# Supplementary material for: Homoleptic Complexes of Heterocyclic Curcuminoids with Mg(II) and Cu(II): First Conformationally Heteroleptic Case, Crystal Structures, and Biological Properties
Source: Molecules. 2023 Feb 2;28(3):1434. doi: 10.3390/molecules28031434 (PMC9919861; doi:10.3390/molecules28031434)
Supplement: Supplementary file 1 [file molecules-28-01434-s001.zip › molecules-2029873-supplementary.pdf]

Supplementary Information

Homoleptic complexes of heterocyclic curcuminoids with Mg and Cu: first conformationally heteroleptic case, crystal structures and biological properties

*William Meza-Morales,[1] Yuritzi Alejo-Osorio,[1] Yair Alvarez-Ricardo,[1] Marco A. Obregón-Mendoza,[1] Juan C. Machado-Rodriguez,[1] Antonino Arenaza-Corona,[1] Rubén A. Toscano,[1] María Teresa Ramírez-Apan,[1] and Raúl G. Enríquez[1]**

1 Instituto de Química, Universidad Nacional Autónoma de México, Circuito Exterior, Ciudad Universitaria, México City, C.P. 07340, México

Corresponding author: *enriquezhabib@gmail.com

Table of Contents

SINGLE CRYSTAL X-RAY DIFFRACTION	1
NUCLEAR MAGNETIC RESONANCE	7
ELECTRON PARAMAGNETIC RESONANCE	37
INFRARED SPECTROSCOPY	40
MASS SPECTROMETRY	49
UV-Vis SPECTROSCOPY	58

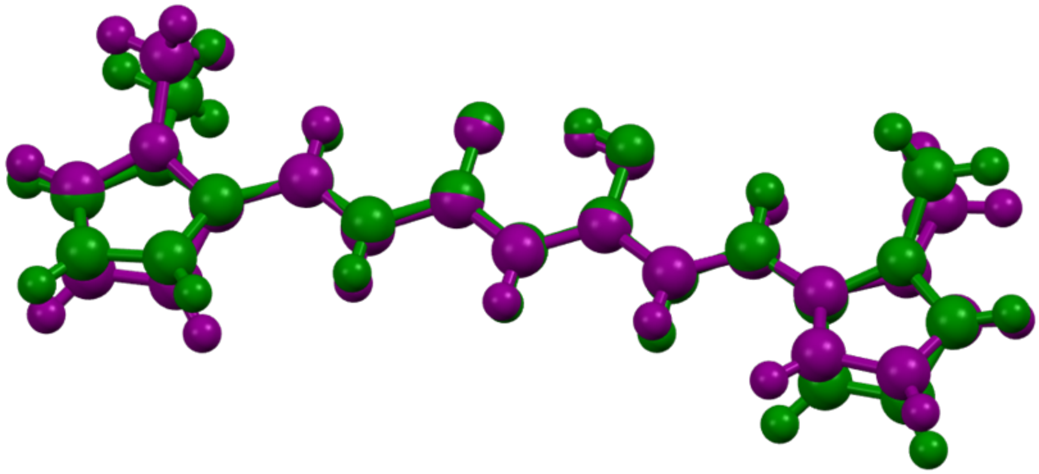

.

Figure S1. Calculated overlay (RMSD = 0.0764) of two polymorphs of ligand **1**.

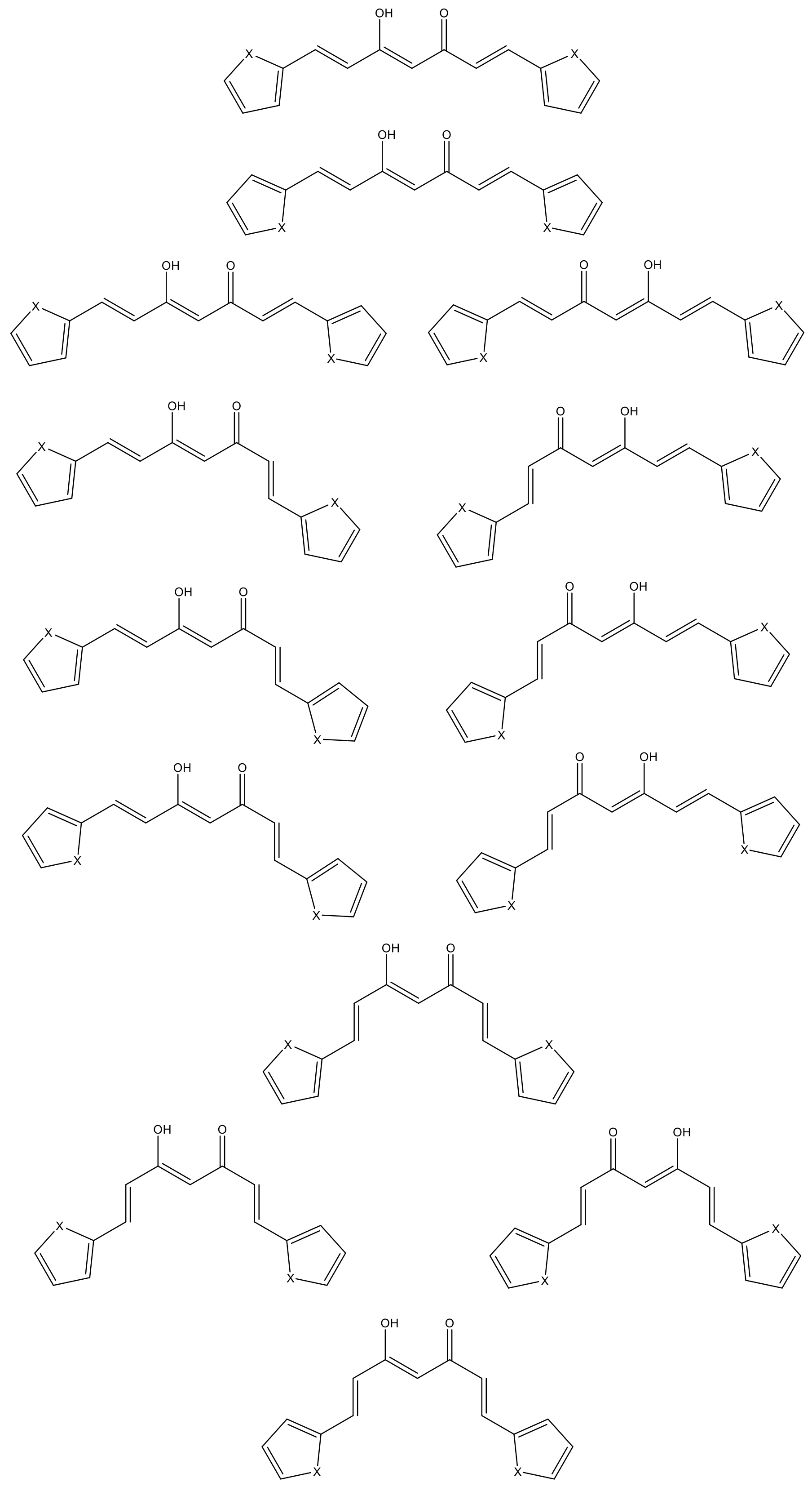

Figure S2. Theoretical extreme conformers for **1-3.**

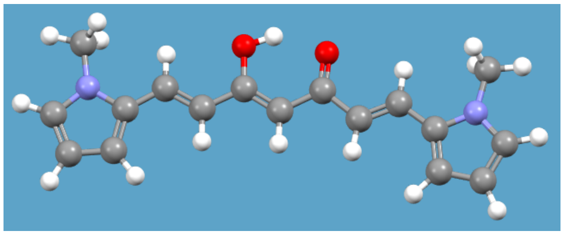

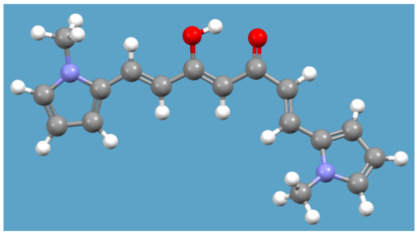

ΔE = 0.0

ΔE = 3.9569

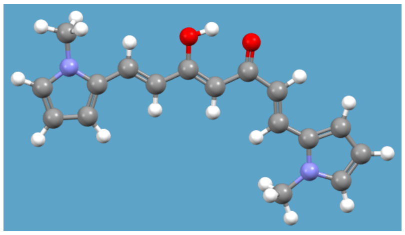

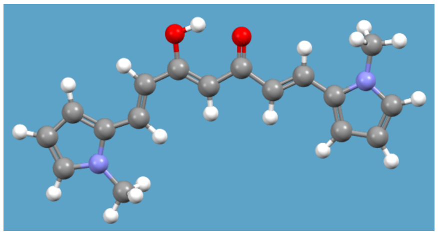

ΔE = 4.7476

ΔE = 6.5320

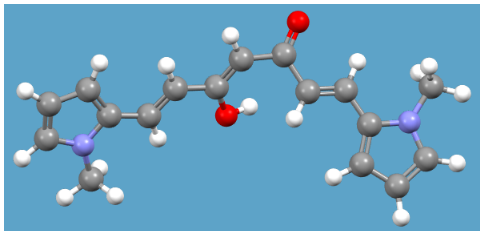

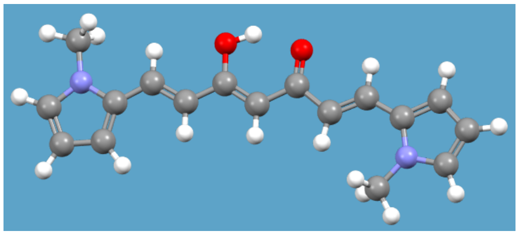

ΔE = 9.4600

ΔE = 10.5589

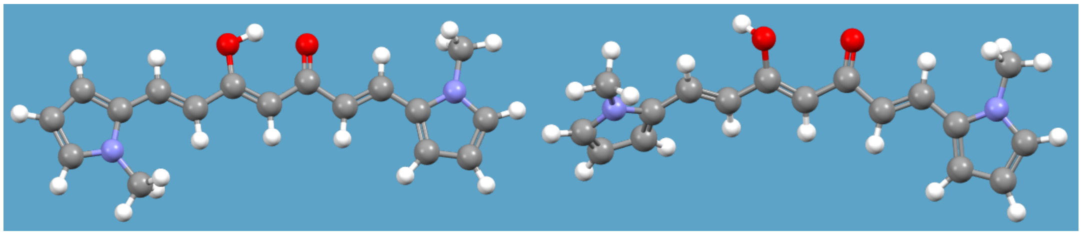

ΔE = 10.6842

ΔE = 11.2770

Figure S3. Structures of the lowest-energy (MMFF94S) conformers of **1.** Relative energies (Kcal/mol).

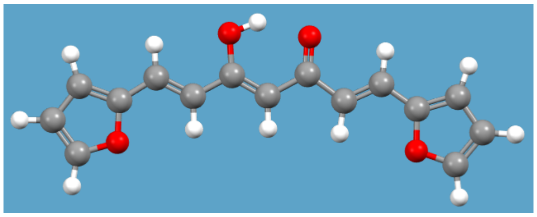

ΔE = 0.0

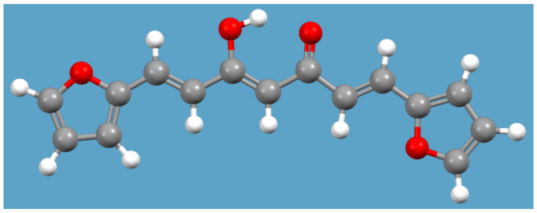

ΔE = 2.1475

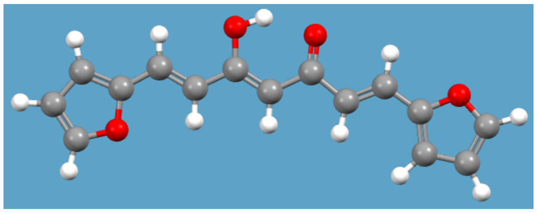

ΔE = 2.6490

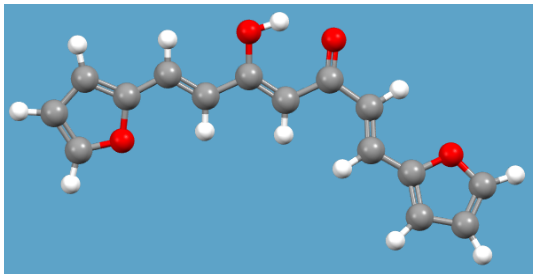

ΔE = 4.0078

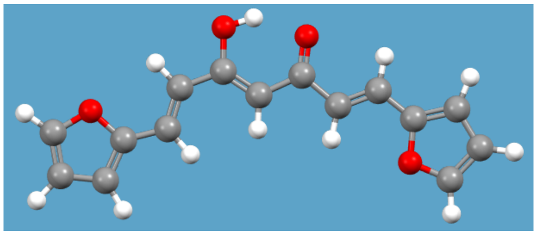

ΔE = 4.8899

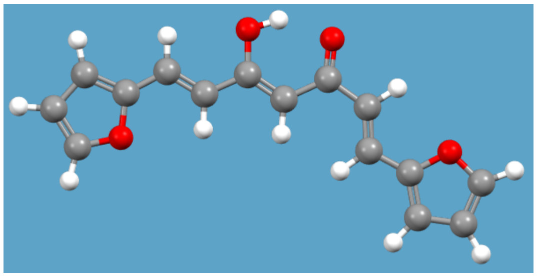

ΔE = 6.0404

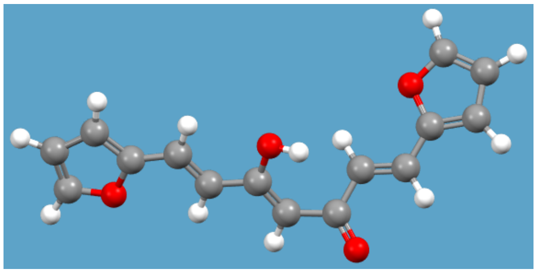

ΔE = 9.6297

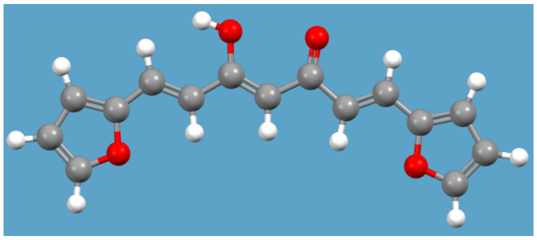

ΔE = 13.3214

Figure S4. Structures of the lowest-energy (MMFF94S) conformers of **2.** Relative energies (Kcal/mol).

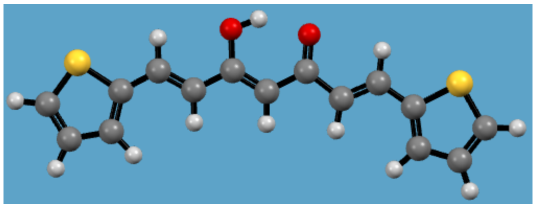

ΔE = 0.0

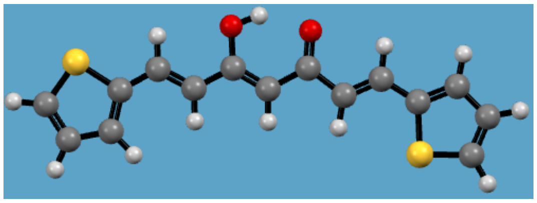

ΔE = 0.7707

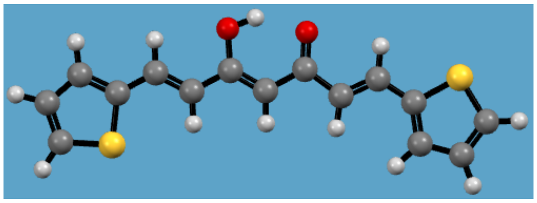

ΔE = 1.0669

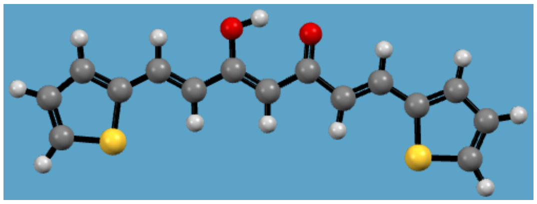

ΔE = 1.8976

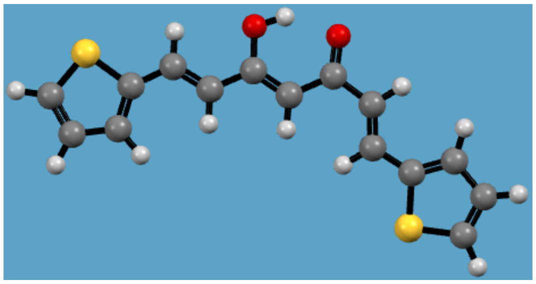

ΔE = 2.5407

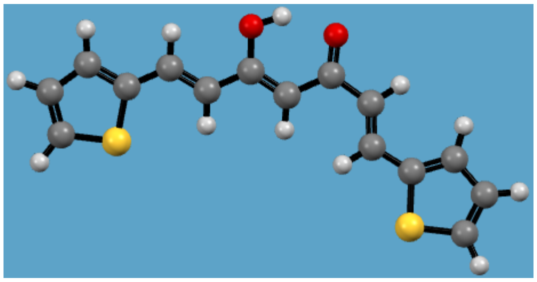

ΔE = 3.6560

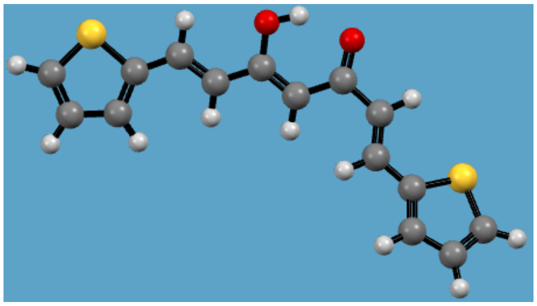

ΔE = 4.6575

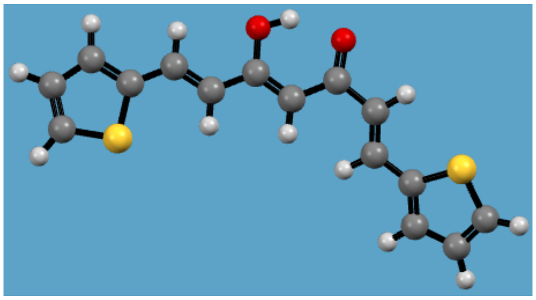

ΔE = 5.7026

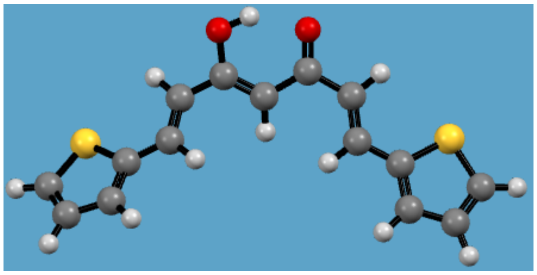

ΔE = 8.1190

Figure S5. Structures of the lowest-energy (MMFF94S) conformers of **3.** Relative energies (Kcal/mol).

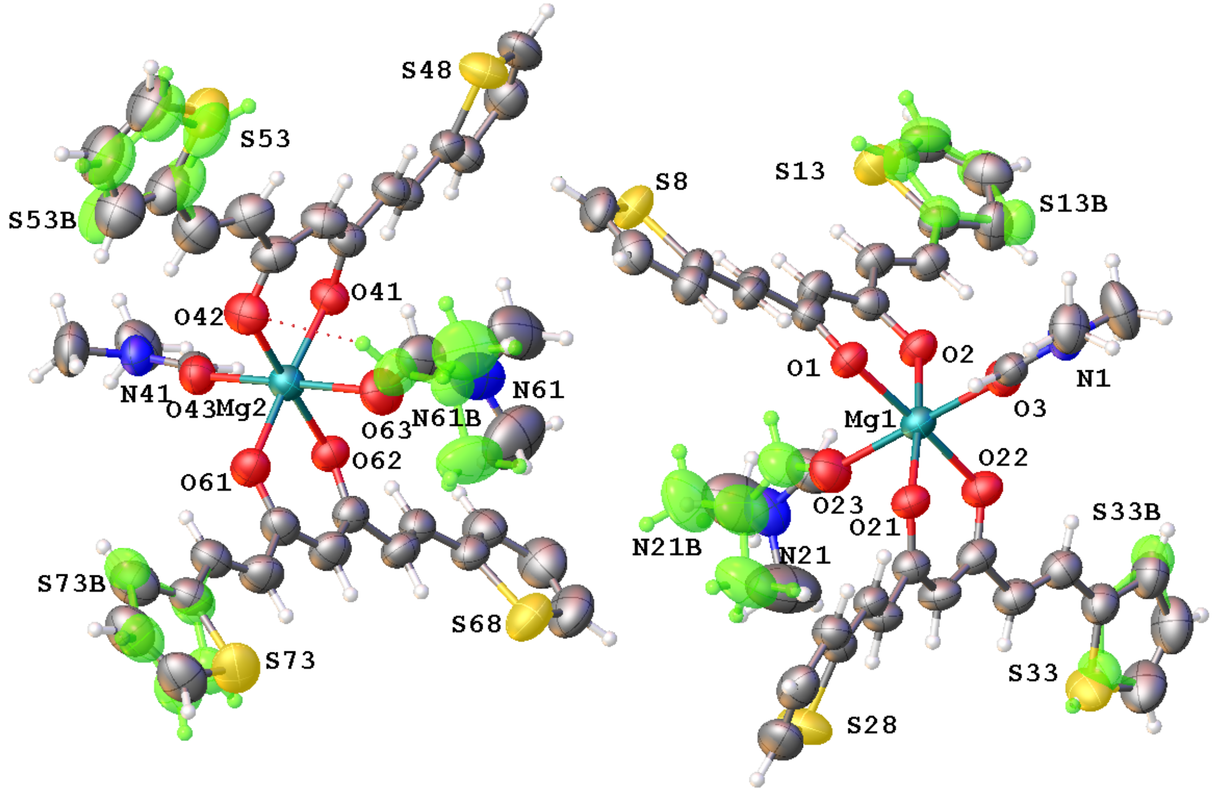

Figure S6. Crystal structures of complex **6**.

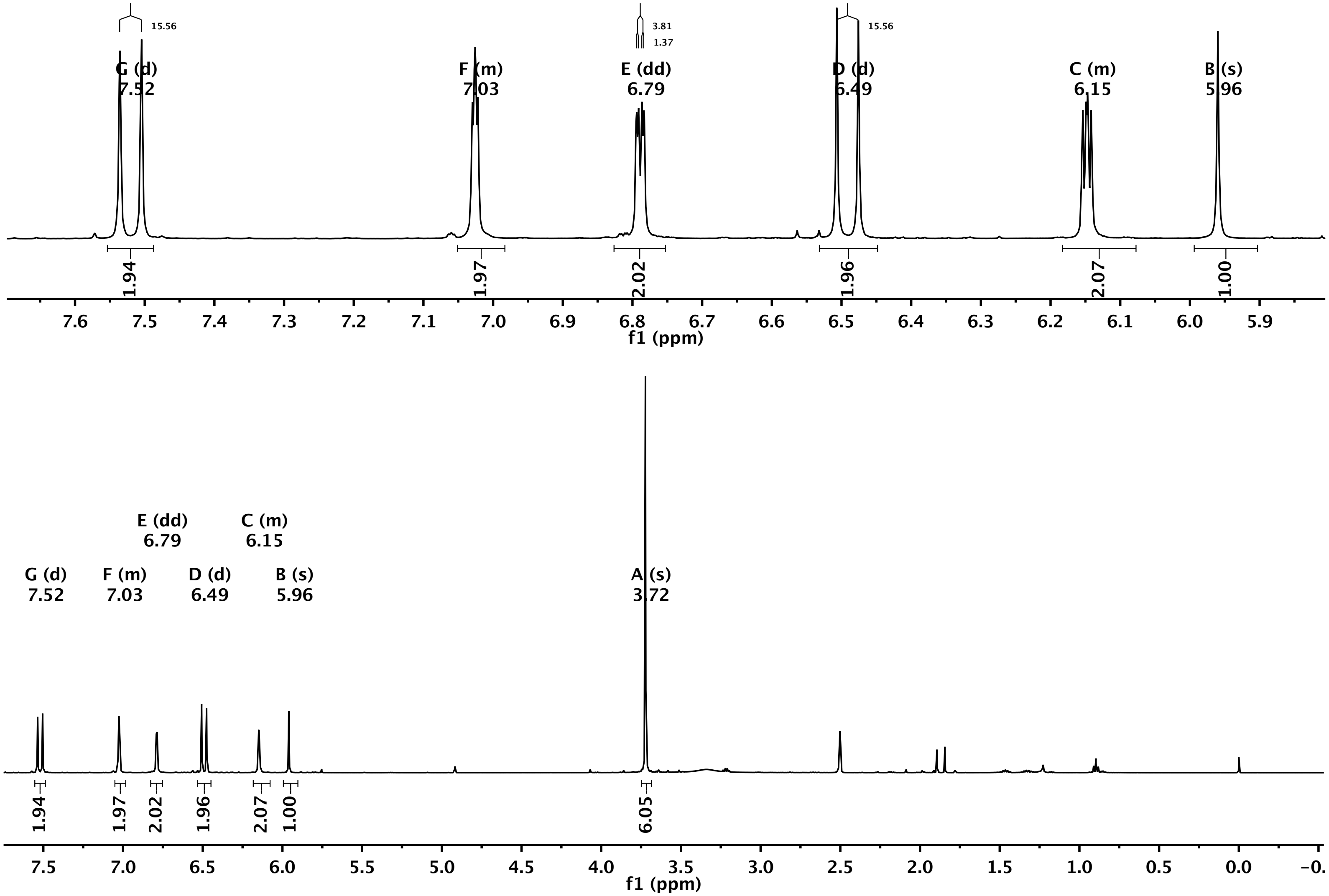

Figure S7. 500 MHz ^{1}H NMR spectrum of N-methyl-pyrCurc.

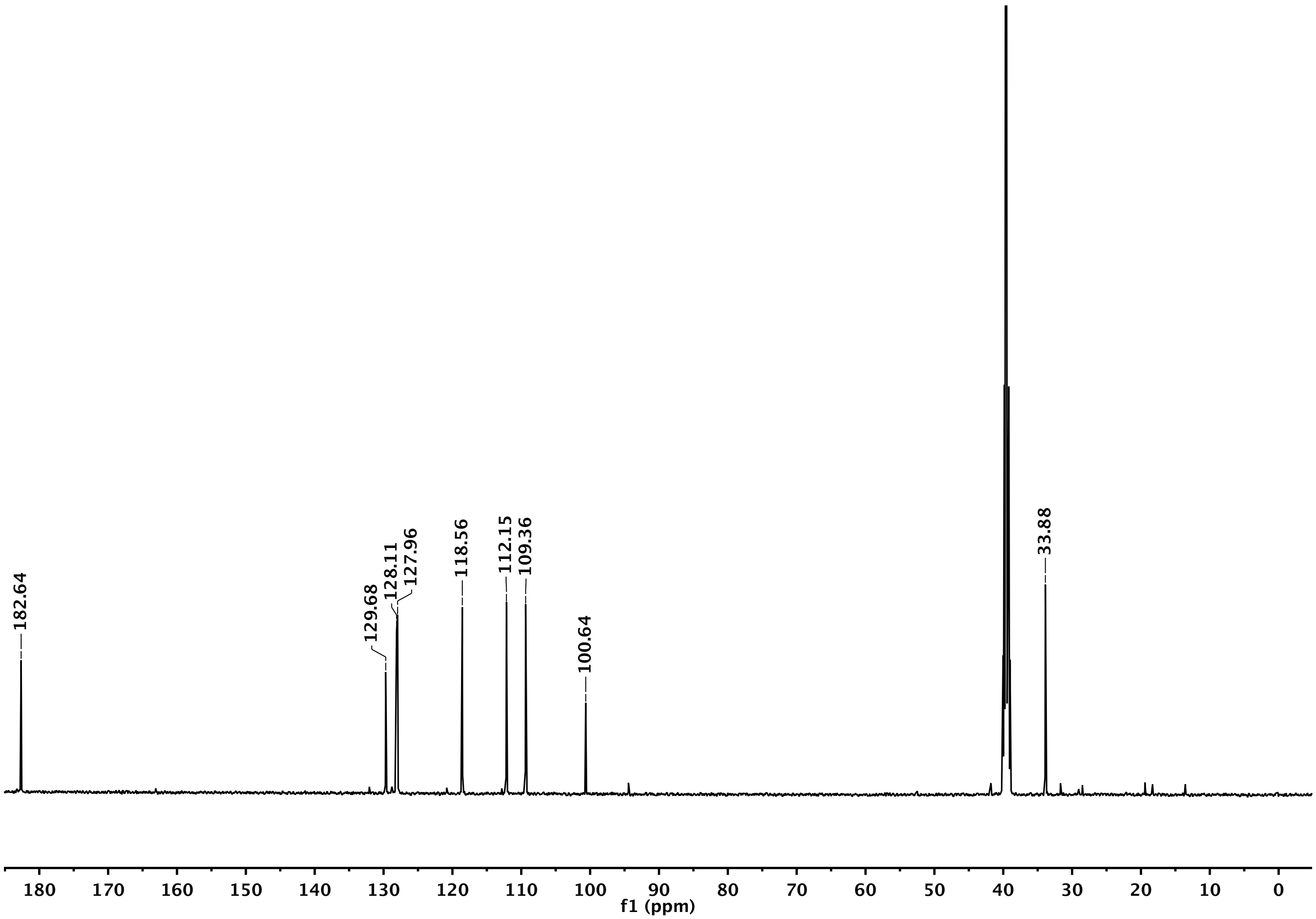

Figure S8. 125 MHz ^{13}C NMR spectrum of N-methyl-pyrCurc.

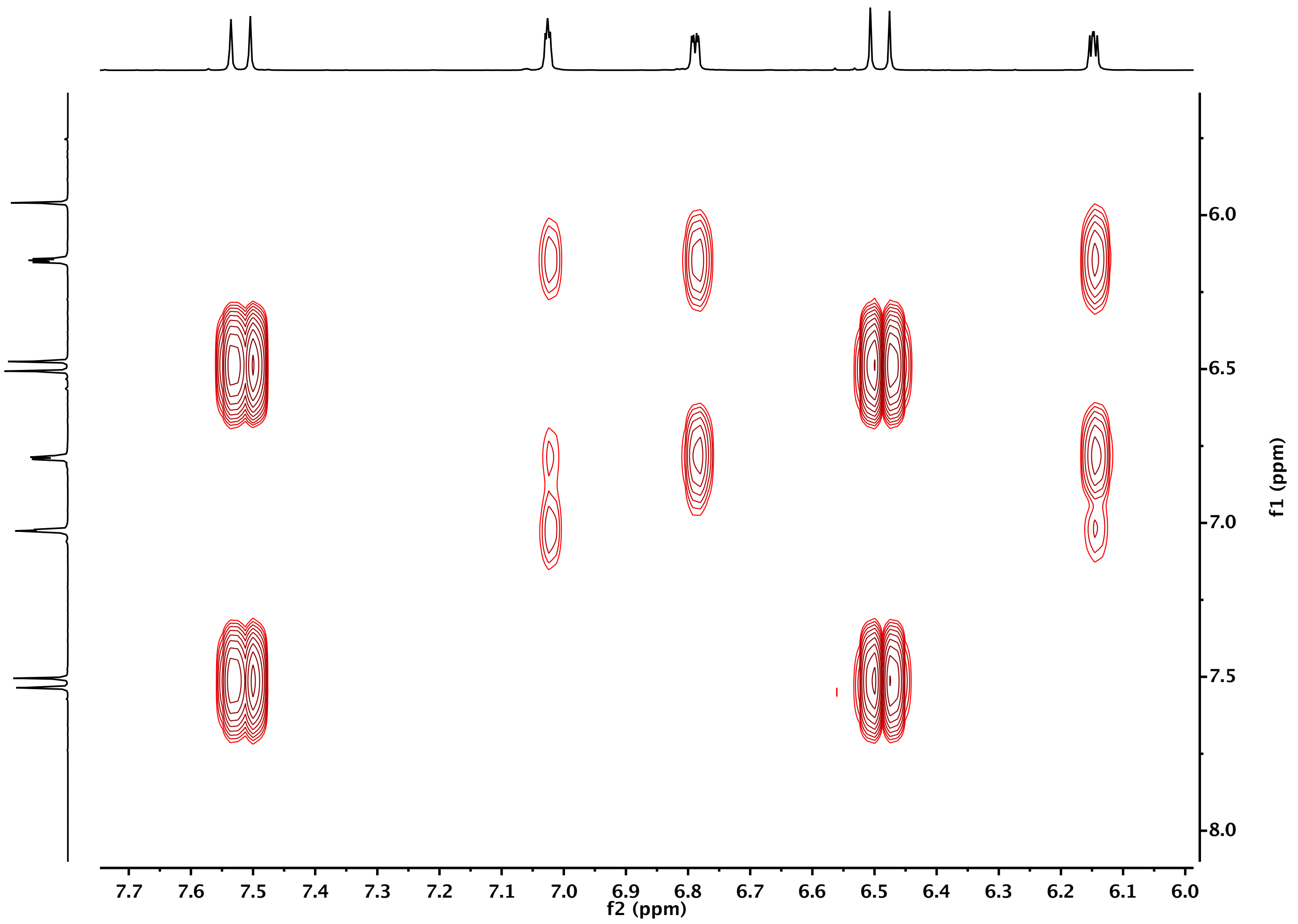

Figure S9. 500 MHz COSY NMR spectrum of N-methyl-pyrCurc.

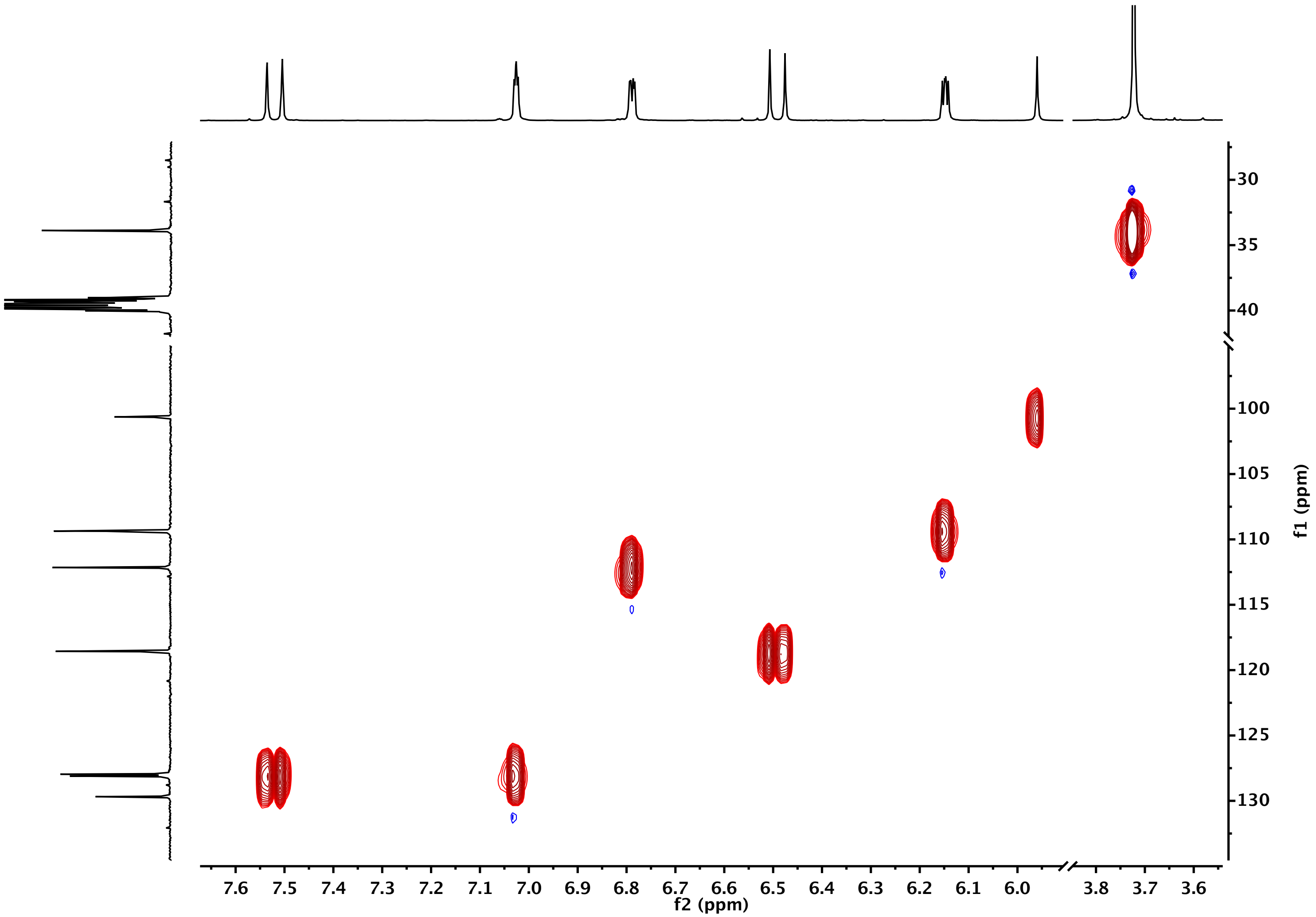

Figure S10. 500 MHz HSQC NMR spectrum of N-methyl-pyrCurc.

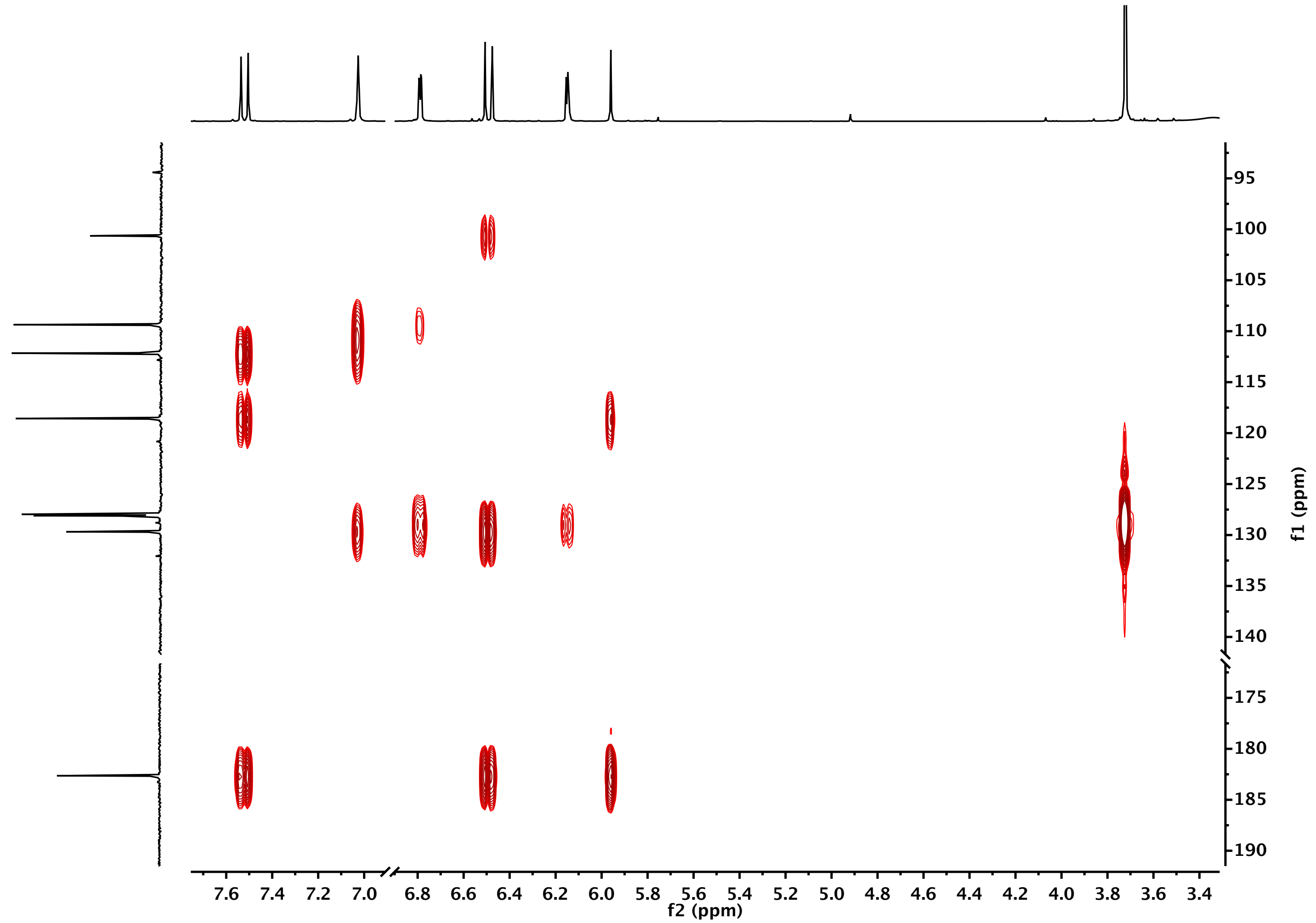

Figure S11. 500 MHz HMBC NMR spectrum of N-methyl-pyrCurc.

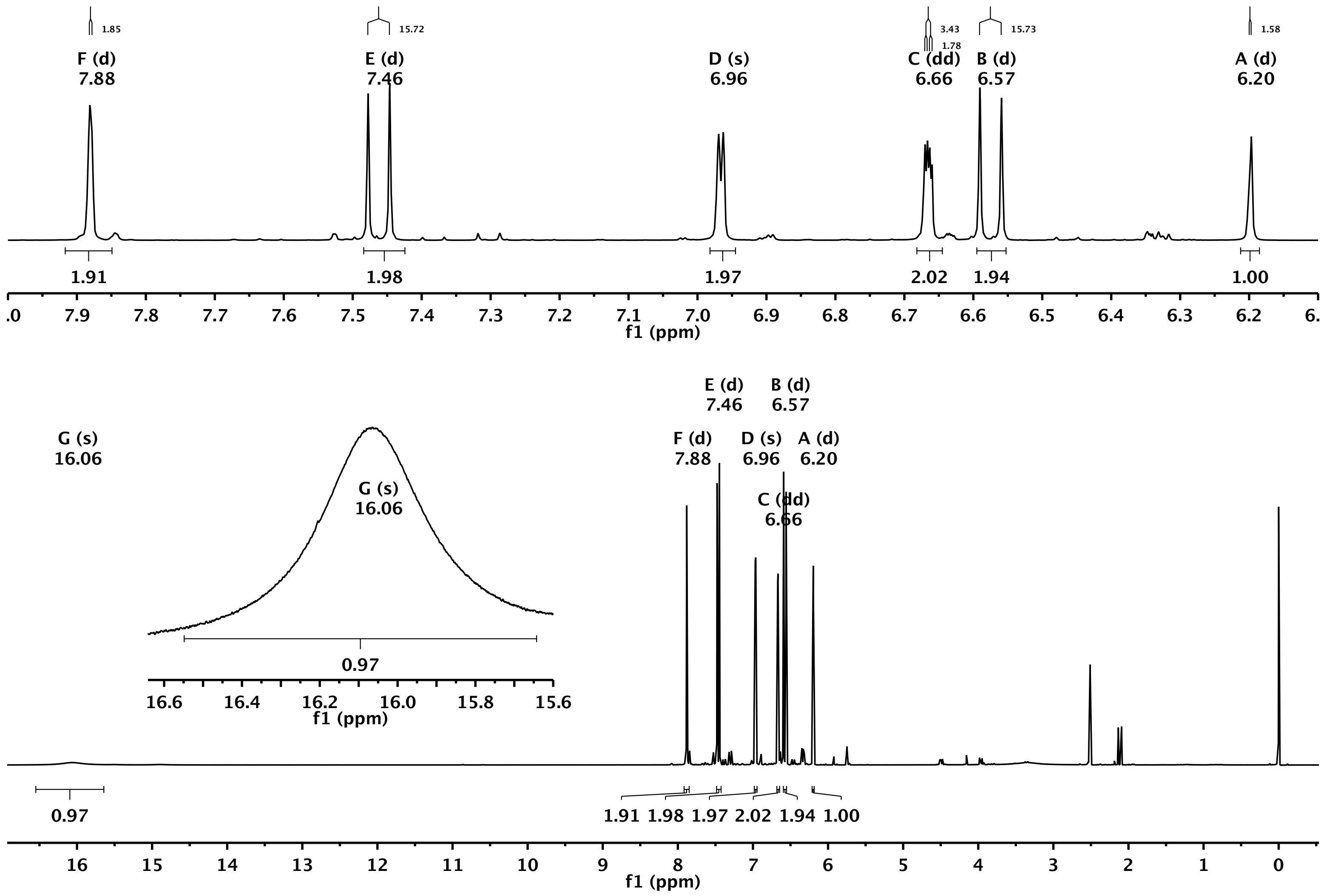

Figure S12. 500 MHz ¹H NMR spectrum of FuranCurc.

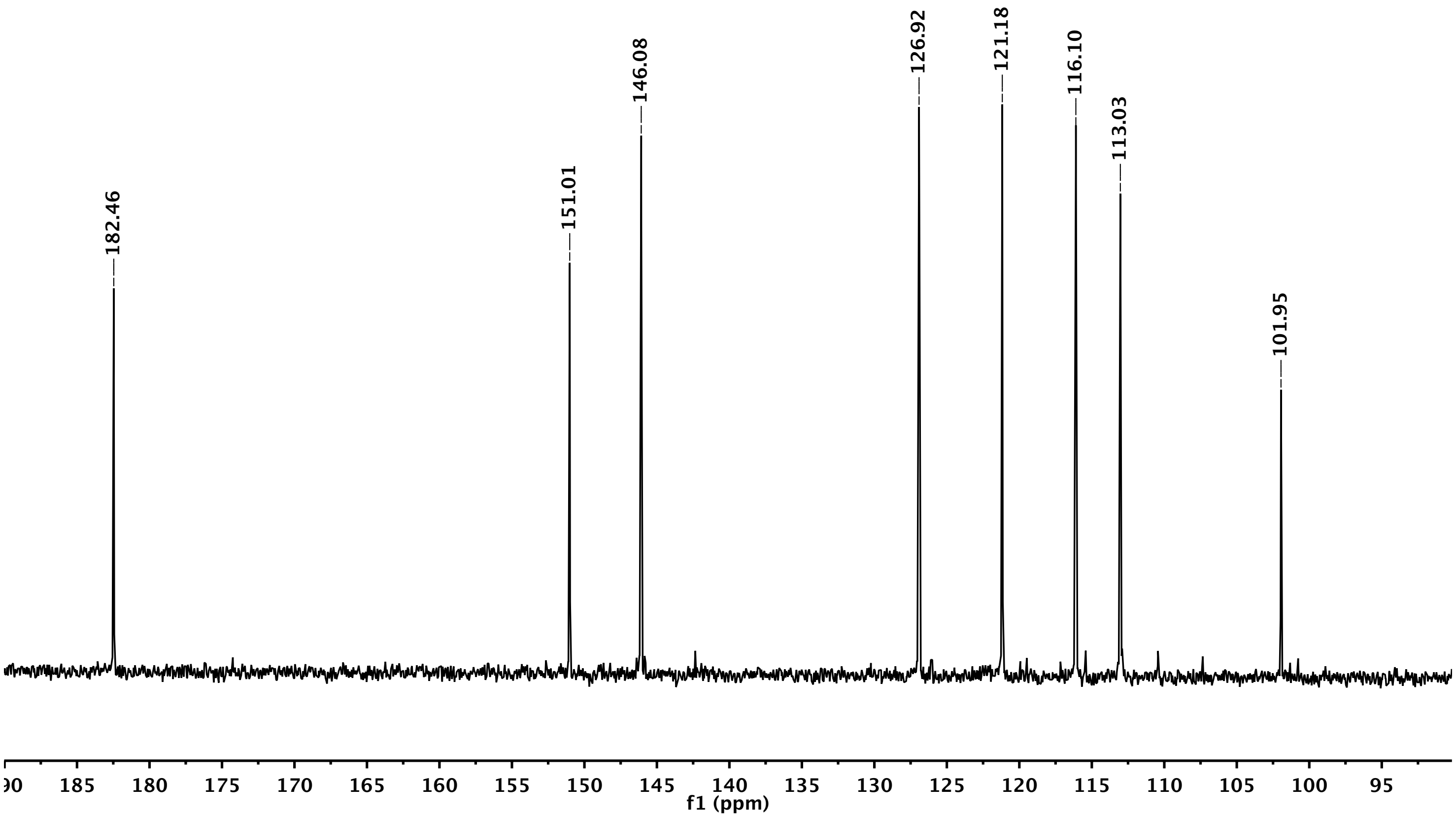

Figure S13. 125 MHz ^{13}C NMR spectrum of FuranCurc.

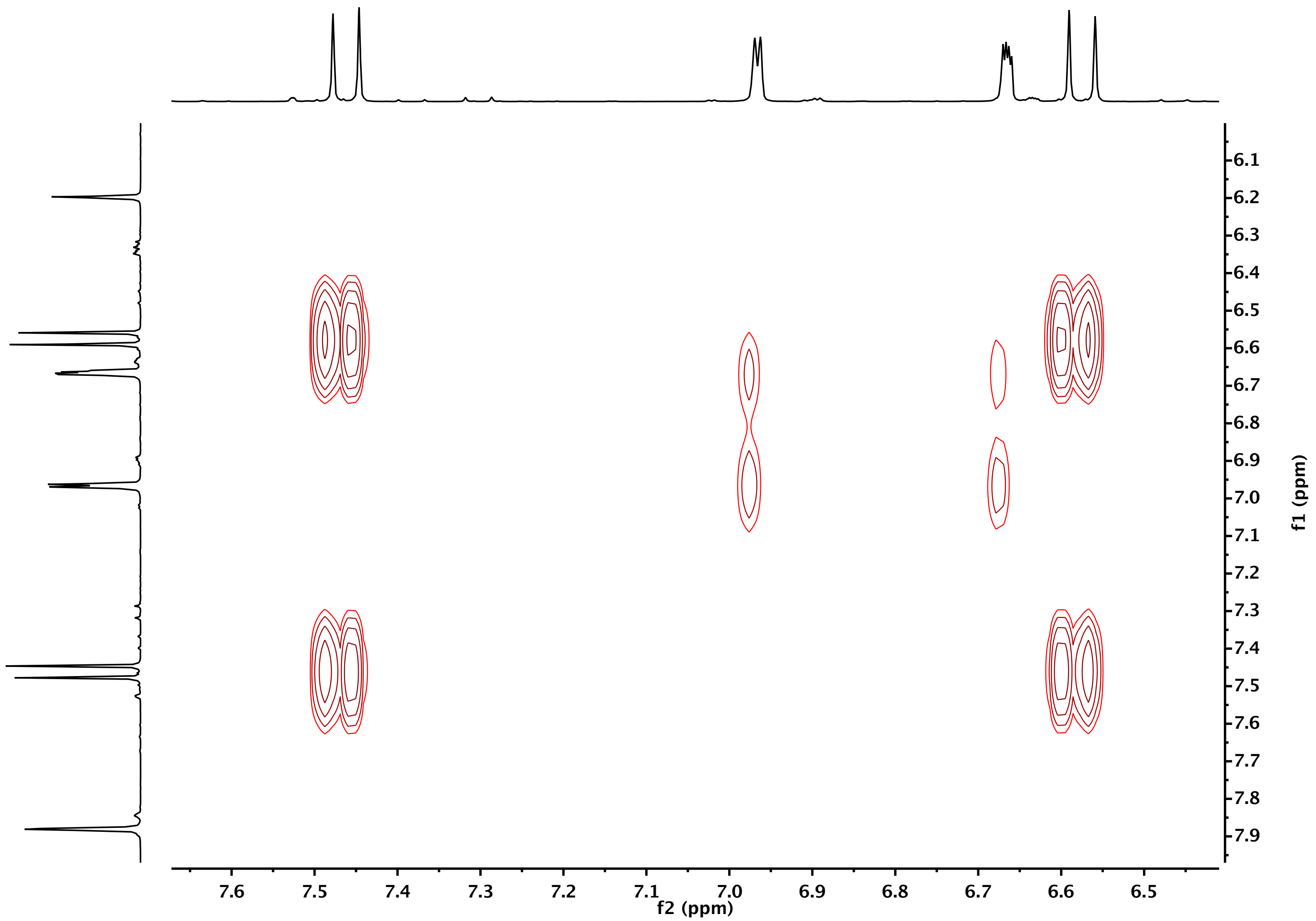

Figure S14. 500 MHz COSY NMR spectrum of FuranCurc.

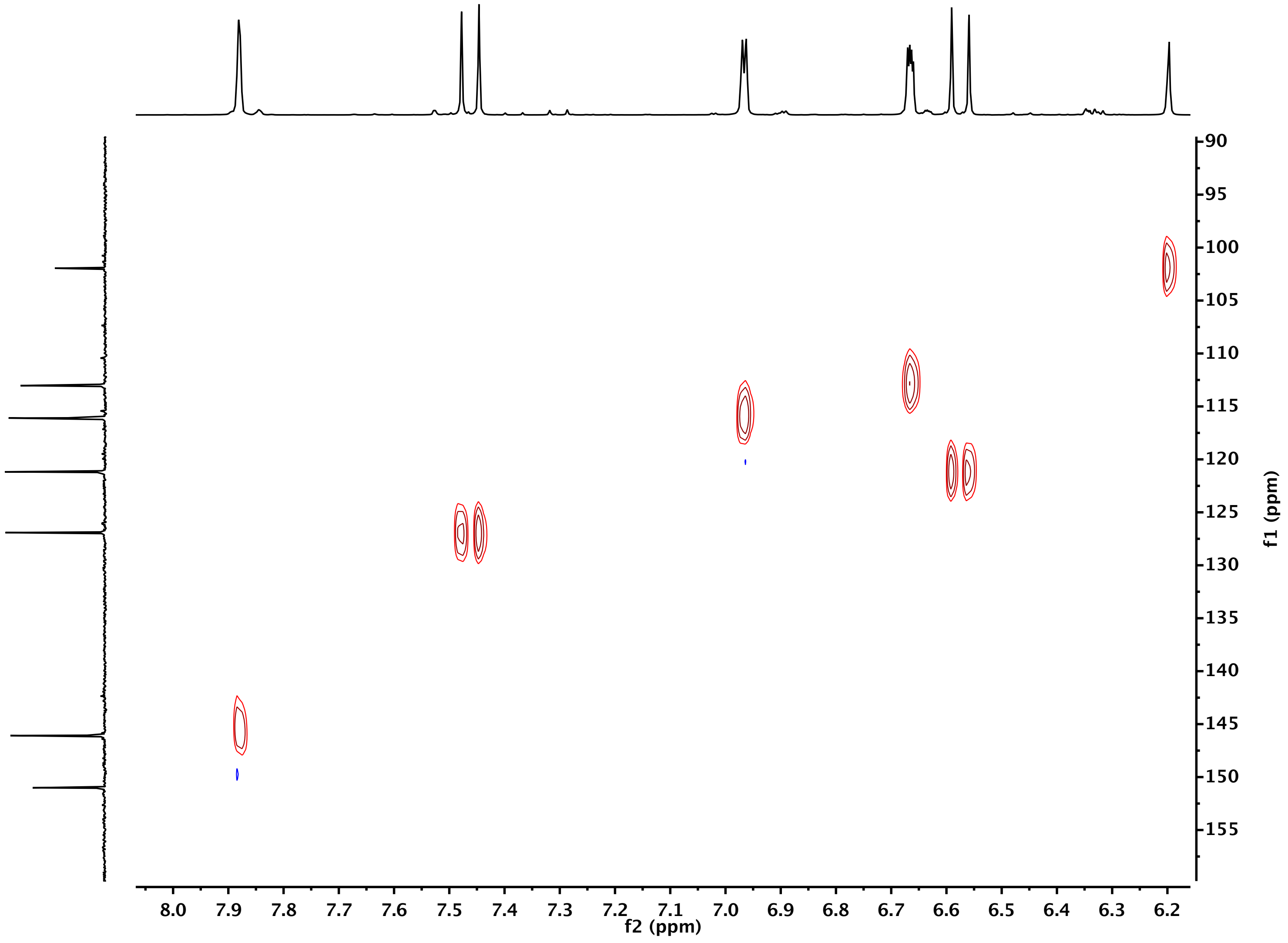

Figure S15. 500 MHz HSQC NMR spectrum of FuranCurc.

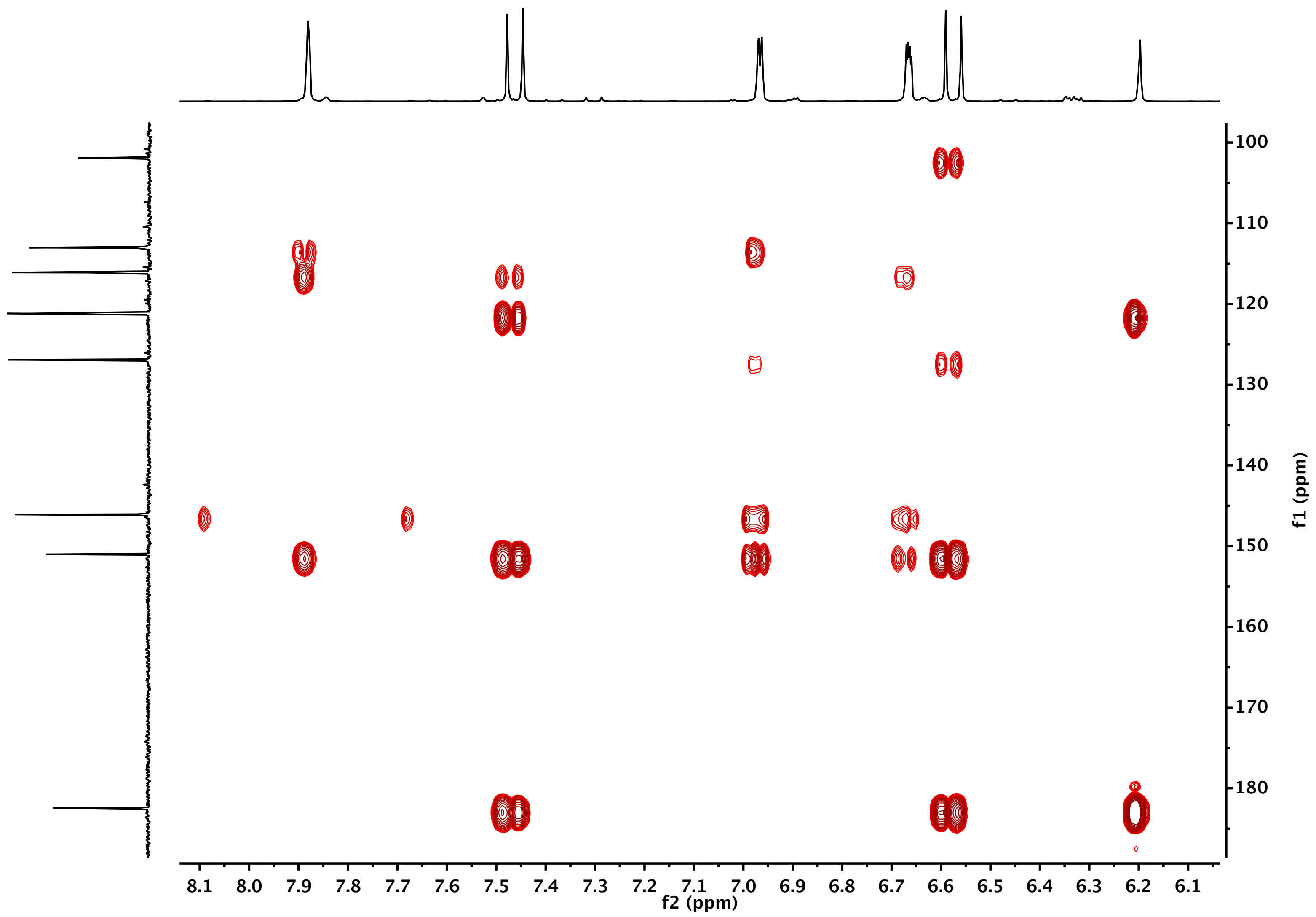

Figure S16. 500 MHz HMBC NMR spectrum of FuranCurc.

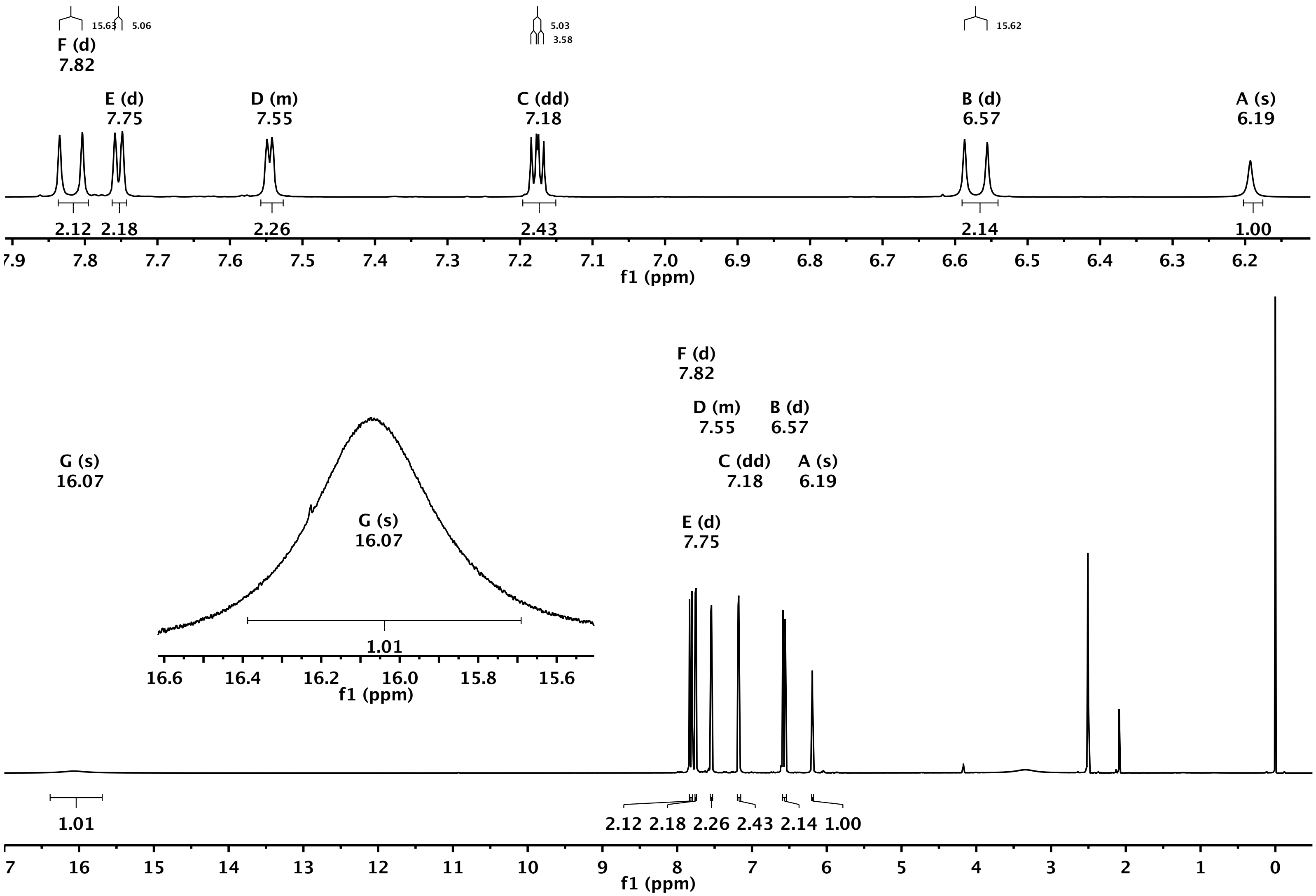

Figure S17. 500 MHz ^{1}H NMR spectrum of ThiopheneCurc.

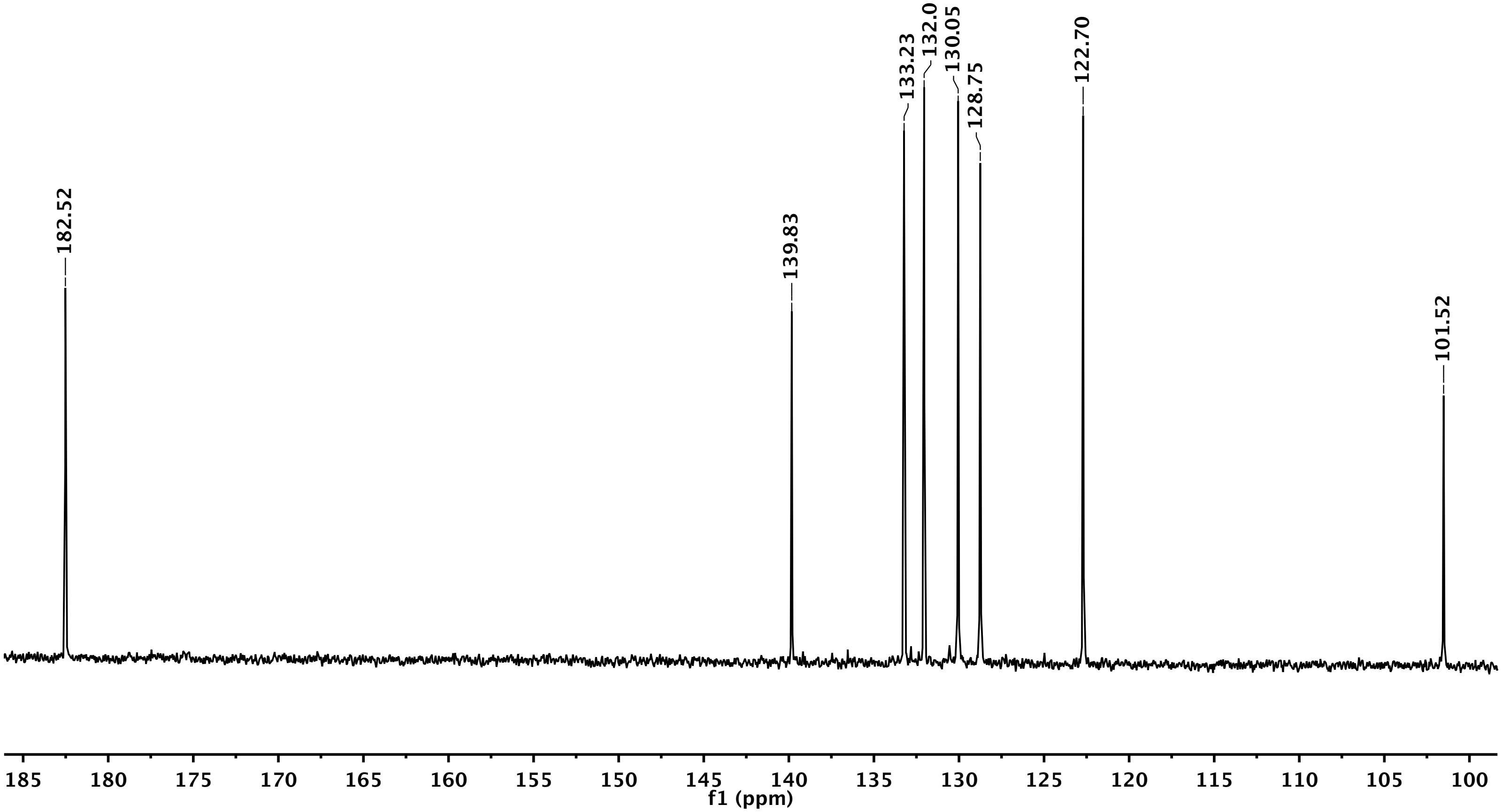

Figure S18. 125 MHz ^{13}C NMR spectrum of ThiopheneCurc.

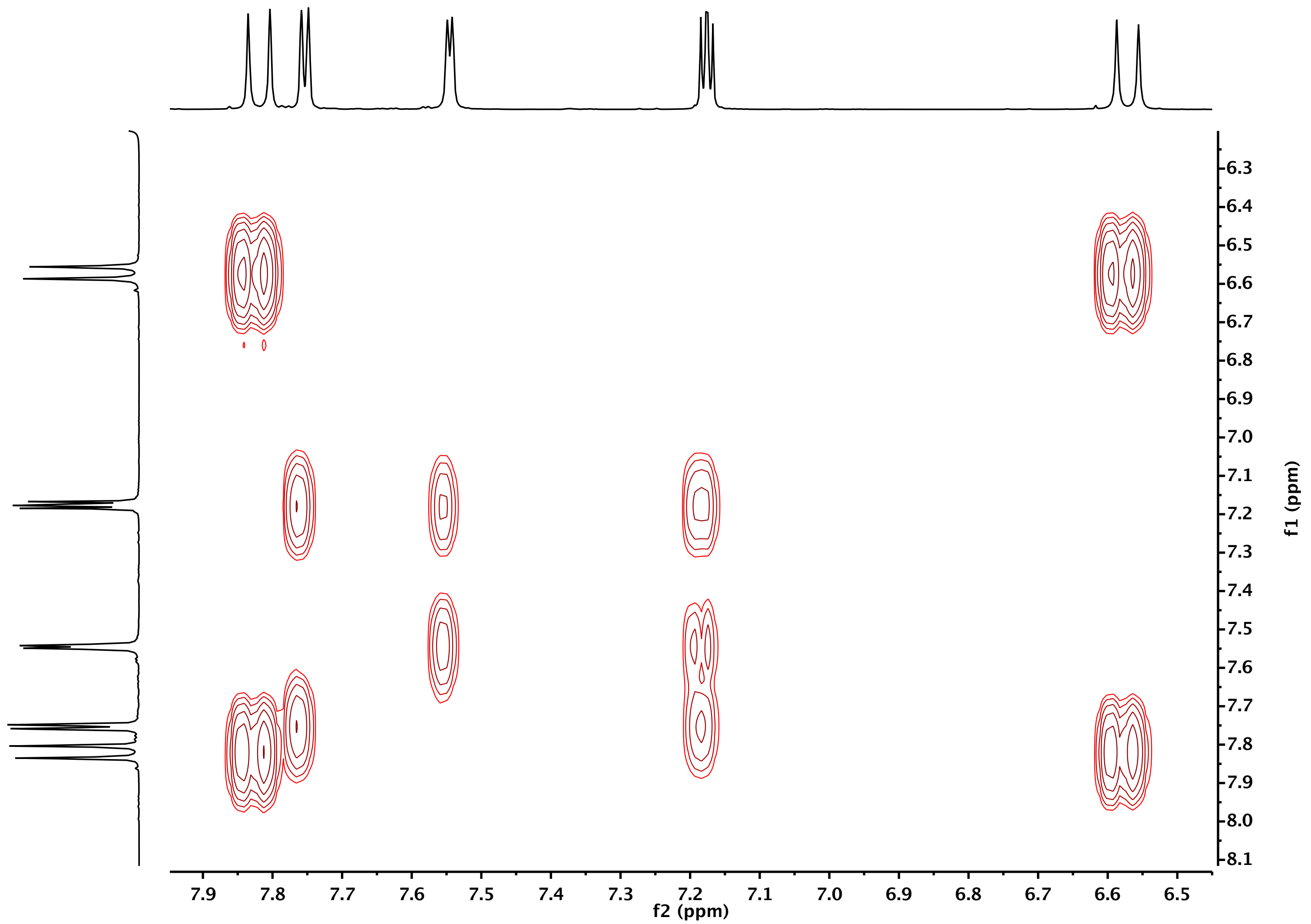

Figure S19. 500 MHz COSY NMR spectrum of ThiopheneCurc.

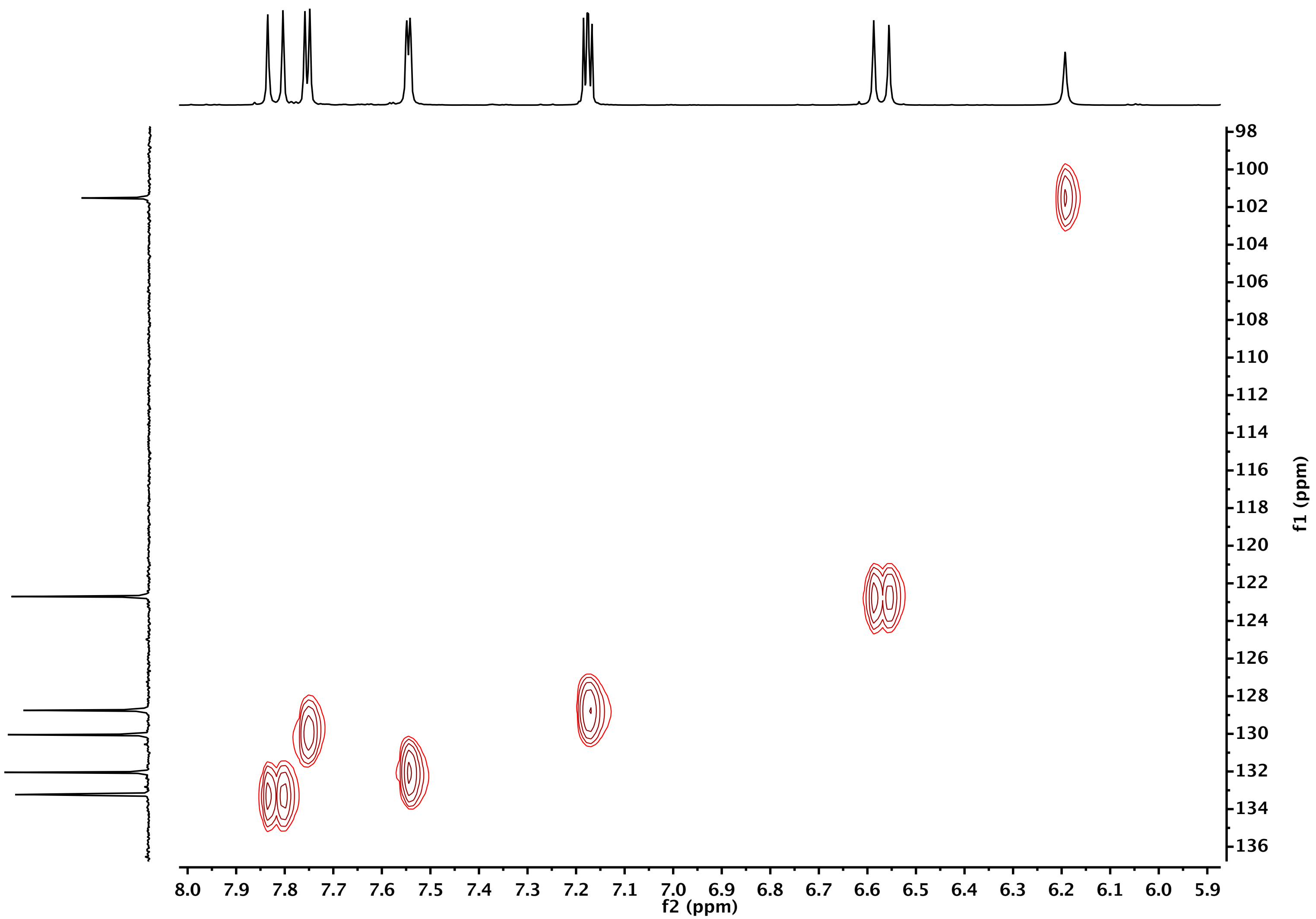

Figure S20. 500 MHz HSQC NMR spectrum of ThiopheneCurc.

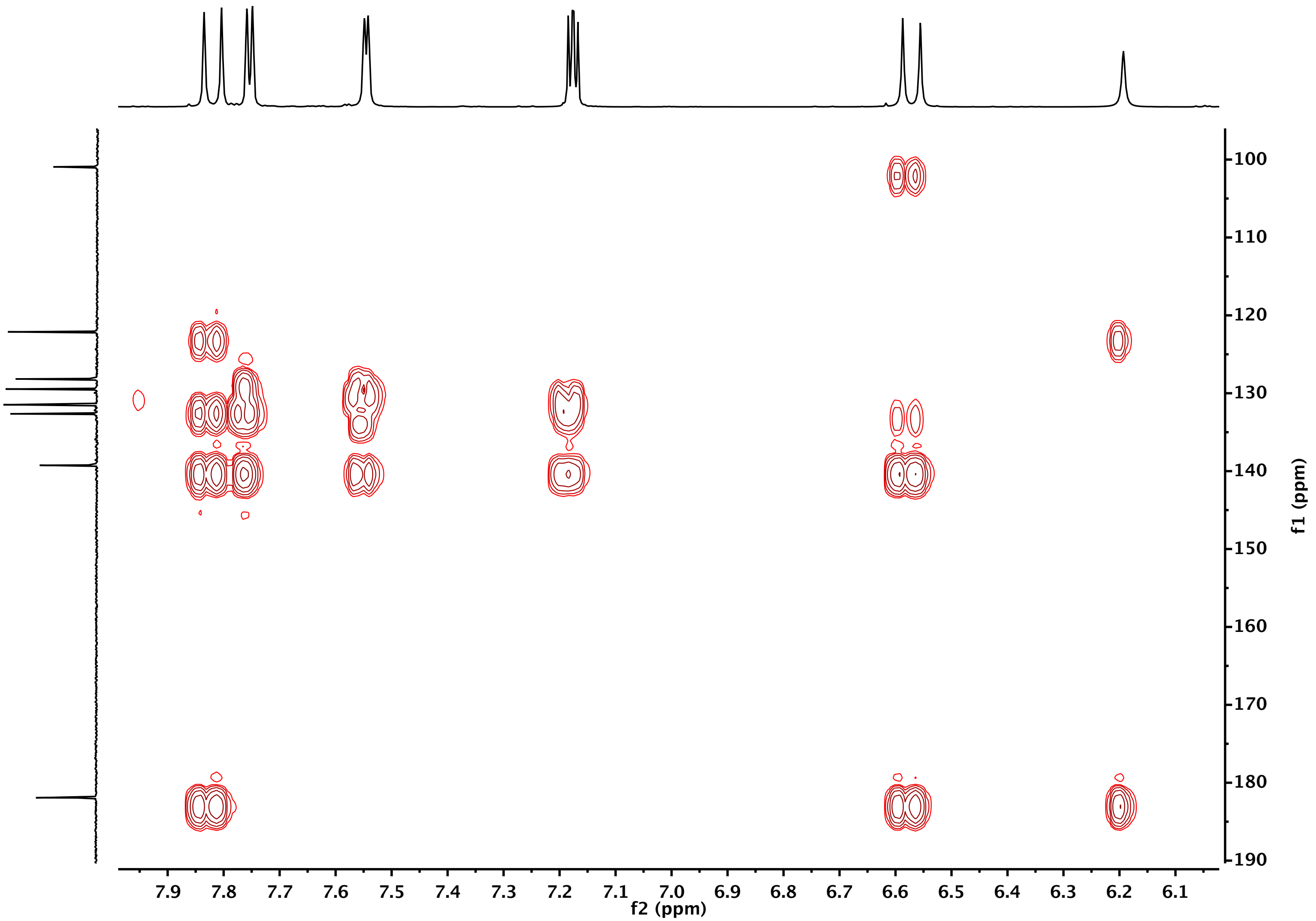

Figure S21. 500 MHz HMBC NMR spectrum of ThiopheneCurc.

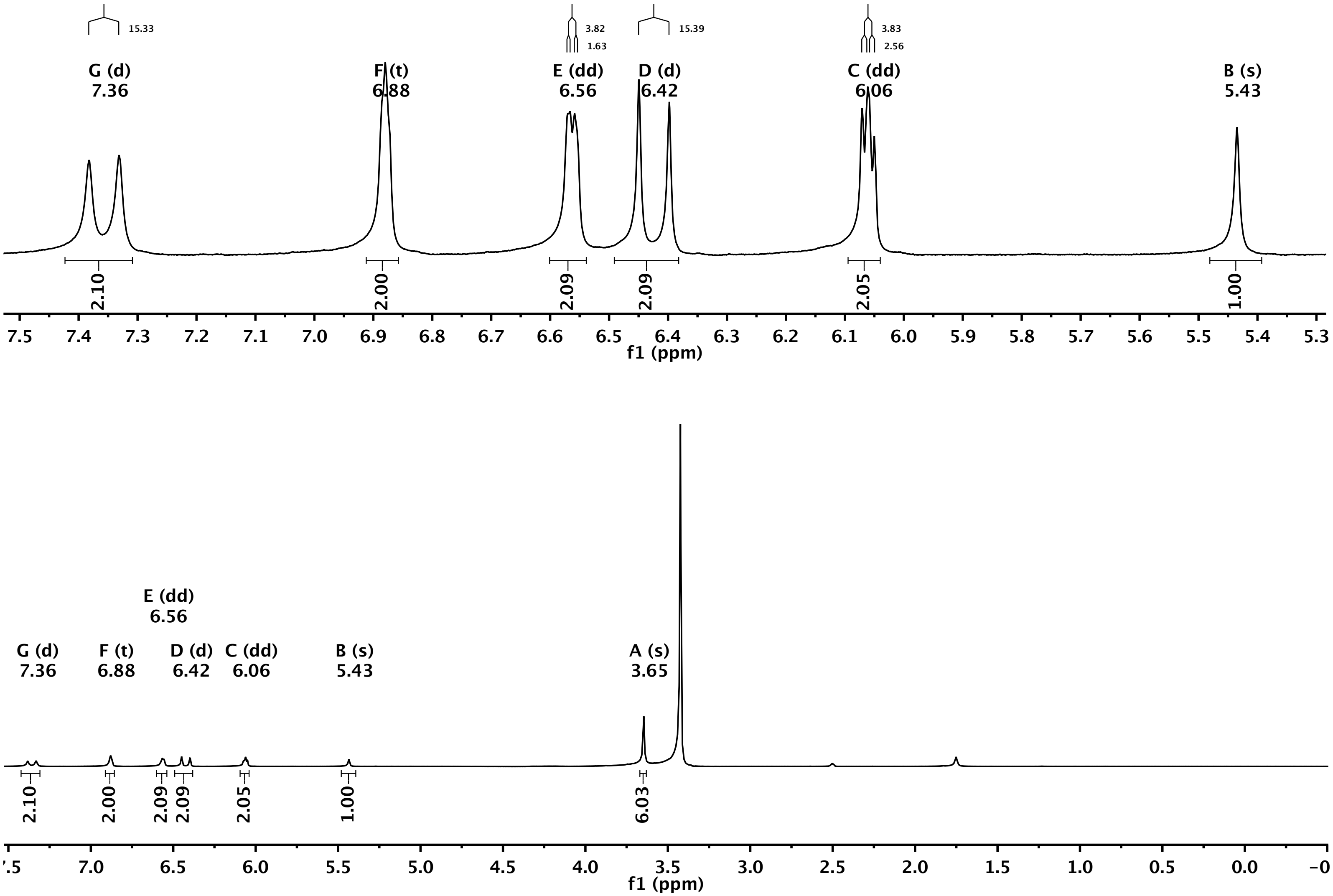

Figure S22. 500 MHz ^{1}H NMR spectrum of N-methyl-pyrCurc-Mg.

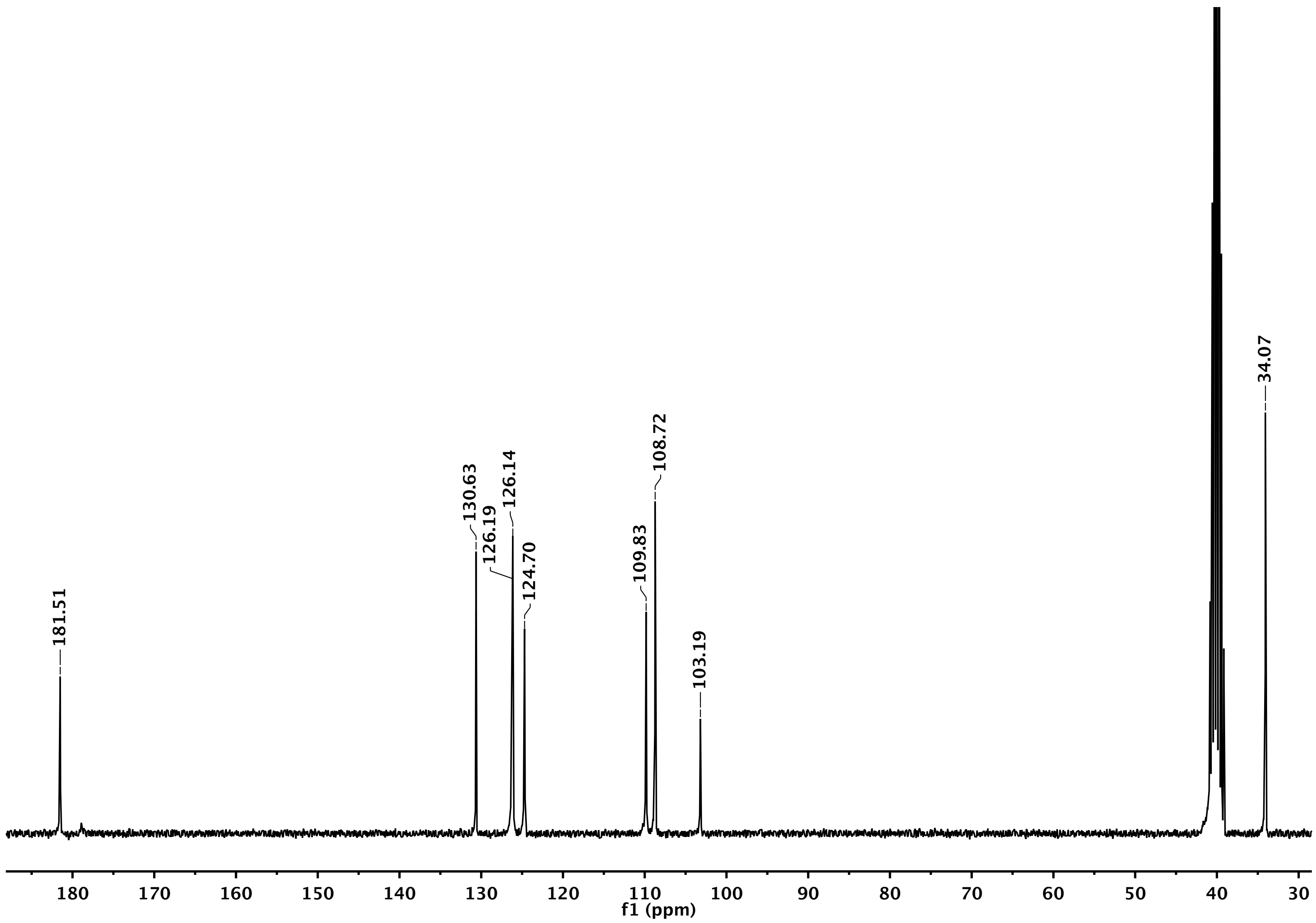

Figure S23. 125 MHz ^{13}C NMR spectrum of N-methyl-pyrCurc-Mg.

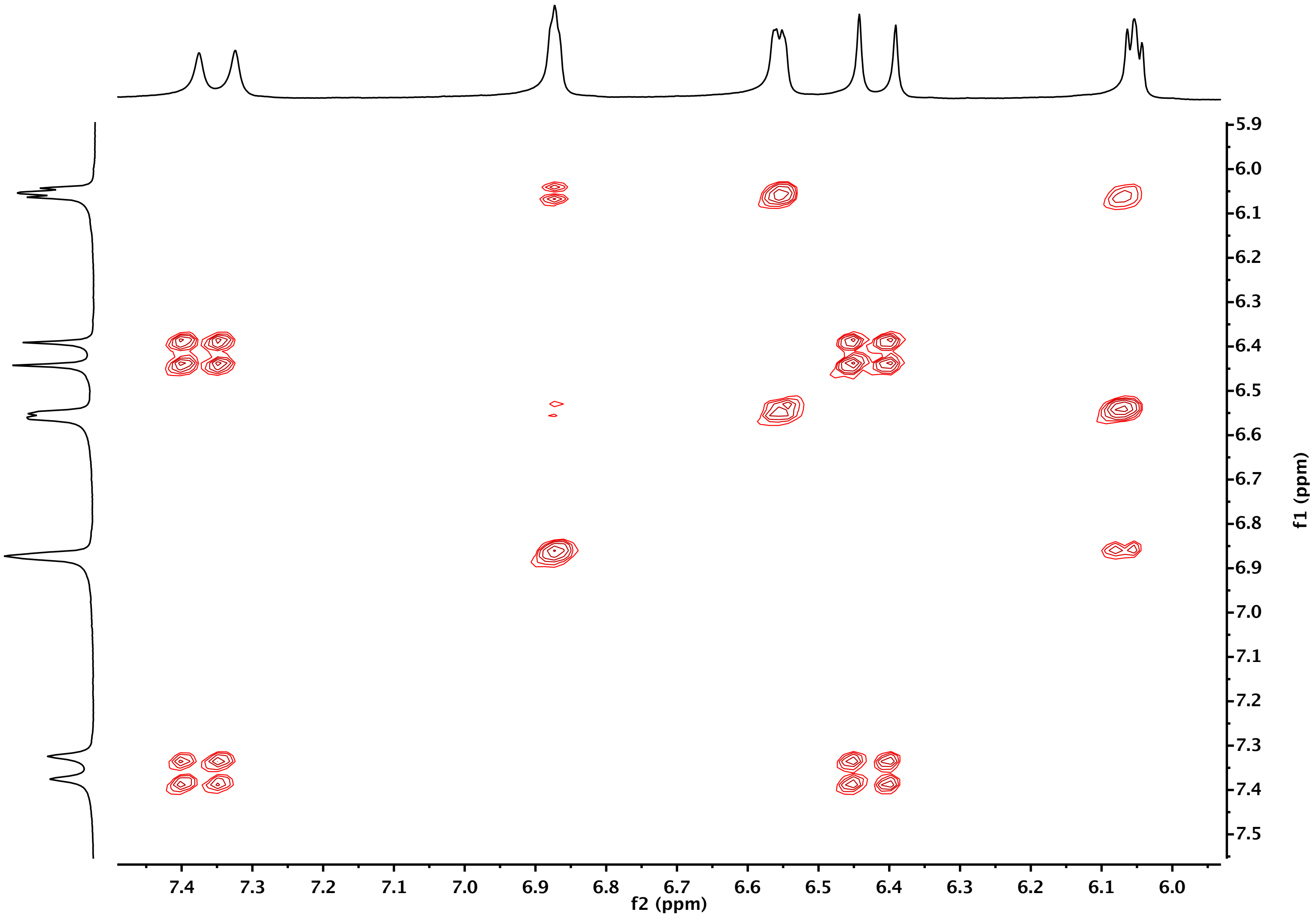

Figure S24. 500 MHz COSY NMR spectrum of N-methyl-pyrCurc-Mg.

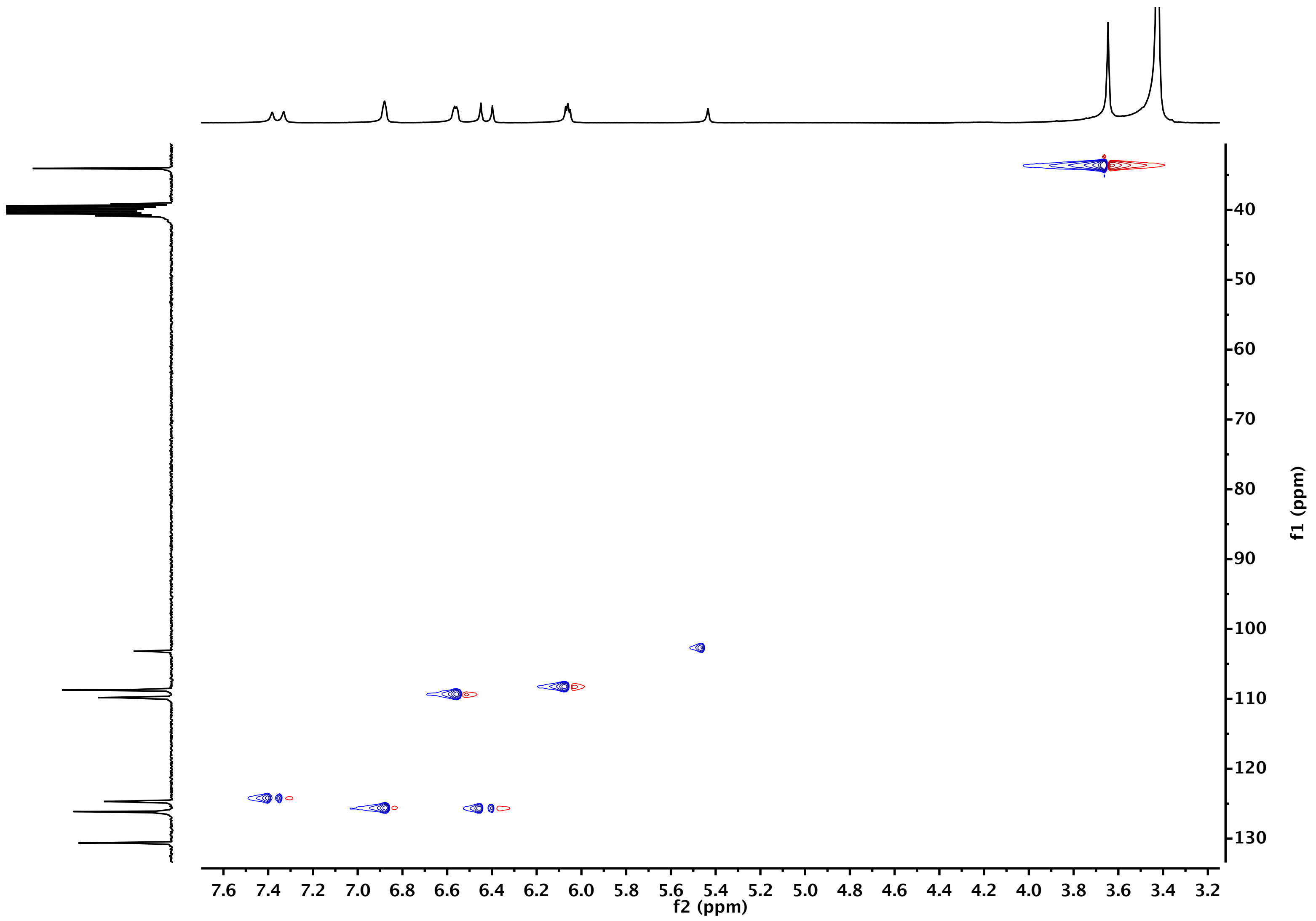

Figure S25. 500 MHz HSQC NMR spectrum of N-methyl-pyrCurc-Mg.

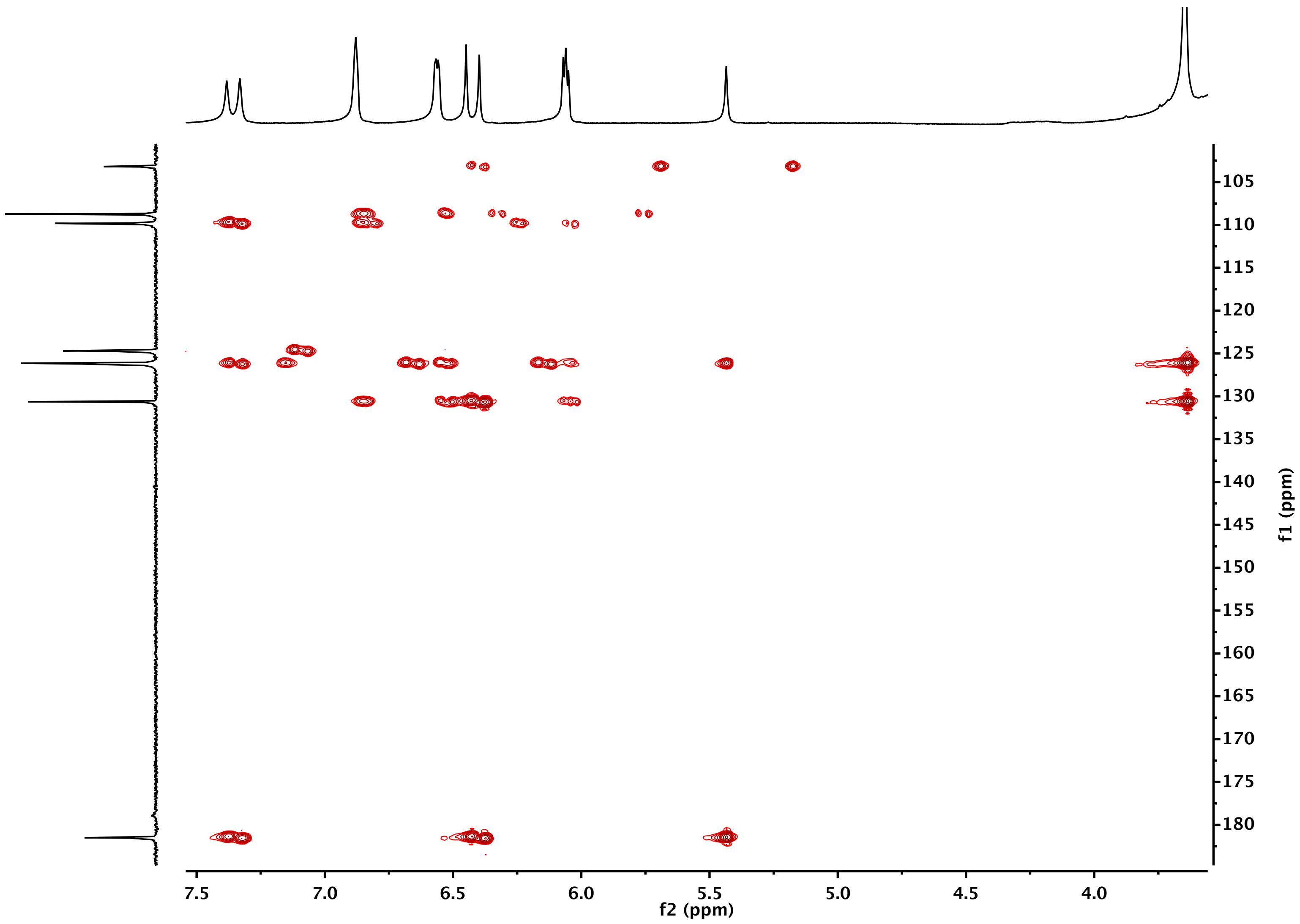

Figure S26. 500 MHz HMBC NMR spectrum of N-methyl-pyrCurc-Mg.

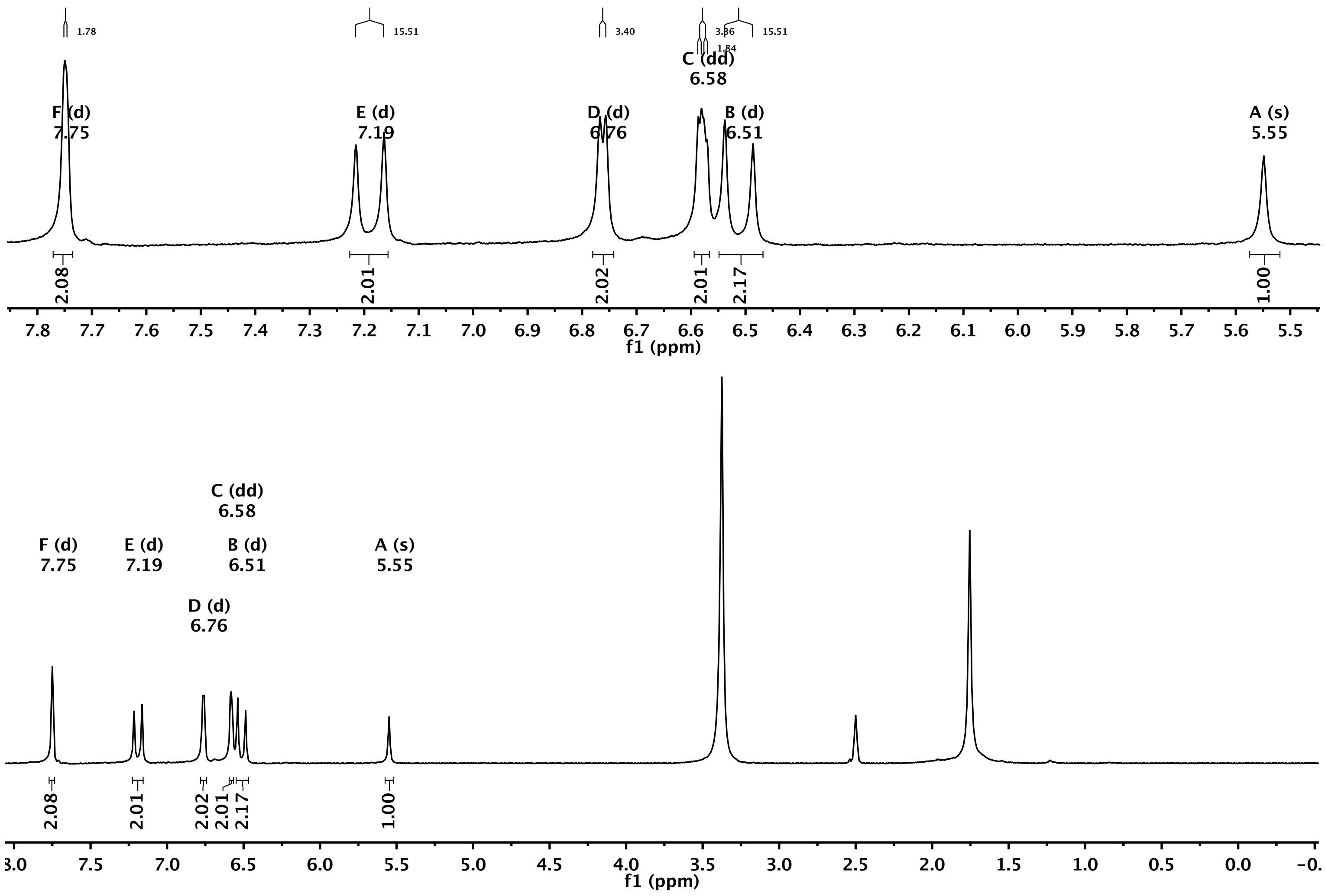

Figure S27. 500 MHz ^{1}H NMR spectrum of FuranCurc-Mg.

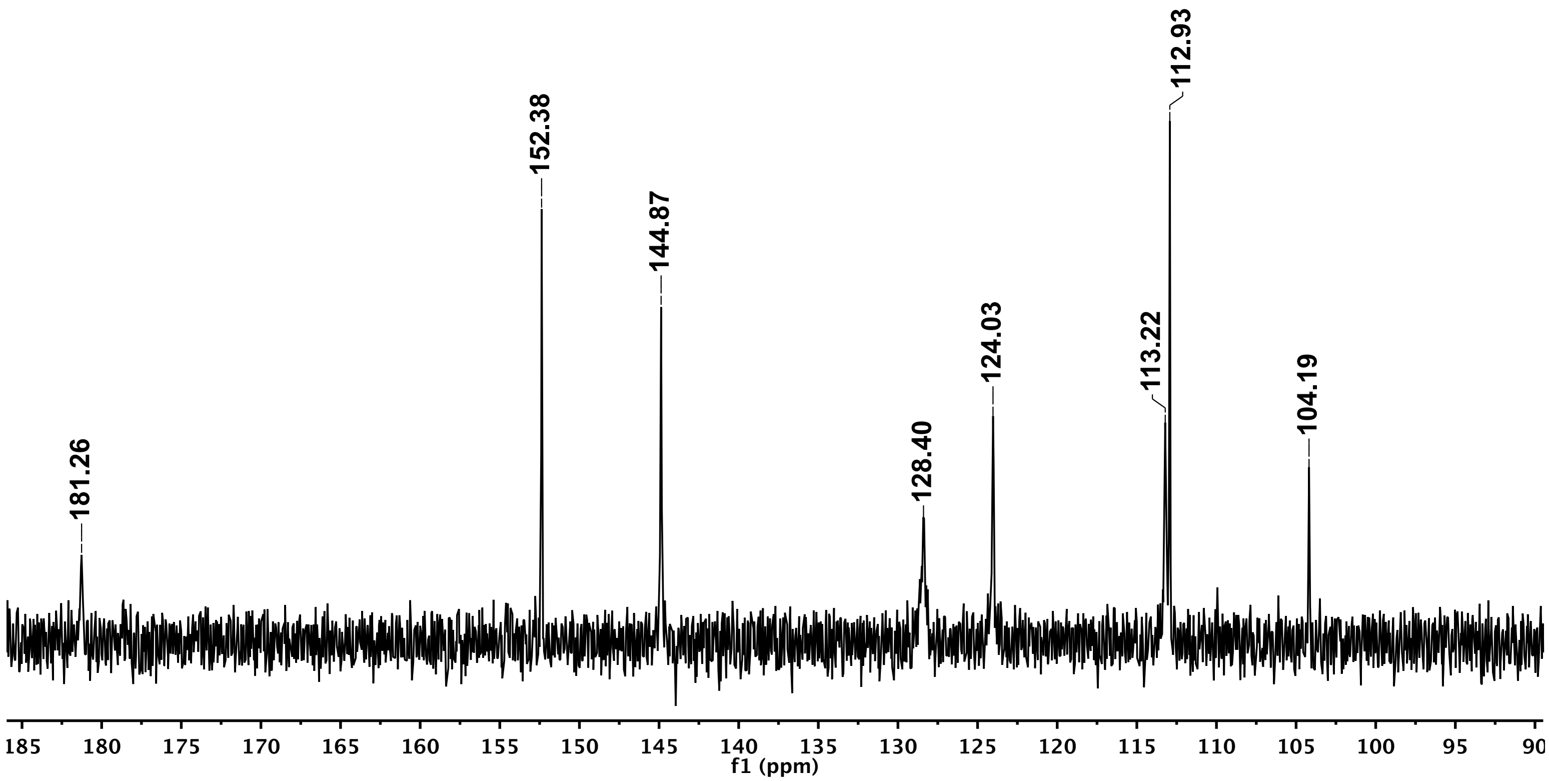

Figure S28. 125 MHz ^{13}C NMR spectrum of FuranCurc-Mg.

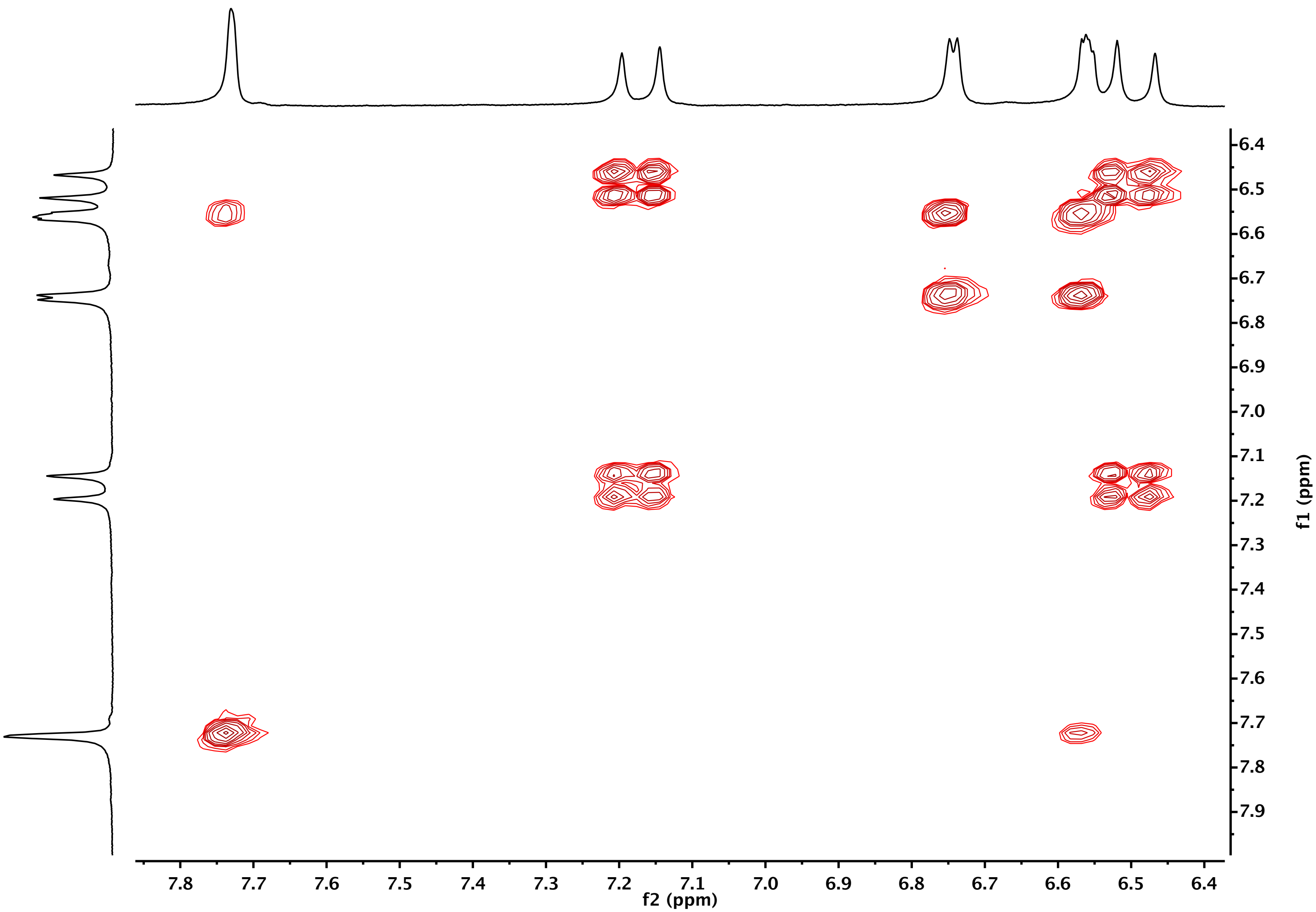

Figure S29. 500 MHz COSY NMR spectrum of FuranCurc-Mg.

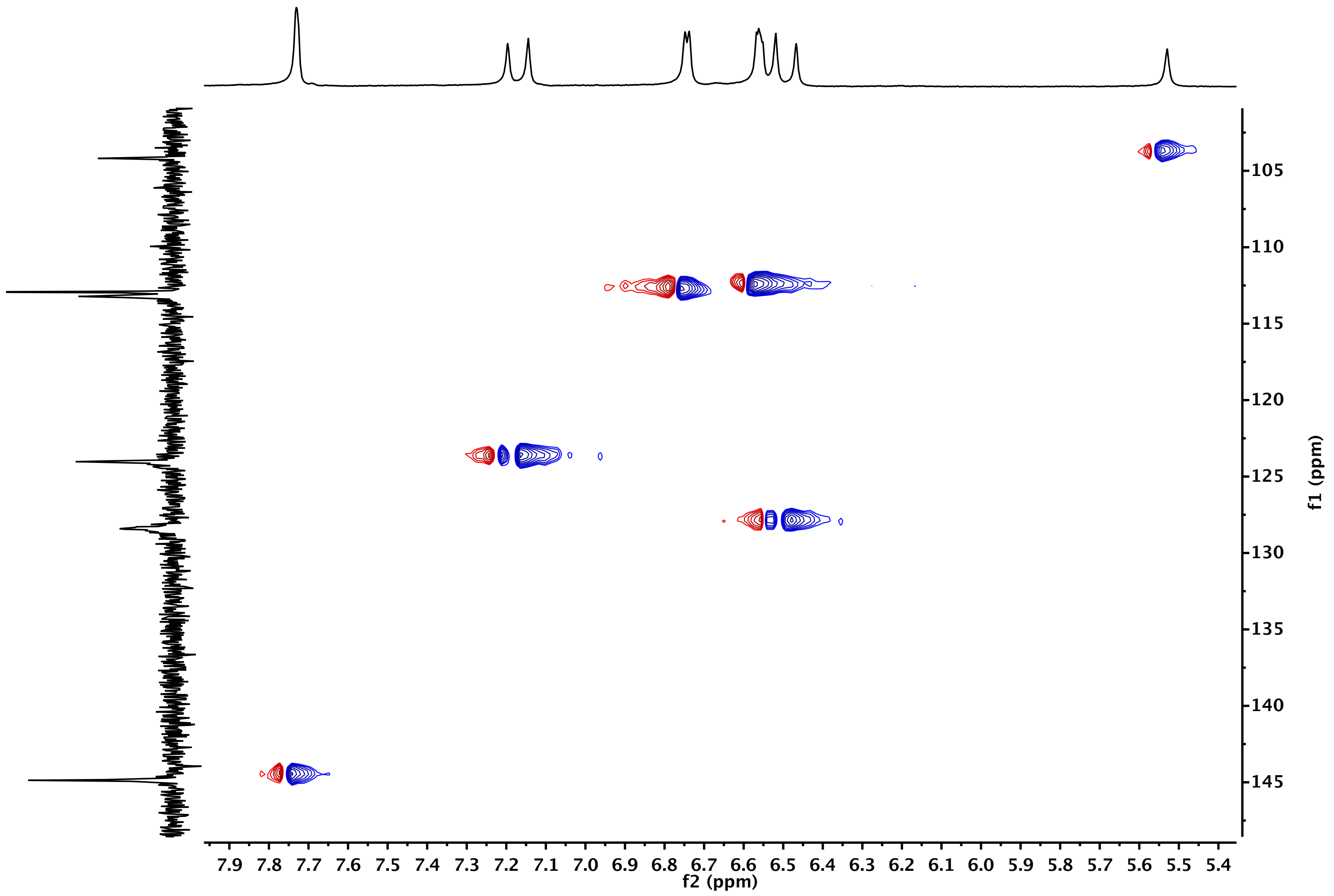

Figure S30. 500 MHz HSQC NMR spectrum of FuranCurc-Mg.

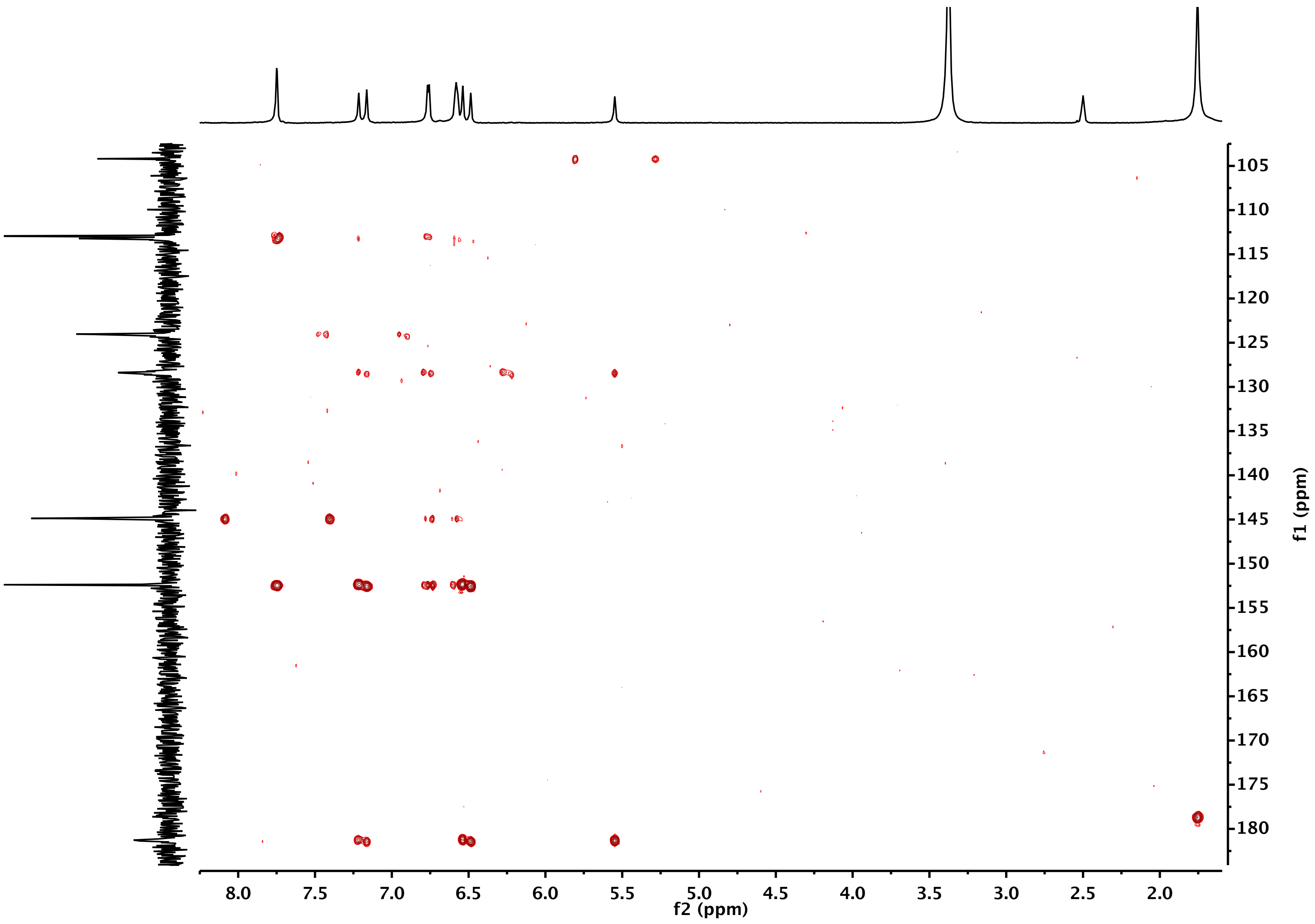

Figure S31. 500 MHz HMBC NMR spectrum of FuranCurc-Mg.

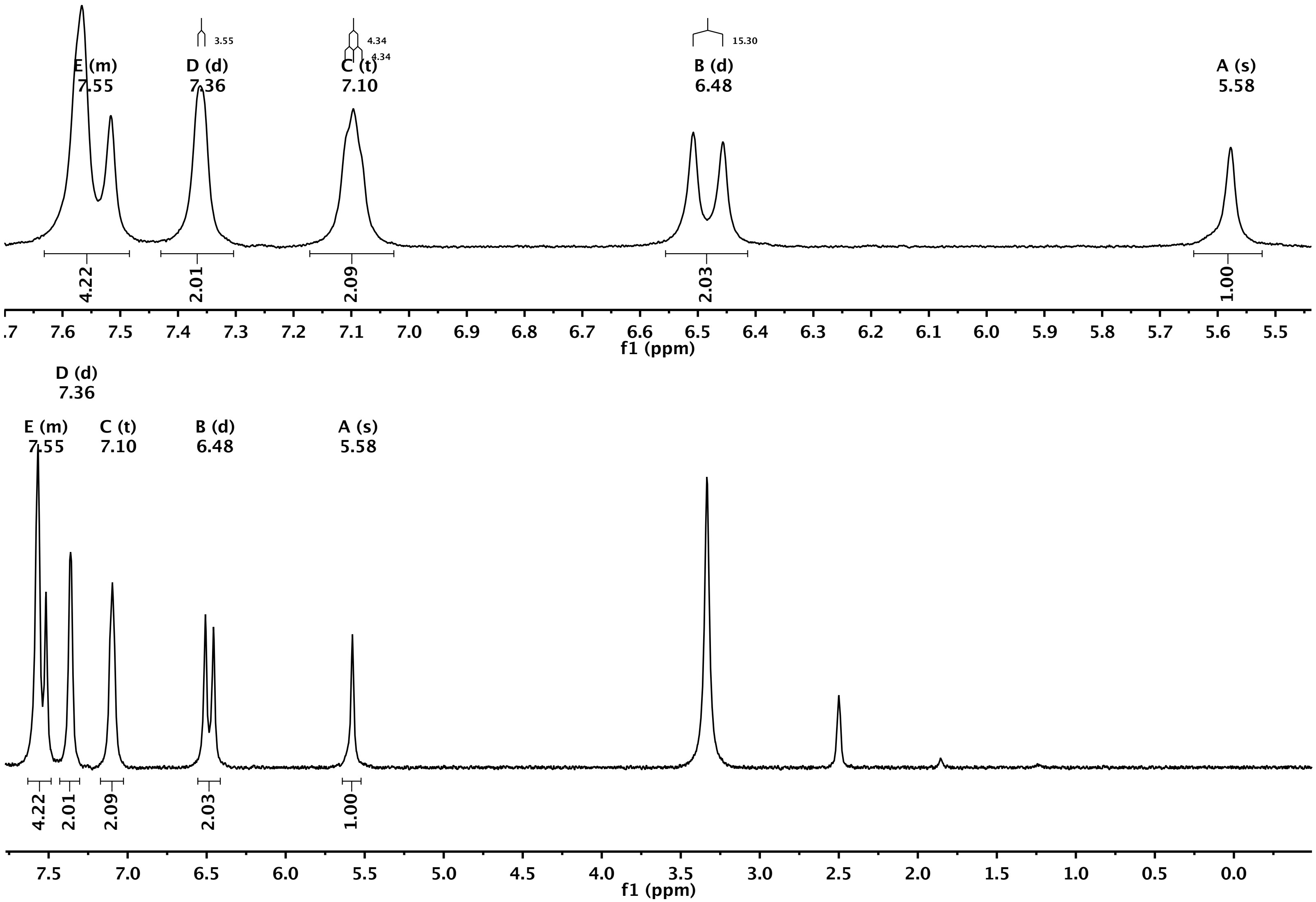

Figure S32. 500 MHz ^{1}H NMR spectrum of ThiopheneCurc-Mg.

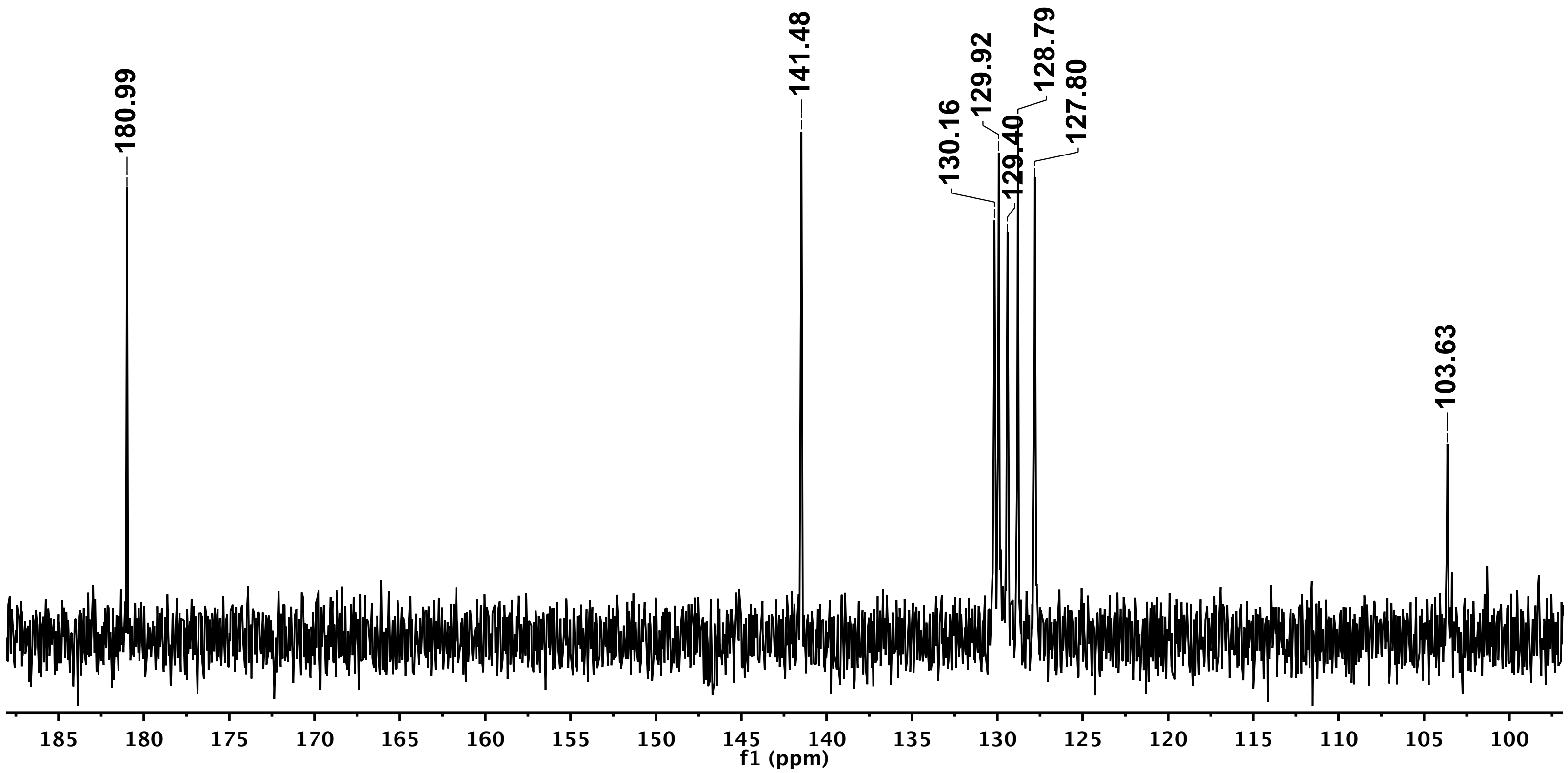

Figure S33. 125 MHz ^{13}C NMR spectrum of ThiopheneCurc-Mg.

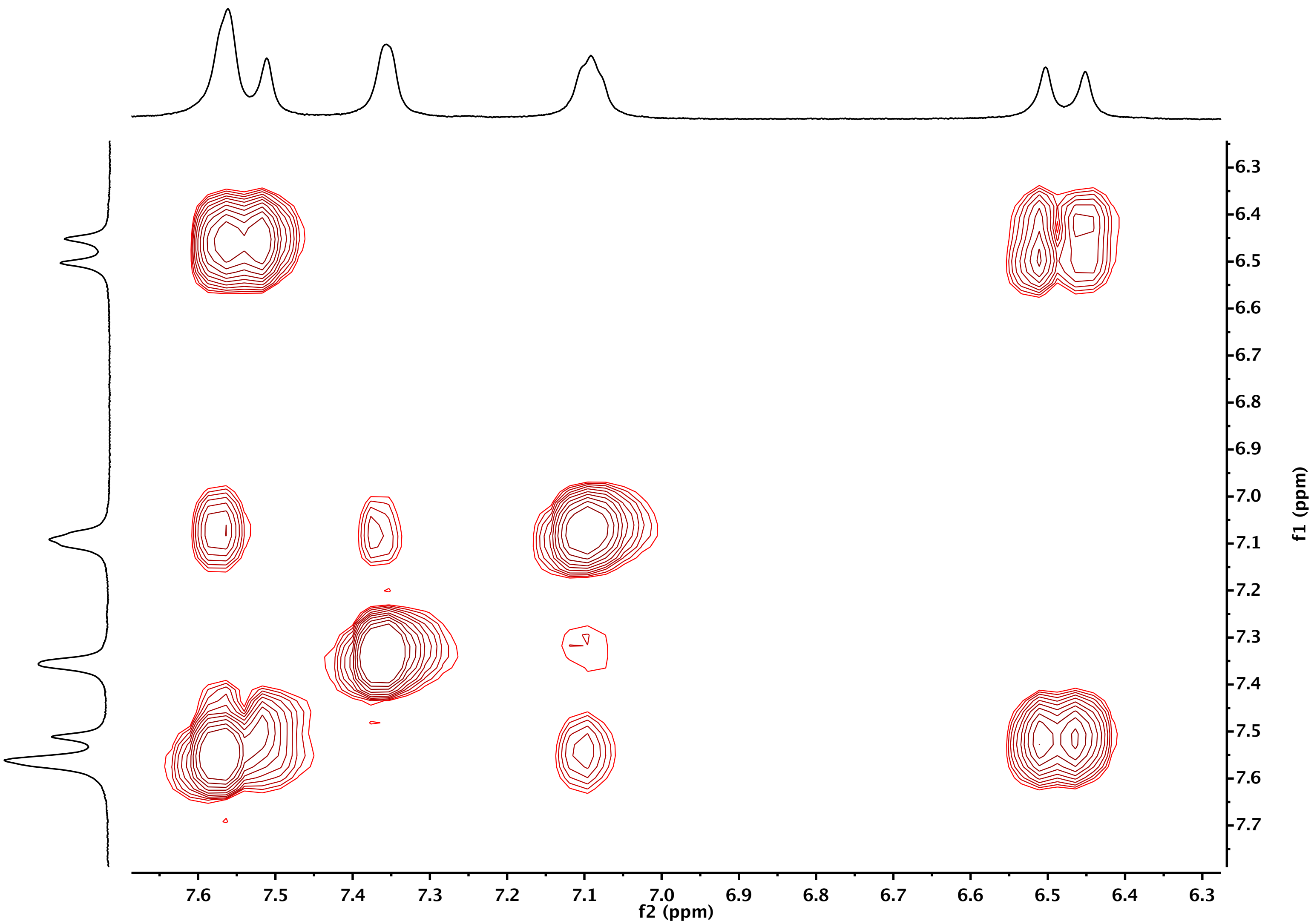

Figure S34. 500 MHz HSQC NMR spectrum of ThiopheneCurc-Mg.

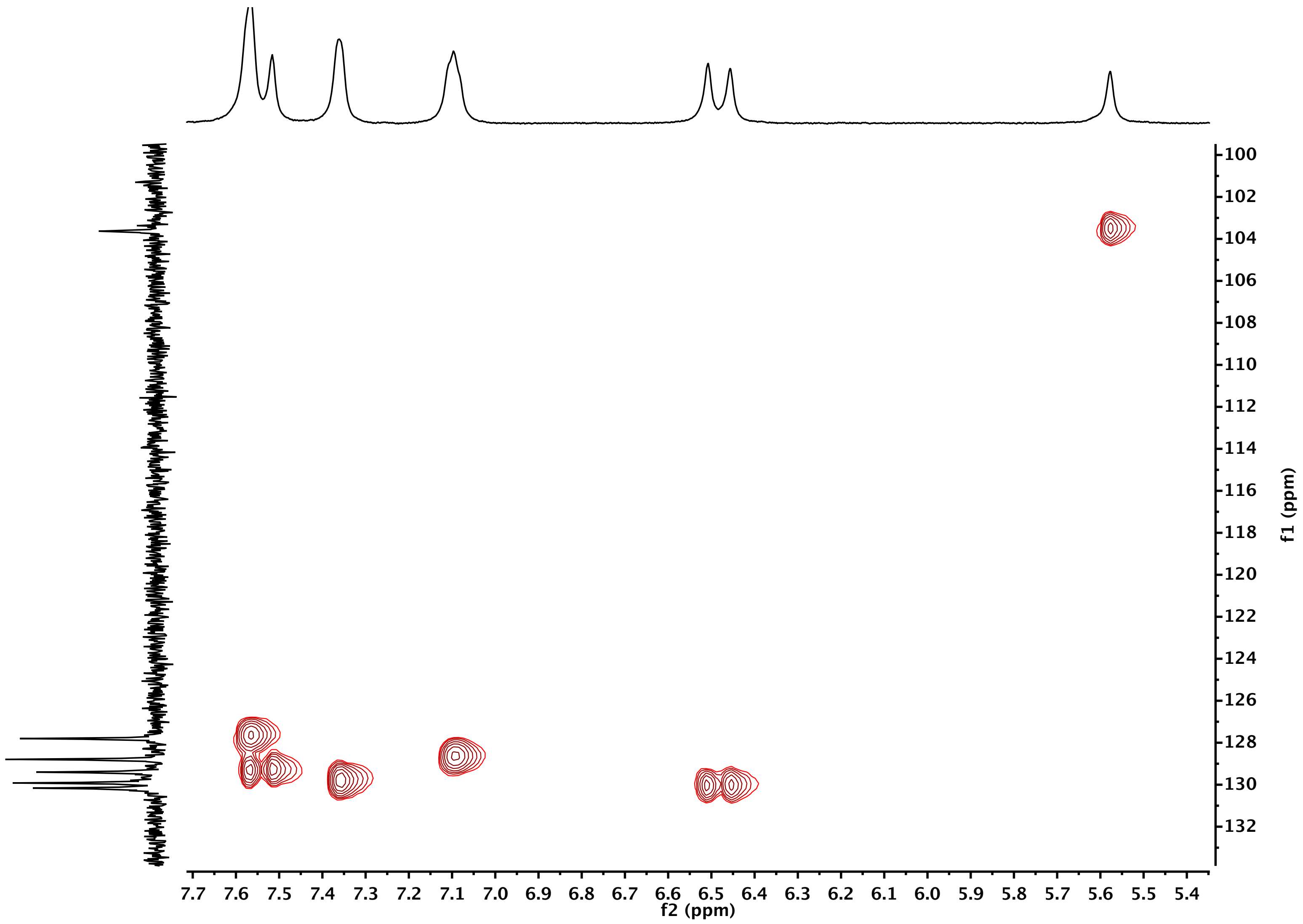

Figure S35. 500 MHz HSQC NMR spectrum of ThiopheneCurc-Mg.

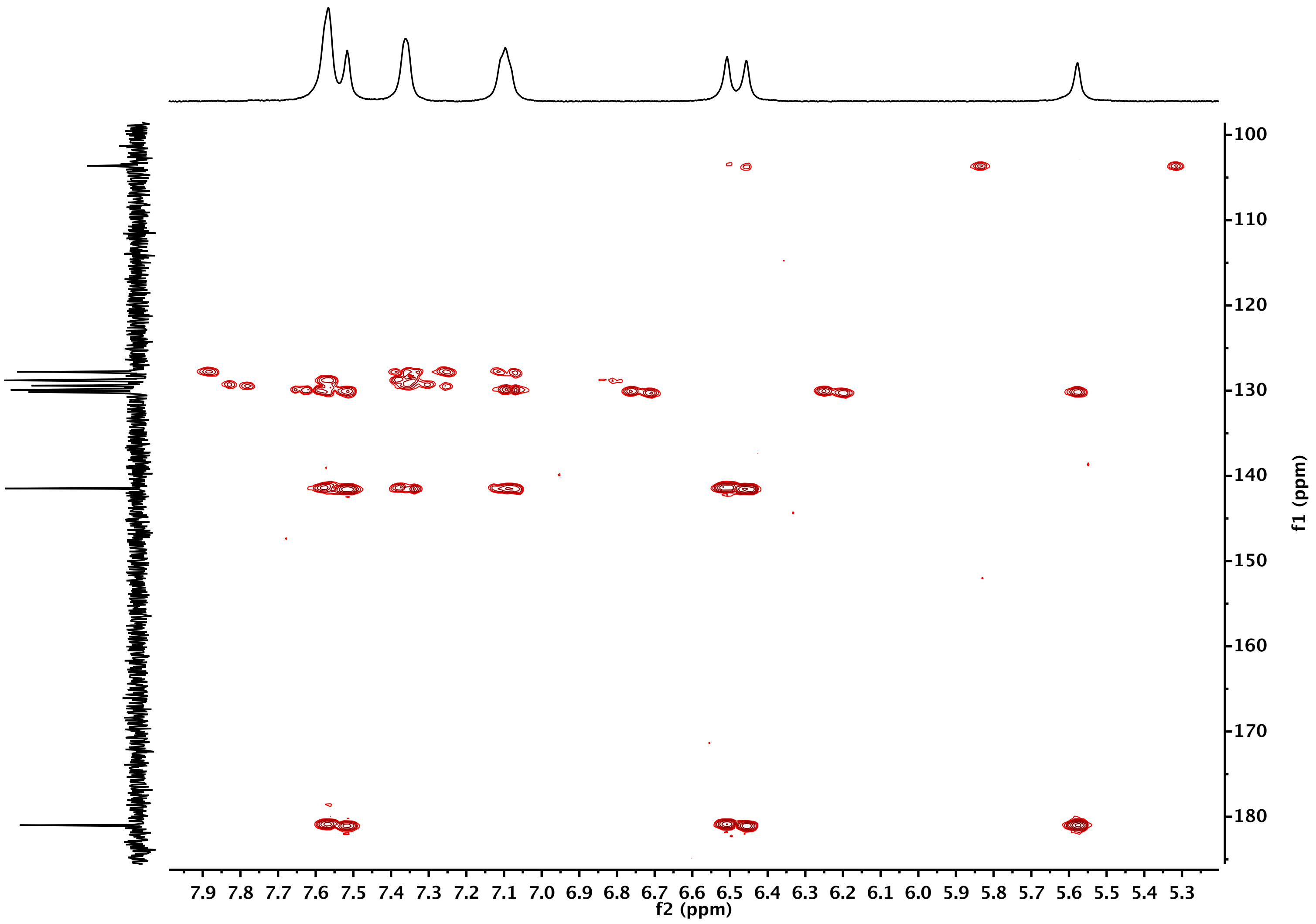

Figure S36. 500 MHz HMBC NMR spectrum of ThiopheneCurc-Mg.

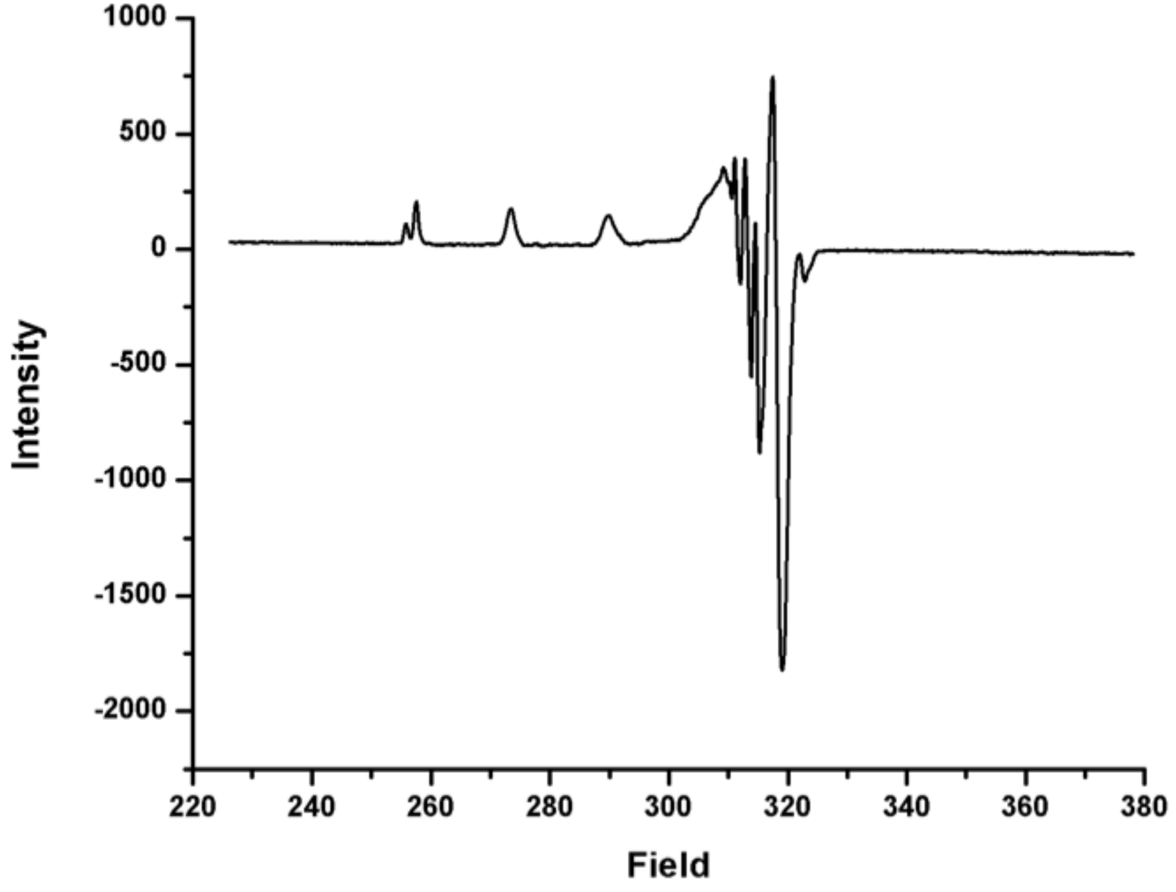

Figure S37. EPR Spectrum of N-methyl-pyrCurc-Cu.

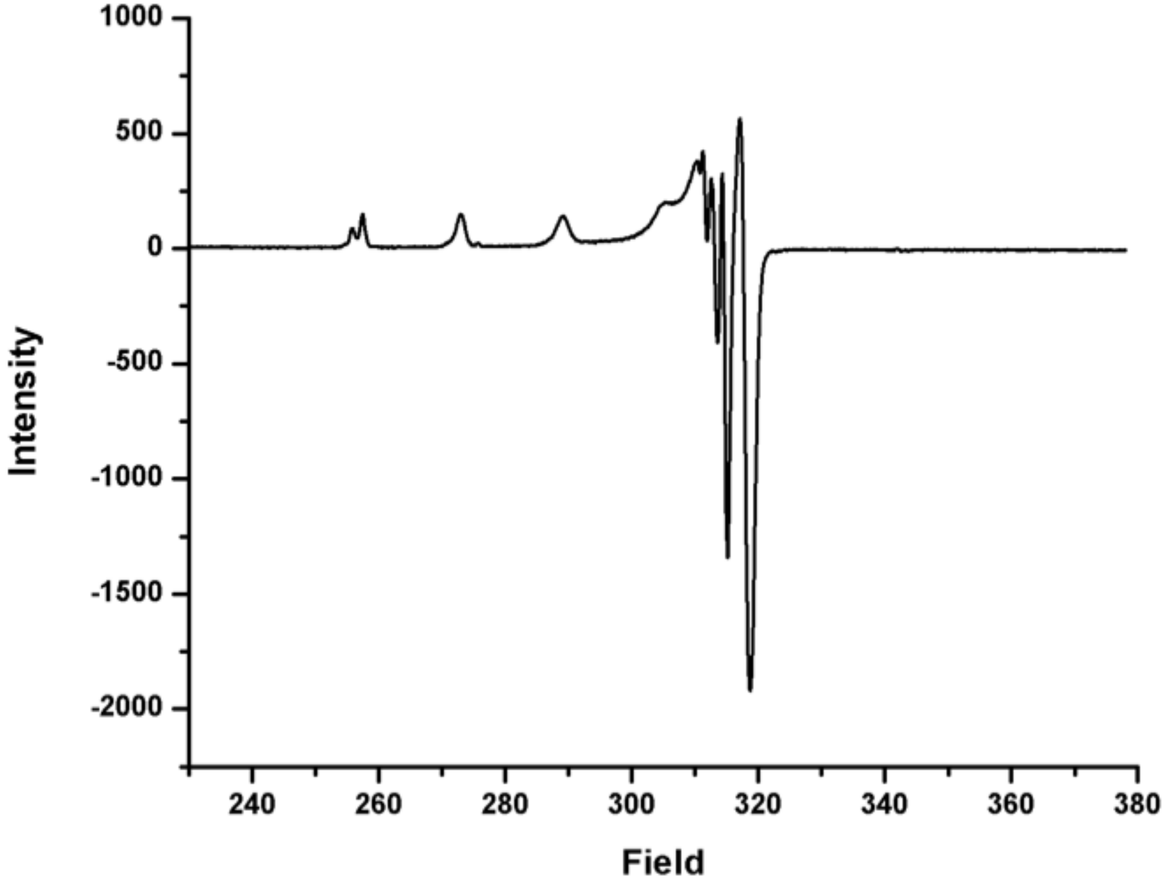

Figure S38. EPR Spectrum of FuranCurc-Cu.

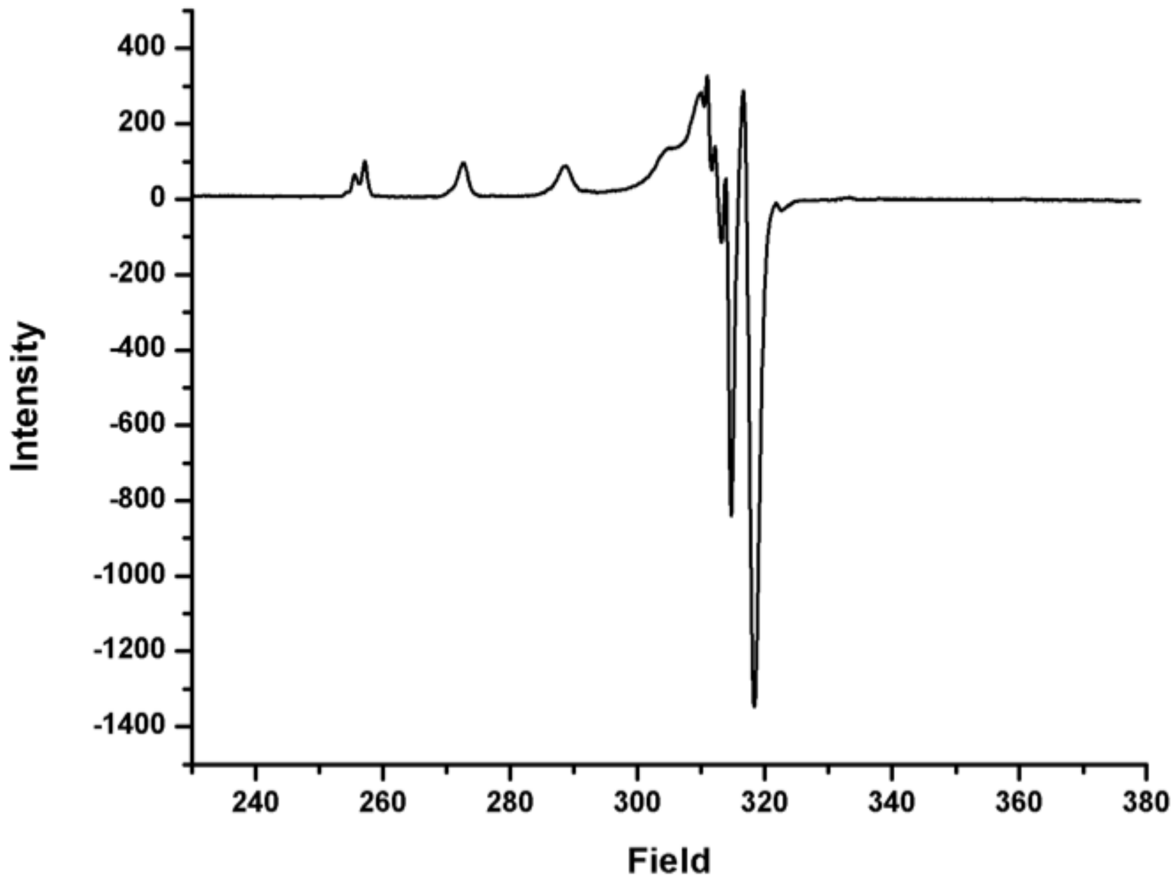

Figure S39. EPR Spectrum of ThiopheneCurc-Cu.

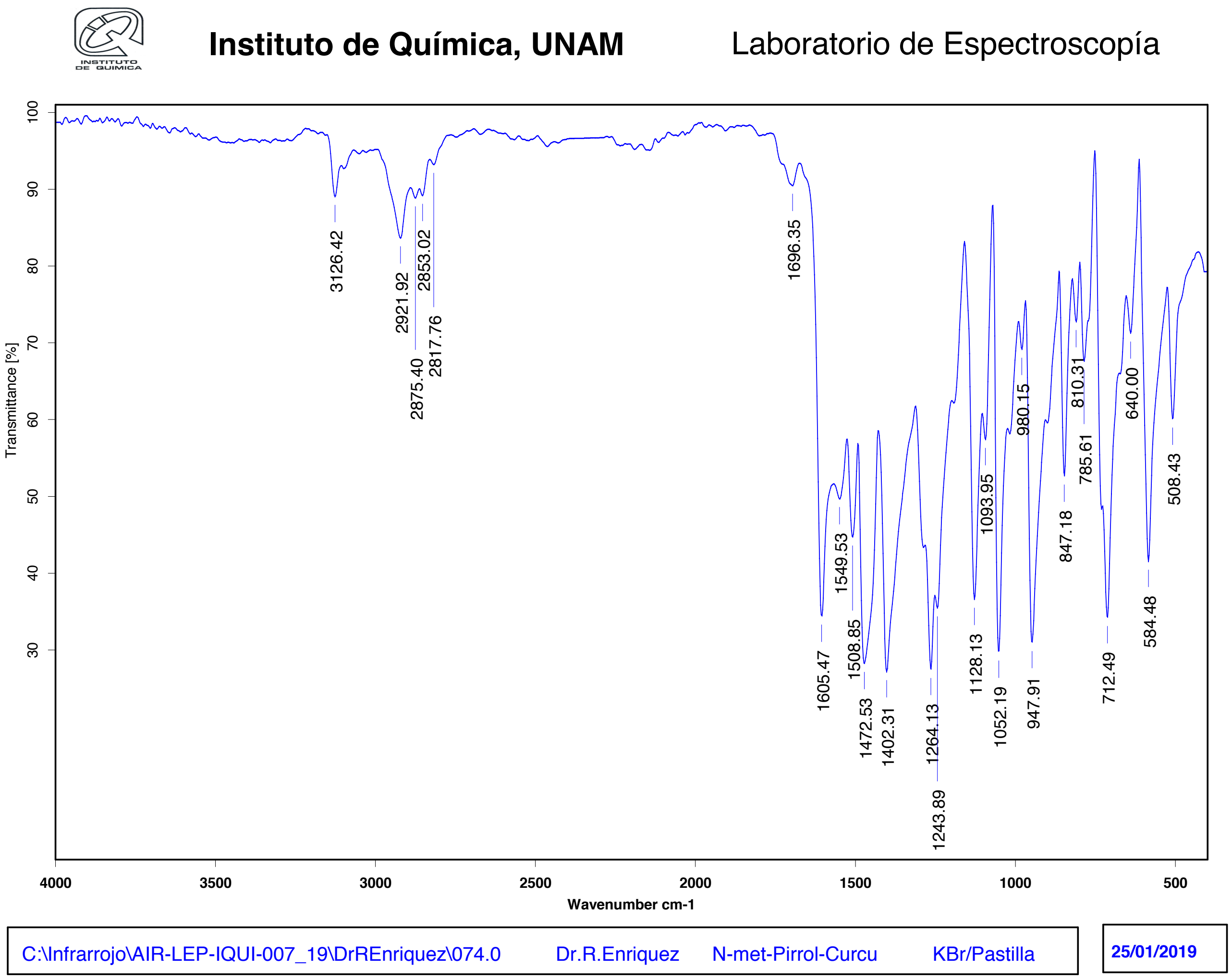

Figure S40. IR Spectrum of N-methyl-pyrCurc.

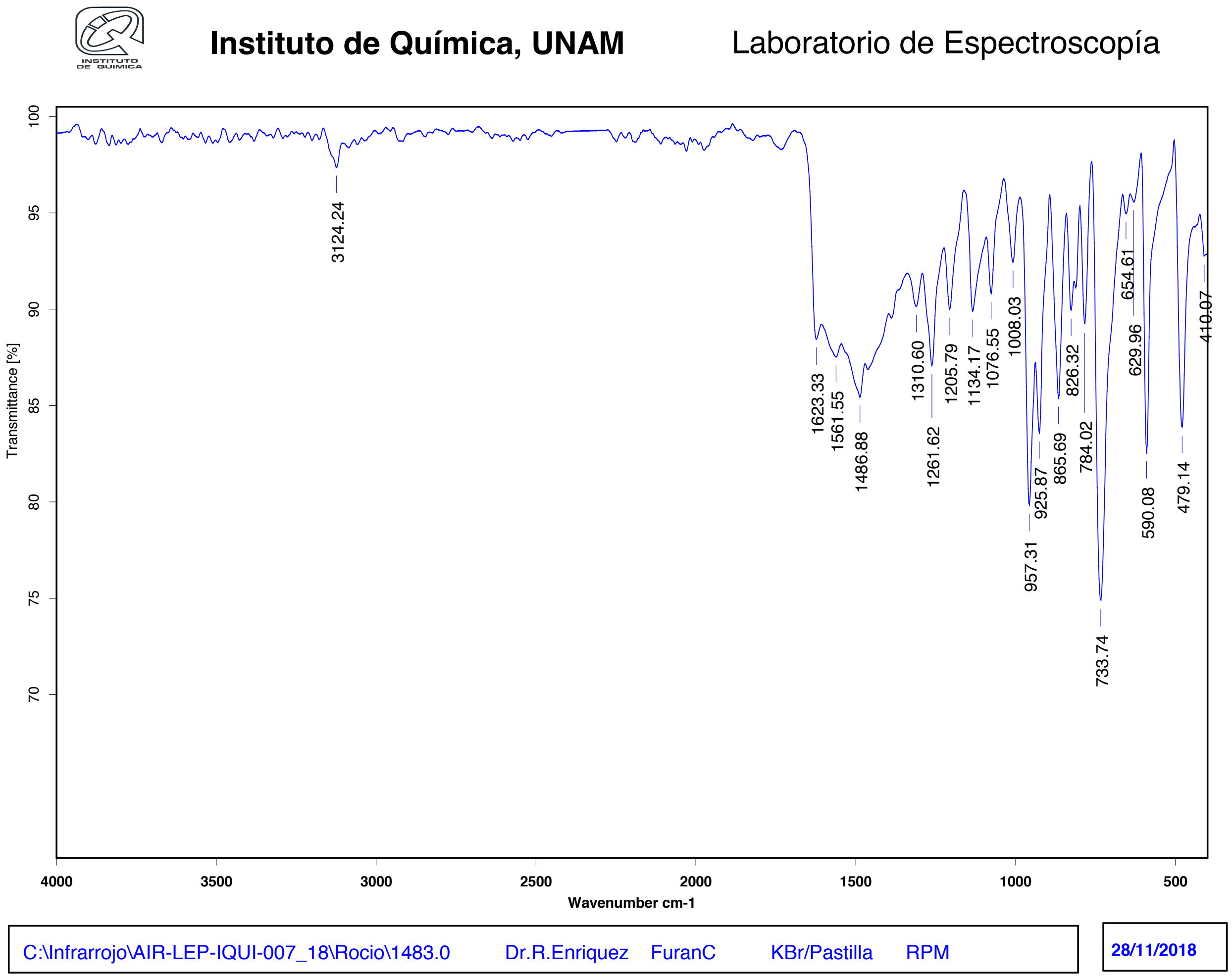

Figure S41. IR Spectrum of FuranCurc.

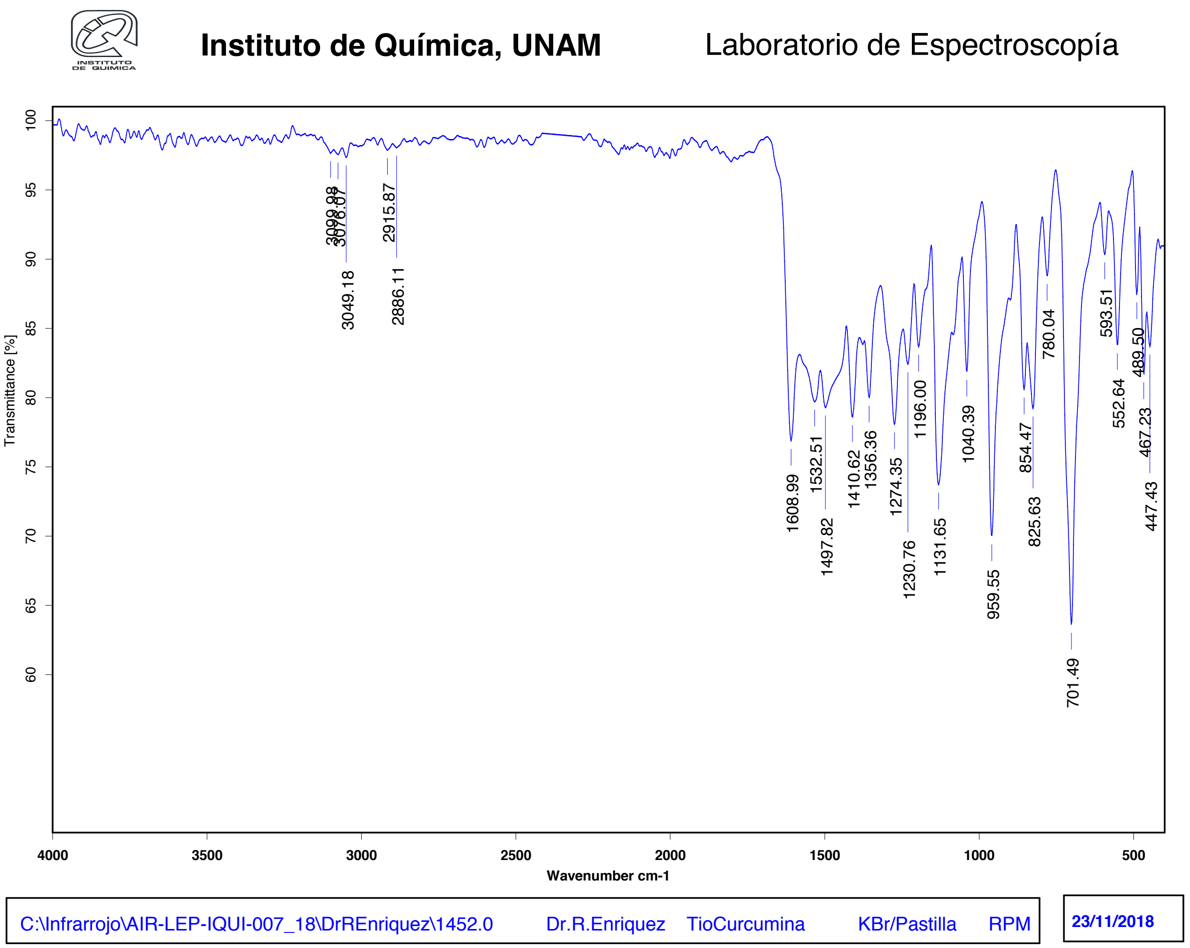

Figure S42. IR Spectrum of ThiopheneCurc.

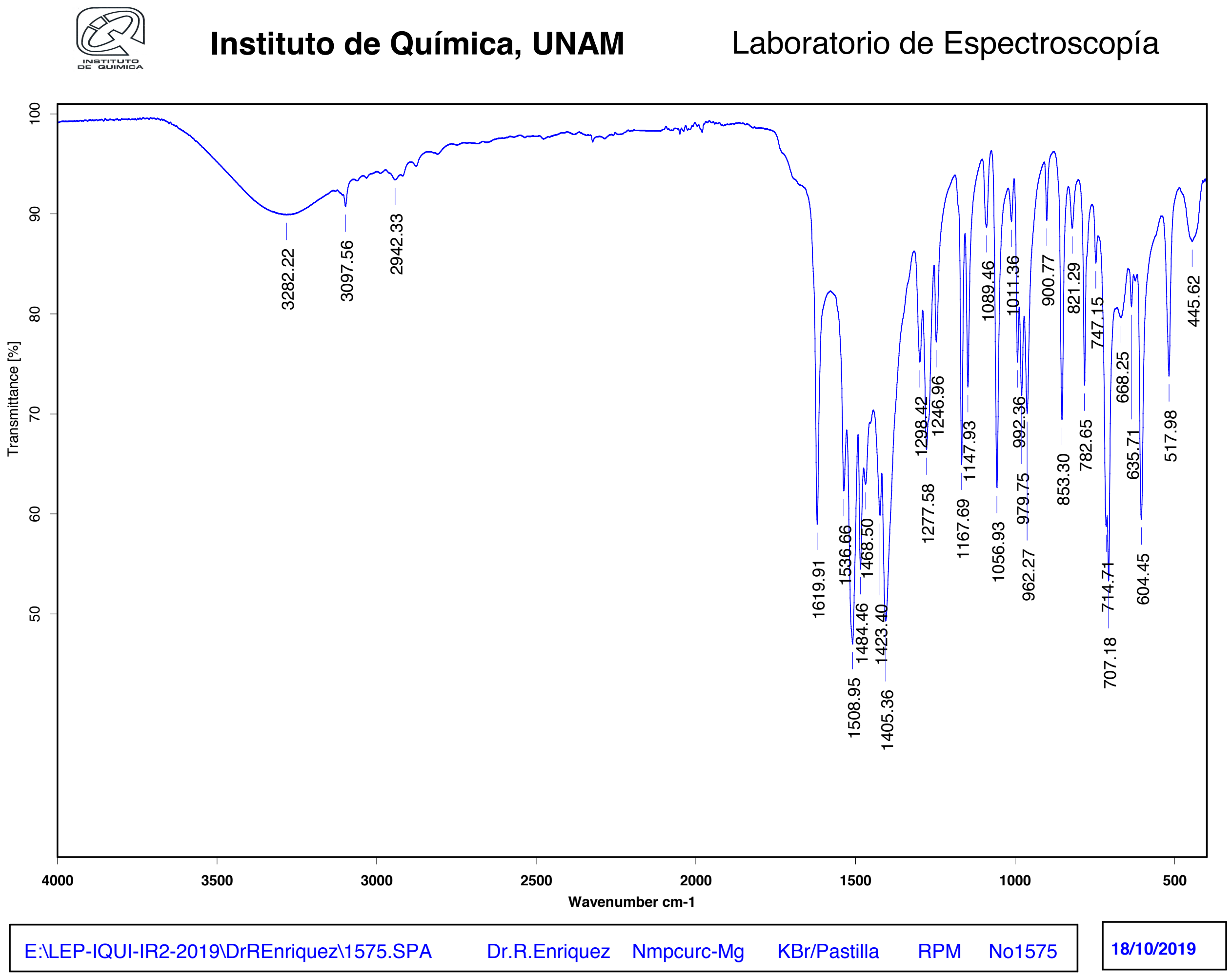

Figure S43. IR Spectrum of N-methyl-pyrCurc-Mg.

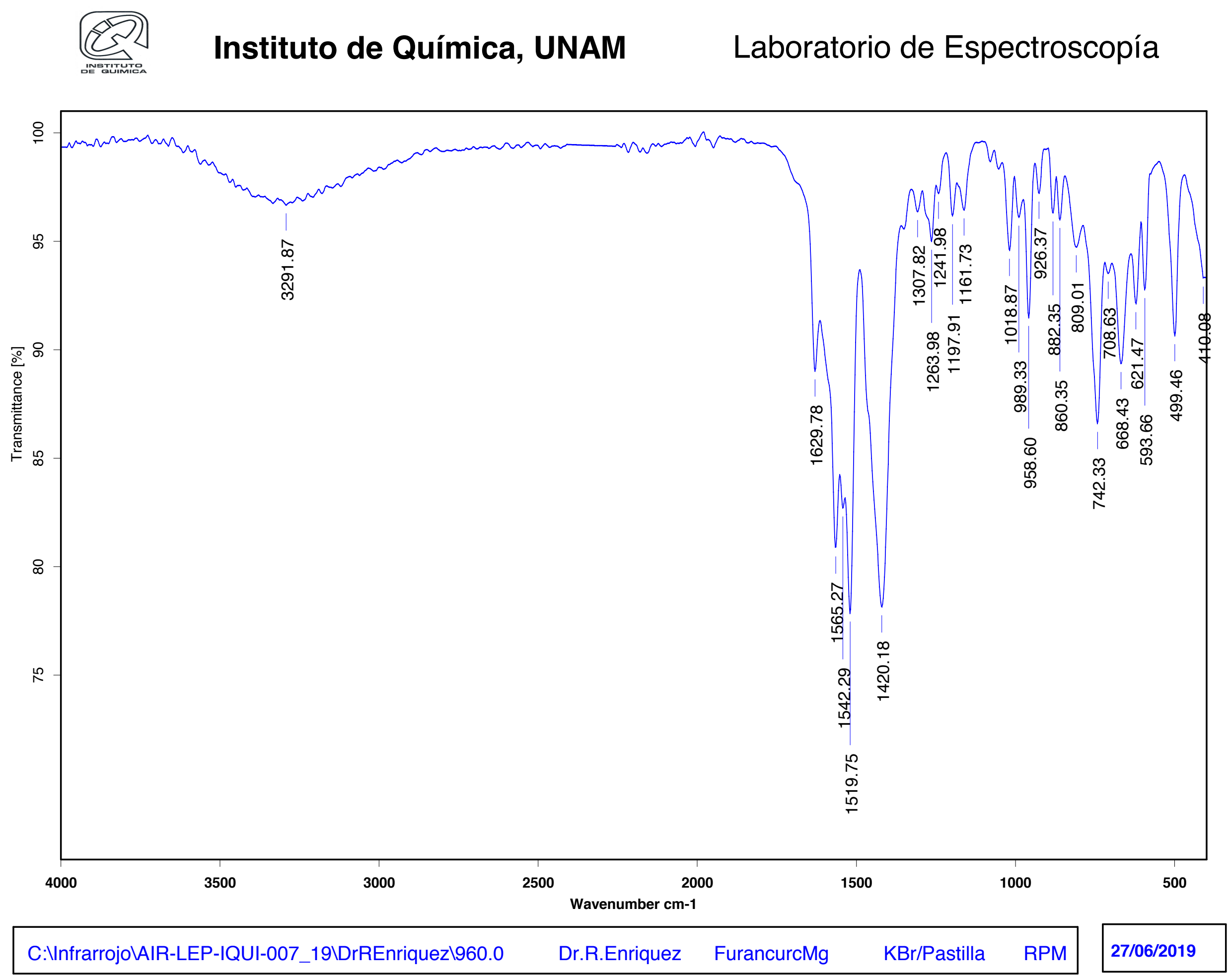

Figure S44. IR Spectrum of FuranCurc-Mg.

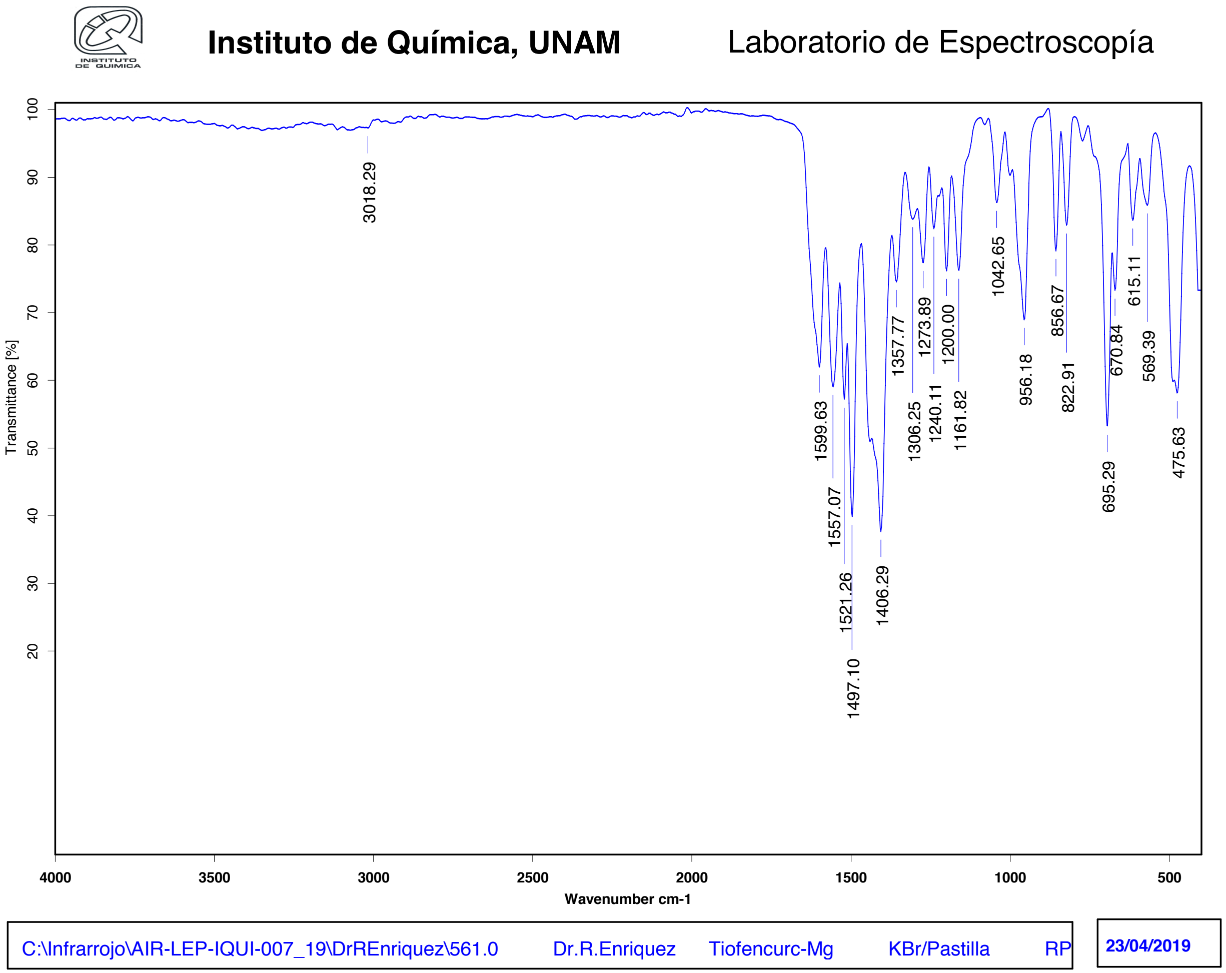

Figure S45. IR Spectrum of ThiopheneCurc -Mg.

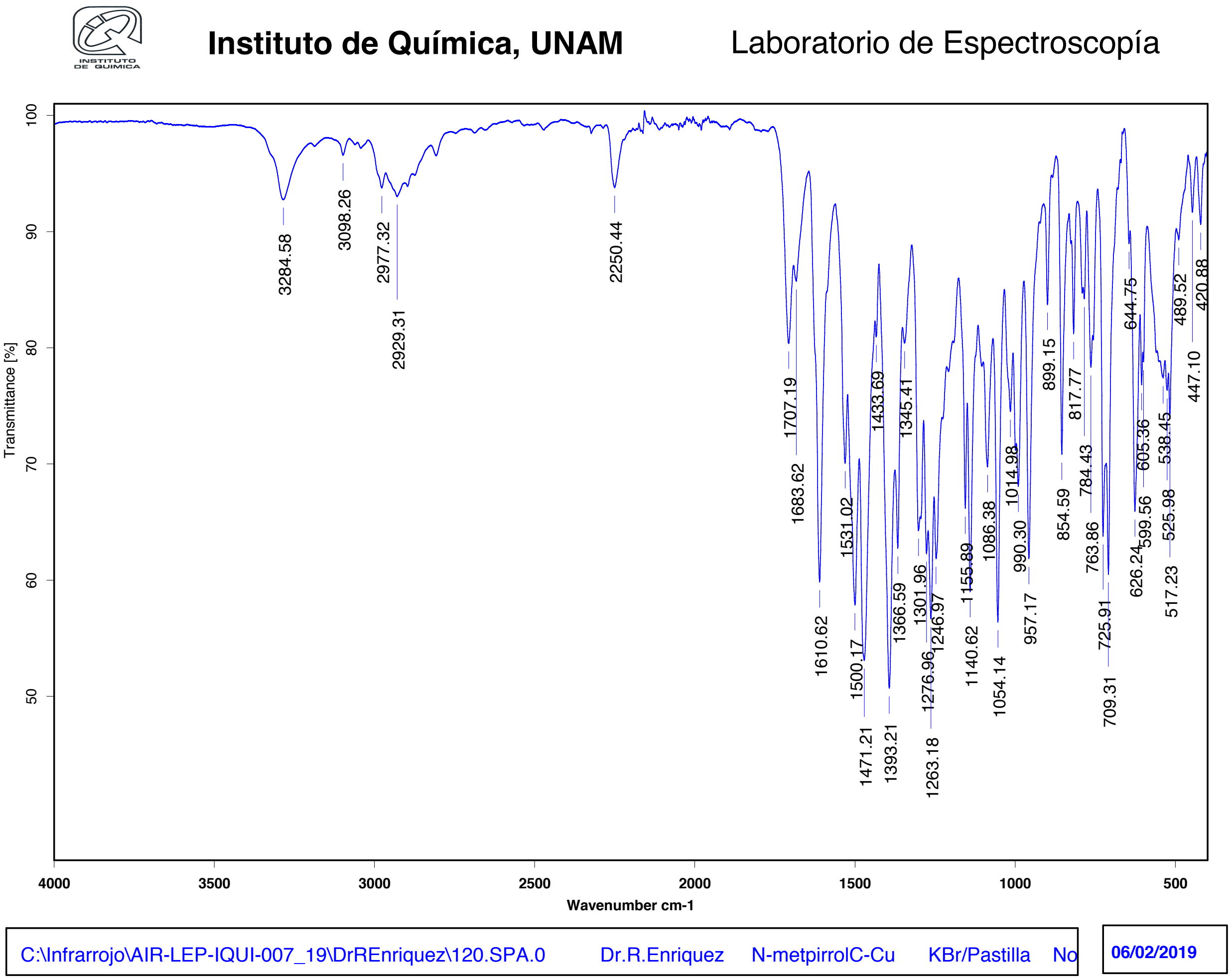

Figure S46. IR Spectrum of N-methyl-pyrCurc-Cu.

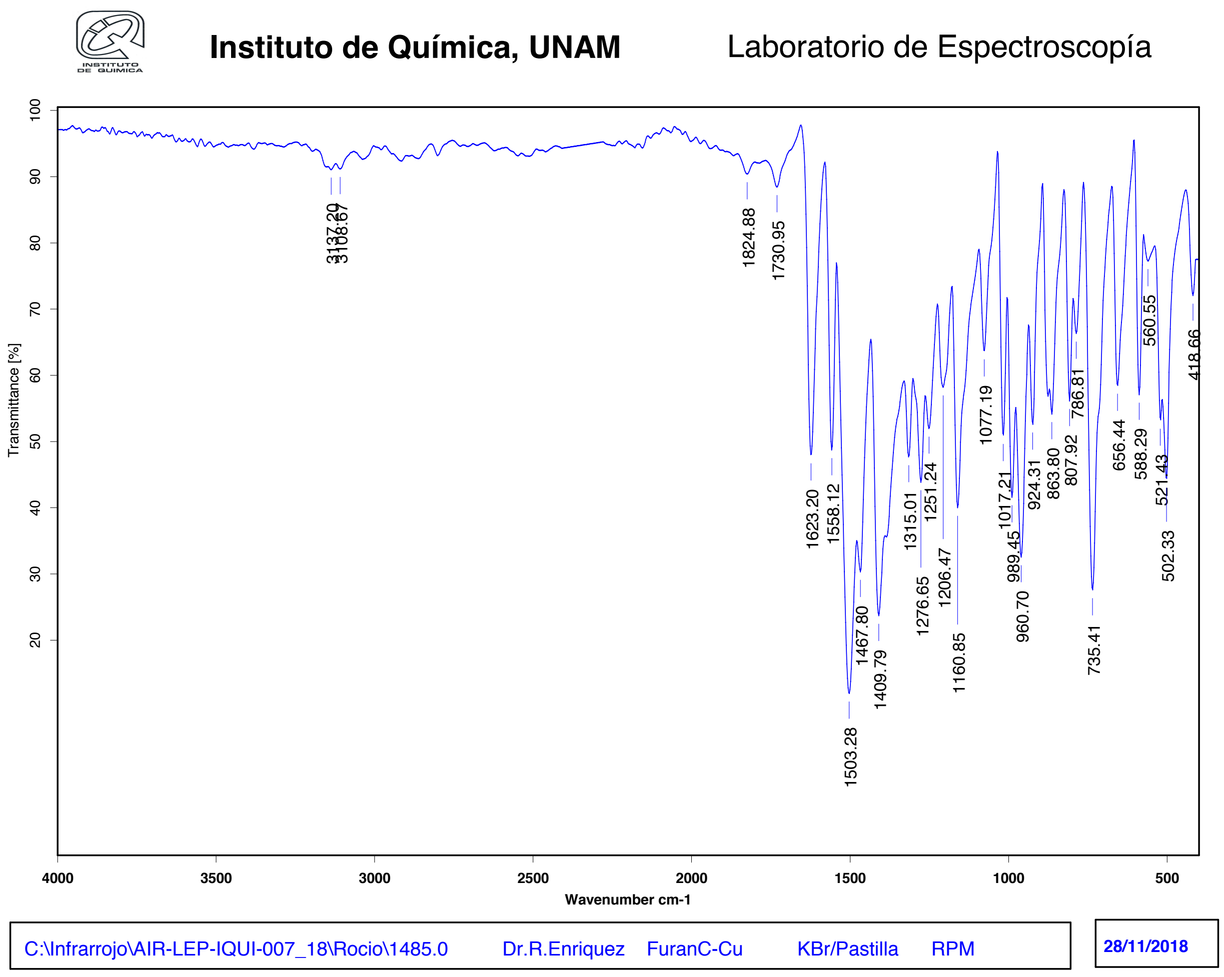

Figure S47. IR Spectrum of FuranCurc-Cu.

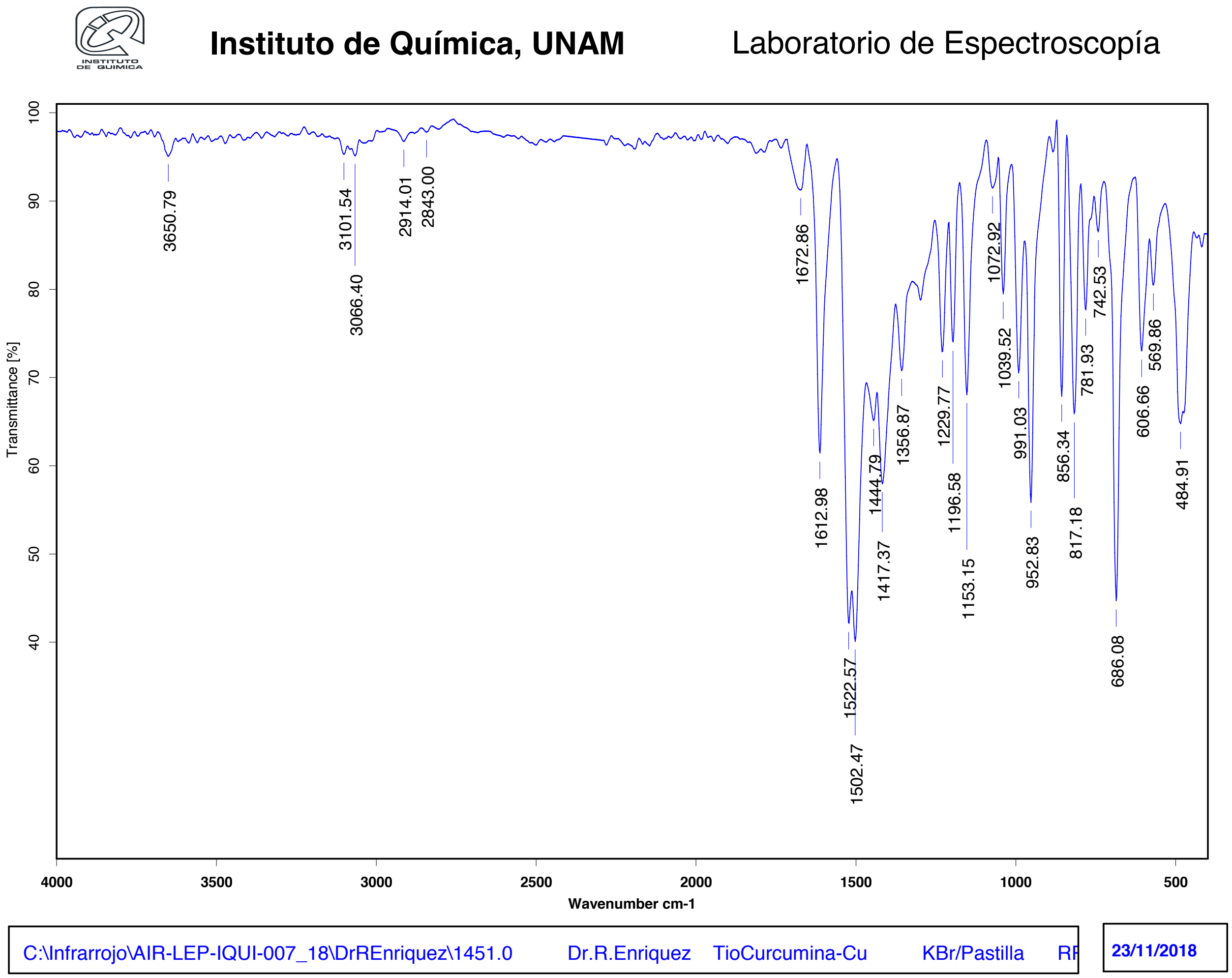

Figure S48. IR Spectrum of ThiopheneCurc-Cu.

Display Report

Analysis Info

Analysis Name	D:\Data\Daniel\030221_Nmpcurc.d	Acquisition Date	02/03/2021 12:39:24 p.m.
Method	Tune pos low.m	Operator	Daniel Arrieta
Sample Name	030221_Nmpcurc	Instrument	micrOTOF-Q 228888.10392
Comment			

Acquisition Parameter

Source Type	ESI	Ion Polarity	Positive	Set Nebulizer	0.4 Bar
Focus	Active	Set Capillary	4500 V	Set Dry Heater	180 °C
Scan Begin	50 m/z	Set End Plate Offset	-500 V	Set Dry Gas	4.0 l/min
Scan End	3000 m/z	Set Collision Cell RF	150.0 Vpp	Set Divert Valve	Source

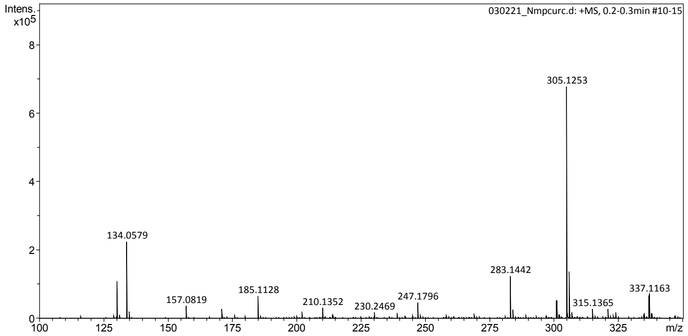

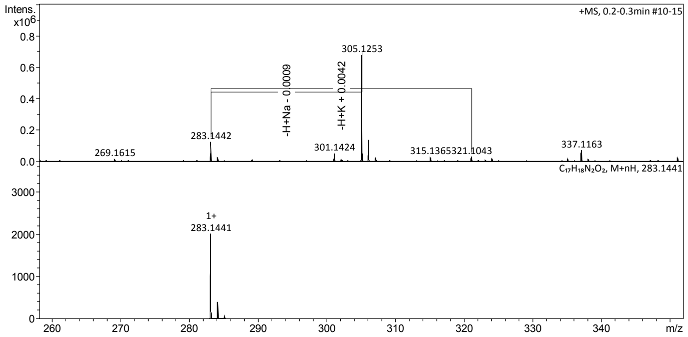

Figura S49. Mass Spectrum of N-methyl-pyrCurc.

Display Report

Analysis Info

Analysis Name	D:\Data\Daniel\030221_Furancurc.d	Acquisition Date	02/03/2021 12:56:21 p.m.
Method	Tune pos low.m	Operator	Daniel Arrieta
Sample Name	030221_Furancurc	Instrument	micrOTOF-Q 228888.10392
Comment			

Acquisition Parameter

Source Type	ESI	Ion Polarity	Positive	Set Nebulizer	0.4 Bar
Focus	Active	Set Capillary	4500 V	Set Dry Heater	180 °C
Scan Begin	50 m/z	Set End Plate Offset	-500 V	Set Dry Gas	4.0 l/min
Scan End	3000 m/z	Set Collision Cell RF	150.0 Vpp	Set Divert Valve	Source

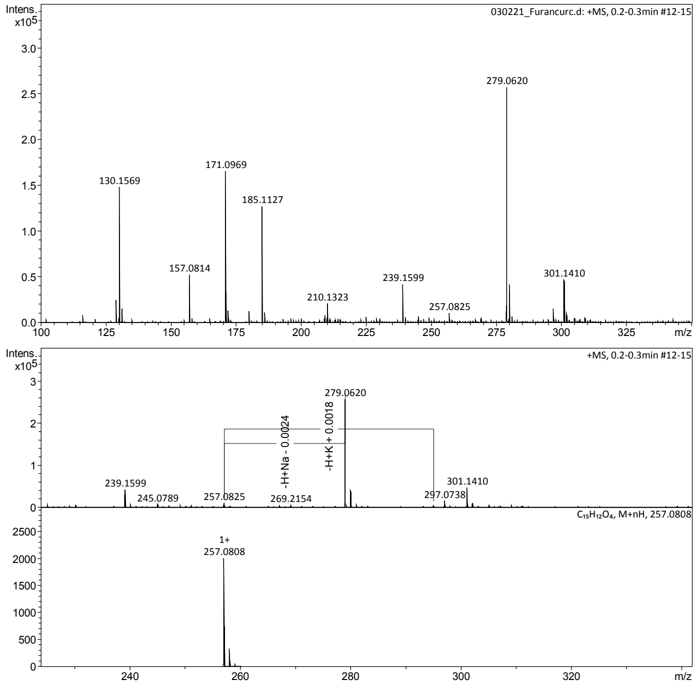

Bruker Compass DataAnalysis 4.1 printed: 04/03/2021 05:35:49 p.m. by: Daniel Arrieta Page 1 of 1

Figura S50. Mass Spectrum of mass exact of FuranCurc.

Display Report

Analysis Info

Analysis Name	D:\Data\Daniel\030221_Tiofencurc.d	Acquisition Date	02/03/2021 12:31:57 p.m.
Method	Tune pos low.m	Operator	Daniel Arrieta
Sample Name	030221_Tiofencurc	Instrument	micrOTOF-Q 228888.10392
Comment			

Acquisition Parameter

Source Type	ESI	Ion Polarity	Positive	Set Nebulizer	0.4 Bar
Focus	Active	Set Capillary	4500 V	Set Dry Heater	180 °C
Scan Begin	50 m/z	Set End Plate Offset	-500 V	Set Dry Gas	4.0 l/min
Scan End	3000 m/z	Set Collision Cell RF	150.0 Vpp	Set Divert Valve	Source

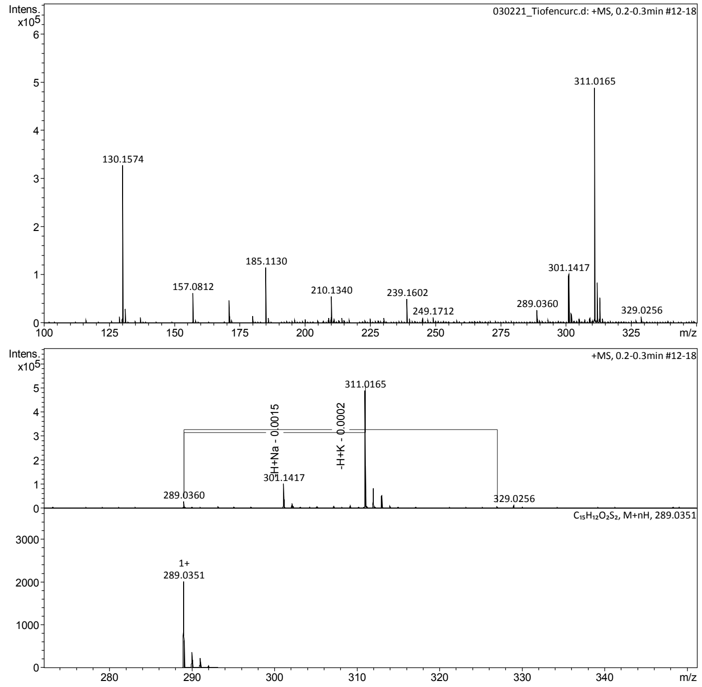

Bruker Compass DataAnalysis 4.1 printed: 04/03/2021 05:39:32 p.m. by: Daniel Arrieta Page 1 of 1

Figura S51. Mass Spectrum of ThiopheneCurc.

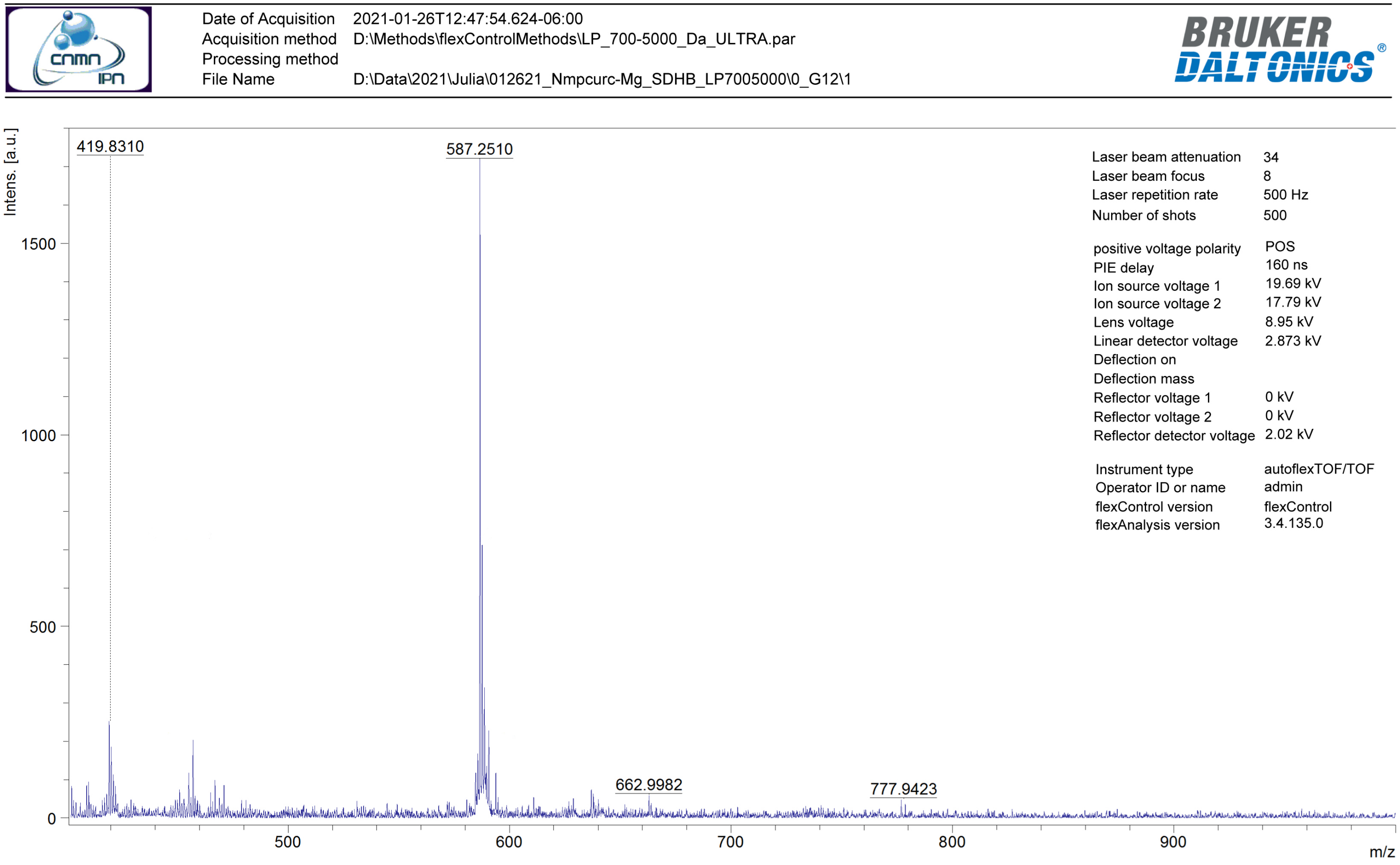

Figura S52. Mass Spectrum of N-methyl-pyrCurc-Mg.

Date of Acquisition	2021-01-26T13:00:25.257-06:00
Acquisition method	D:\Methods\flexControlMethods\LP_700-5000_Da_ULTRA.par
Processing method	
File Name	D:\Data\2021\Julia\012621_Furancurc-Cu_SDHB_LP7005000_01\0_G20\1

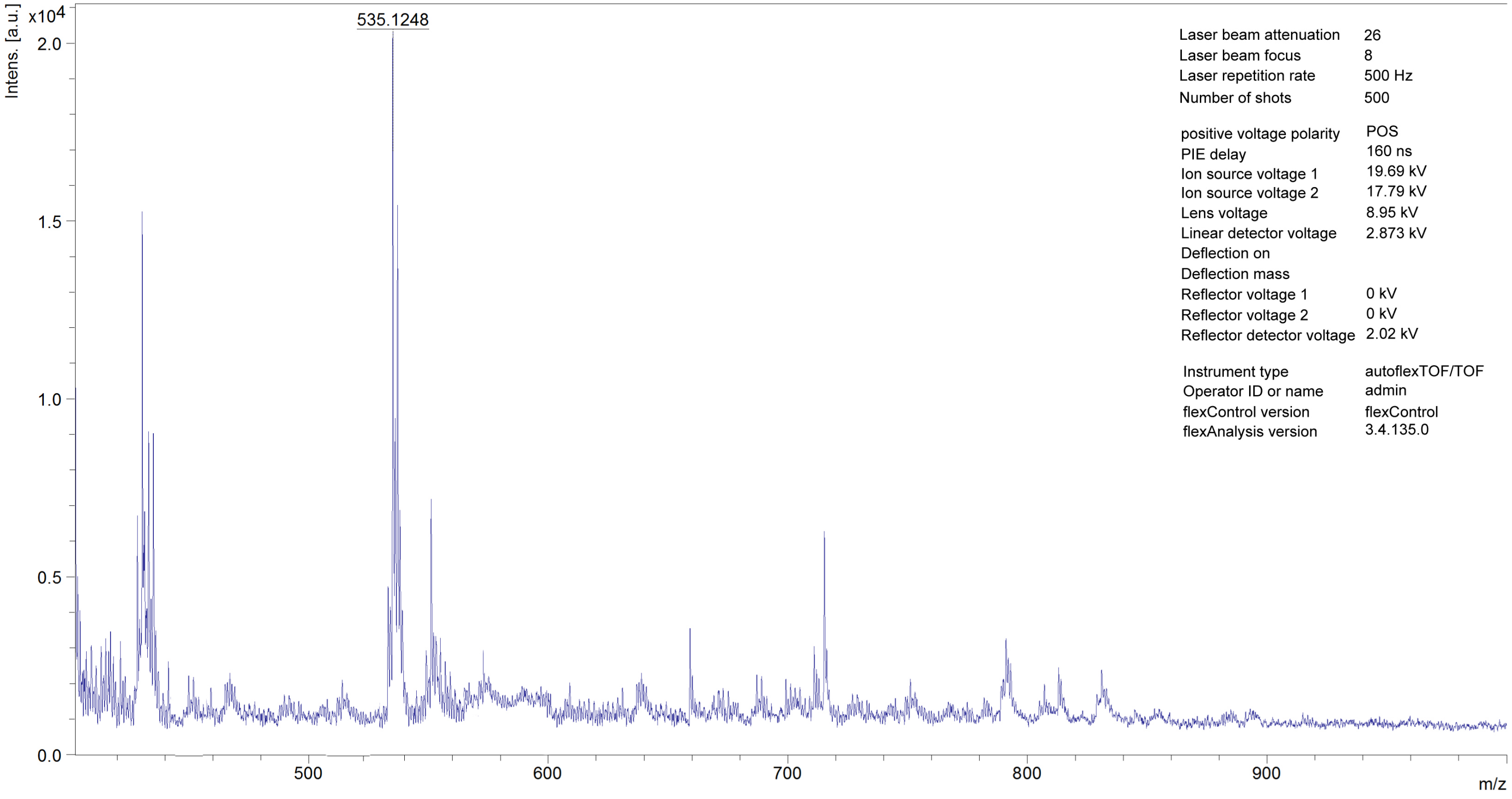

Figura S53. Mass Spectrum of mass exact of FuranCurc-Mg.

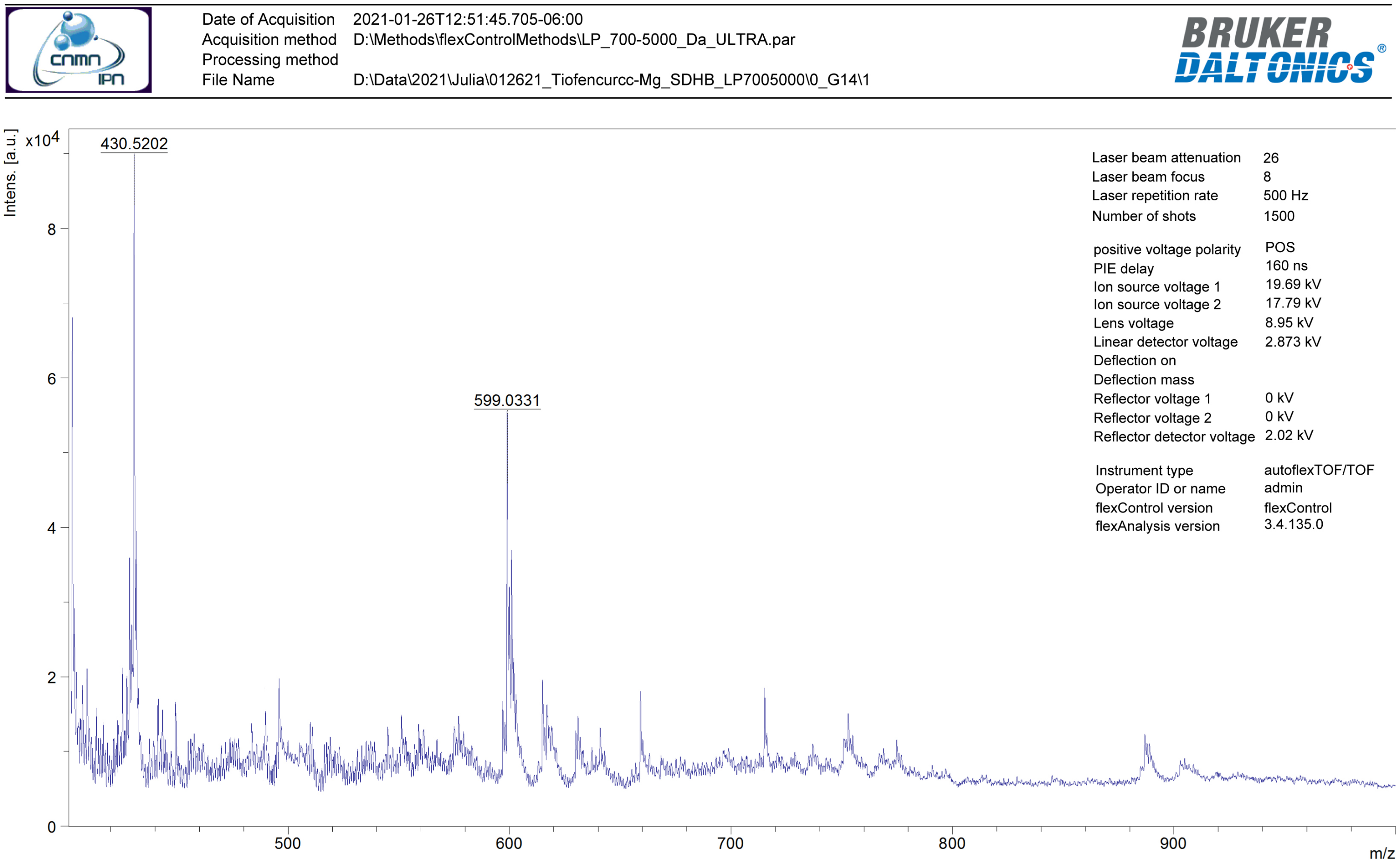

Figura S54. Mass Spectrum of mass exact of ThiopheneCurc-Mg.

Display Report

Analysis Info

Analysis Name	D:\Data\Daniel\020921_metilpirrolcurc-Cu.d	Acquisition Date	09/02/2021 11:18:09 a.m.
Method	Tune pos low.m	Operator	Daniel Arrieta
Sample Name	020921_metilpirrolcurc-Cu	Instrument	micrOTOF-Q 228888.10392
Comment			

Acquisition Parameter

Source Type	ESI	Ion Polarity	Positive	Set Nebulizer	0.4 Bar
Focus	Active	Set Capillary	4500 V	Set Dry Heater	180 °C
Scan Begin	50 m/z	Set End Plate Offset	-500 V	Set Dry Gas	4.0 l/min
Scan End	3000 m/z	Set Collision Cell RF	150.0 Vpp	Set Divert Valve	Source

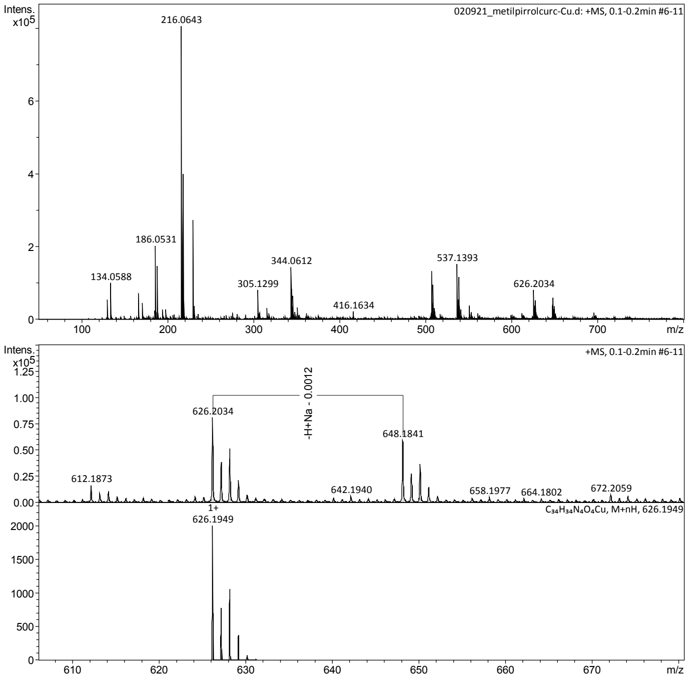

Bruker Compass DataAnalysis 4.1 printed: 10/02/2021 12:42:13 p.m. by: Daniel Arrieta Page 1 of 1

Figura S55. Mass Spectrum of N-methyl-pyrCurc-Cu.

Display Report

Analysis Info

Analysis Name	D:\Data\Daniel\020921_Furancur-Cu_Low.d	Acquisition Date	09/02/2021 10:49:20 a.m.
Method	Tune pos low.m	Operator	Daniel Arrieta
Sample Name	020921_Furancur-Cu_Low	Instrument	micrOTOF-Q 228888.10392
Comment			

Acquisition Parameter

Source Type	ESI	Ion Polarity	Positive	Set Nebulizer	0.4 Bar
Focus	Active	Set Capillary	4500 V	Set Dry Heater	180 °C
Scan Begin	50 m/z	Set End Plate Offset	-500 V	Set Dry Gas	4.0 l/min
Scan End	3000 m/z	Set Collision Cell RF	150.0 Vpp	Set Divert Valve	Source

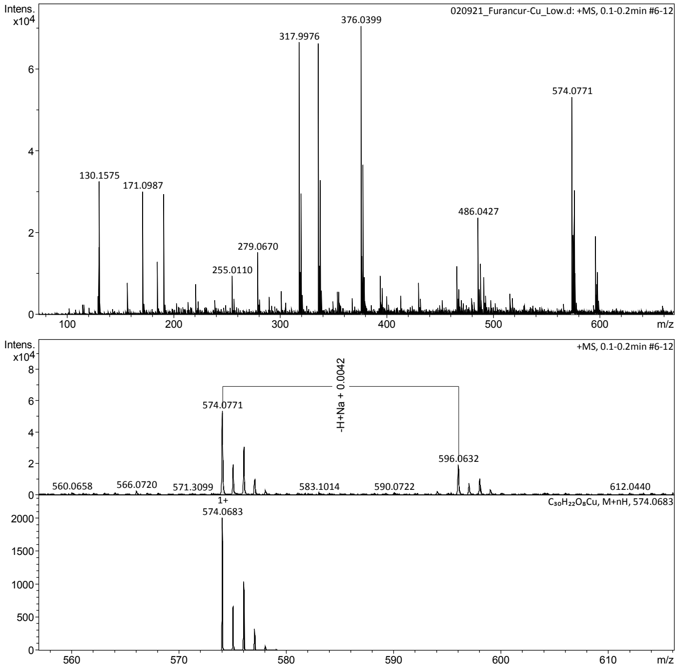

Figura S56. Mass Spectrum of FuranCurc-Cu.

Display Report

Analysis Info

Analysis Name	D:\Data\Daniel\020921_Tiofencurc-Cu-Low.d	Acquisition Date	09/02/2021 10:56:40 a.m.
Method	Tune pos low.m	Operator	Daniel Arrieta
Sample Name	020921_Tiofencurc-Cu-Low	Instrument	micrOTOF-Q 228888.10392
Comment			

Acquisition Parameter

Source Type	ESI	Ion Polarity	Positive	Set Nebulizer	0.4 Bar
Focus	Active	Set Capillary	4500 V	Set Dry Heater	180 °C
Scan Begin	50 m/z	Set End Plate Offset	-500 V	Set Dry Gas	4.0 l/min
Scan End	3000 m/z	Set Collision Cell RF	150.0 Vpp	Set Divert Valve	Source

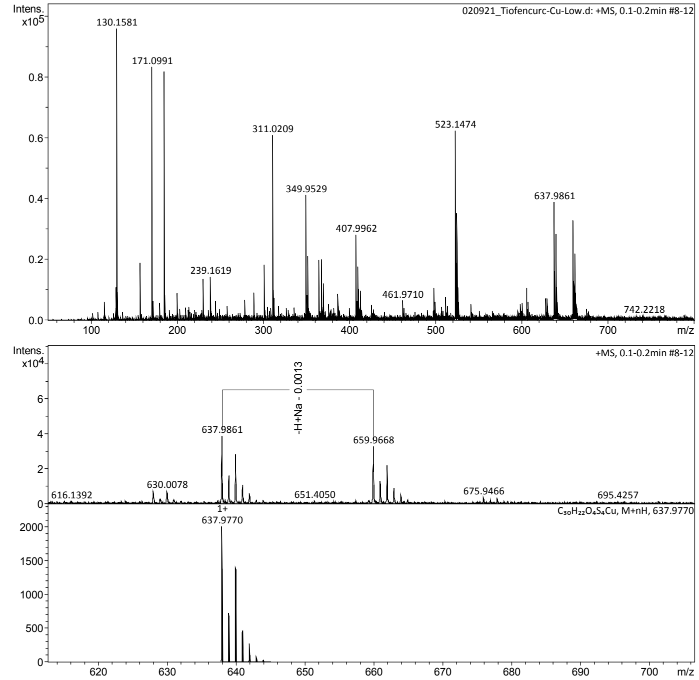

Bruker Compass DataAnalysis 4.1 printed: 10/02/2021 12:22:50 p.m. by: Daniel Arrieta Page 1 of 1

Figura S57. Mass Spectrum of ThiopheneCurc-Cu.

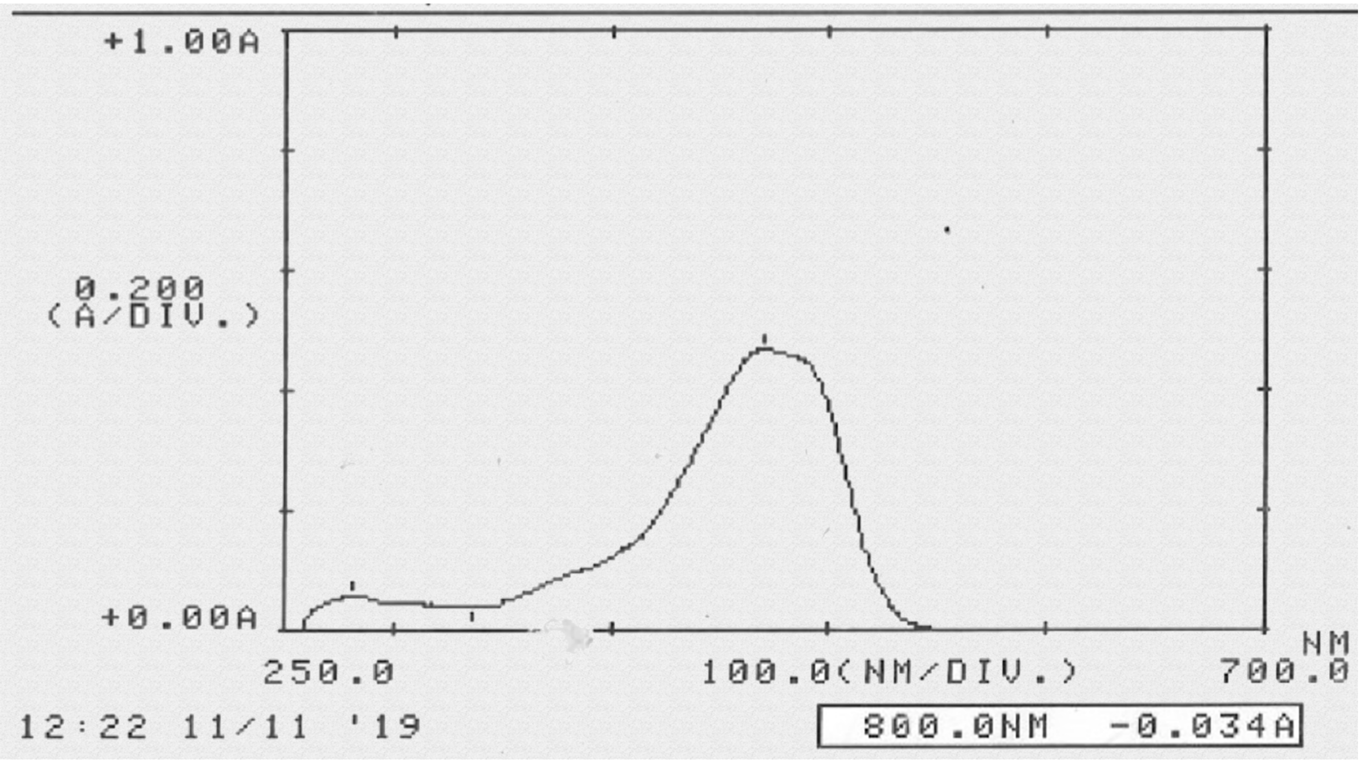

Figure S58. UV-Vis spectra of N-methyl-pyrCurc.

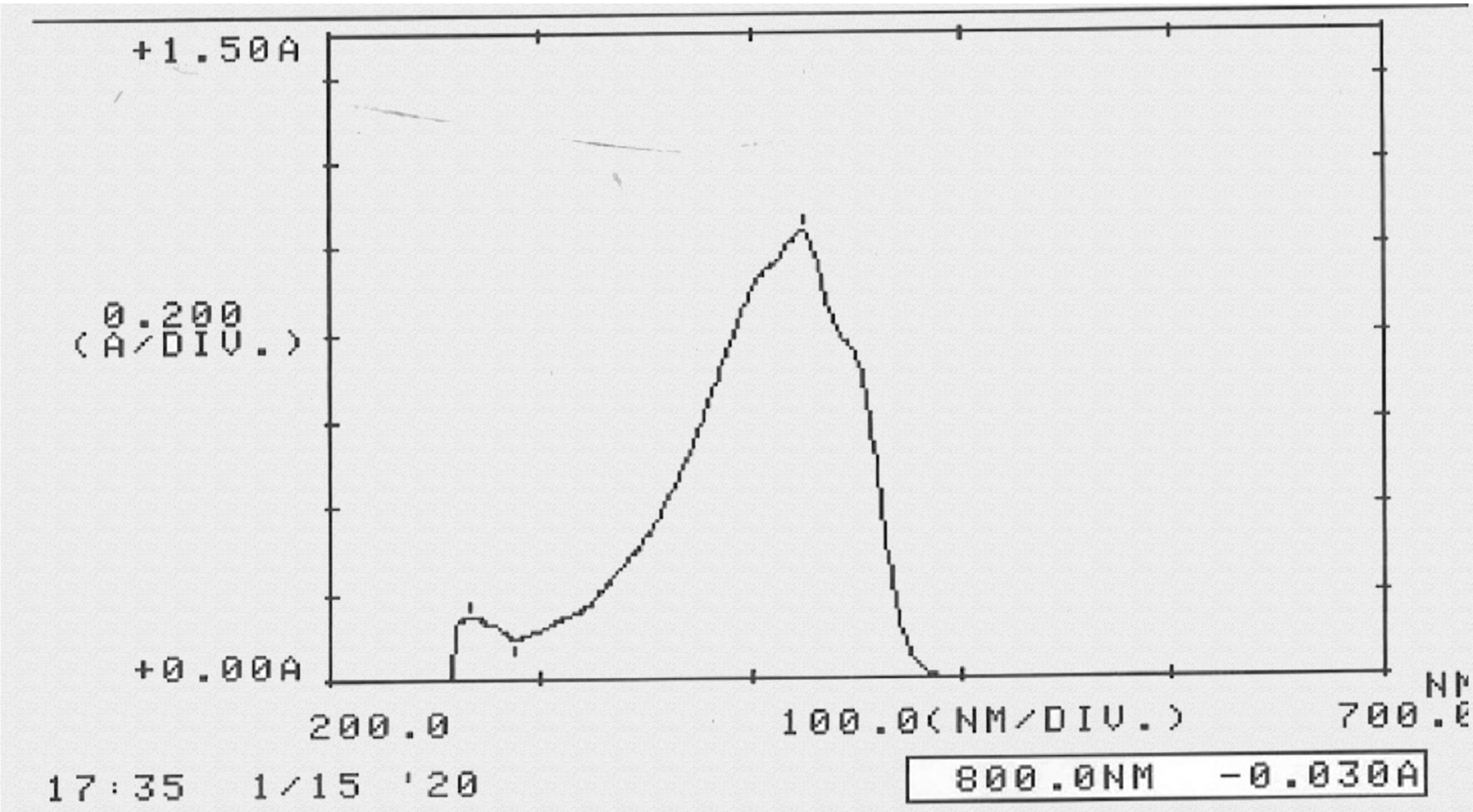

Figure S59. UV-Vis spectra of FuranCurc.

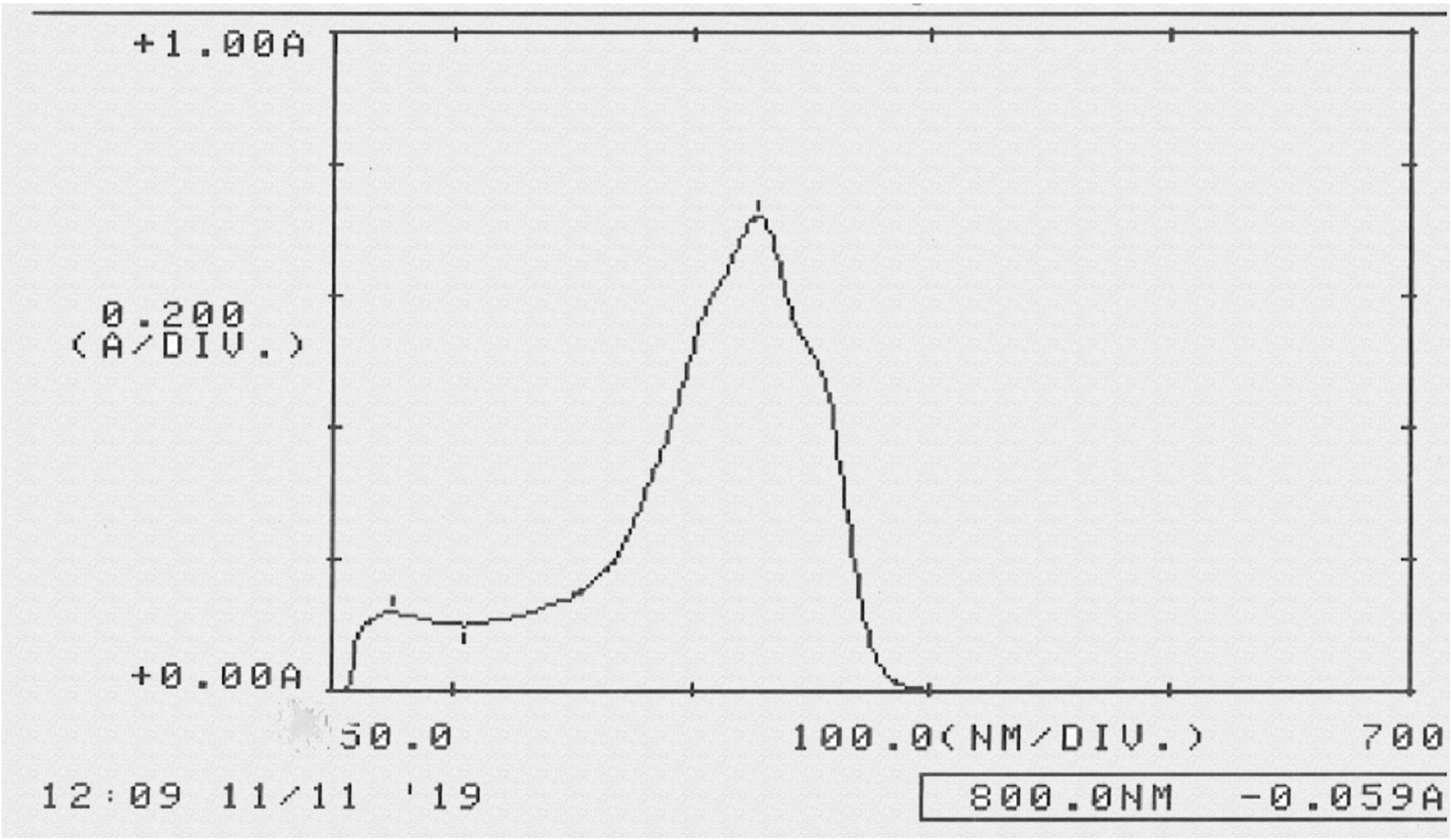

Figure S60. UV-Vis spectra of ThiopheneCurc.

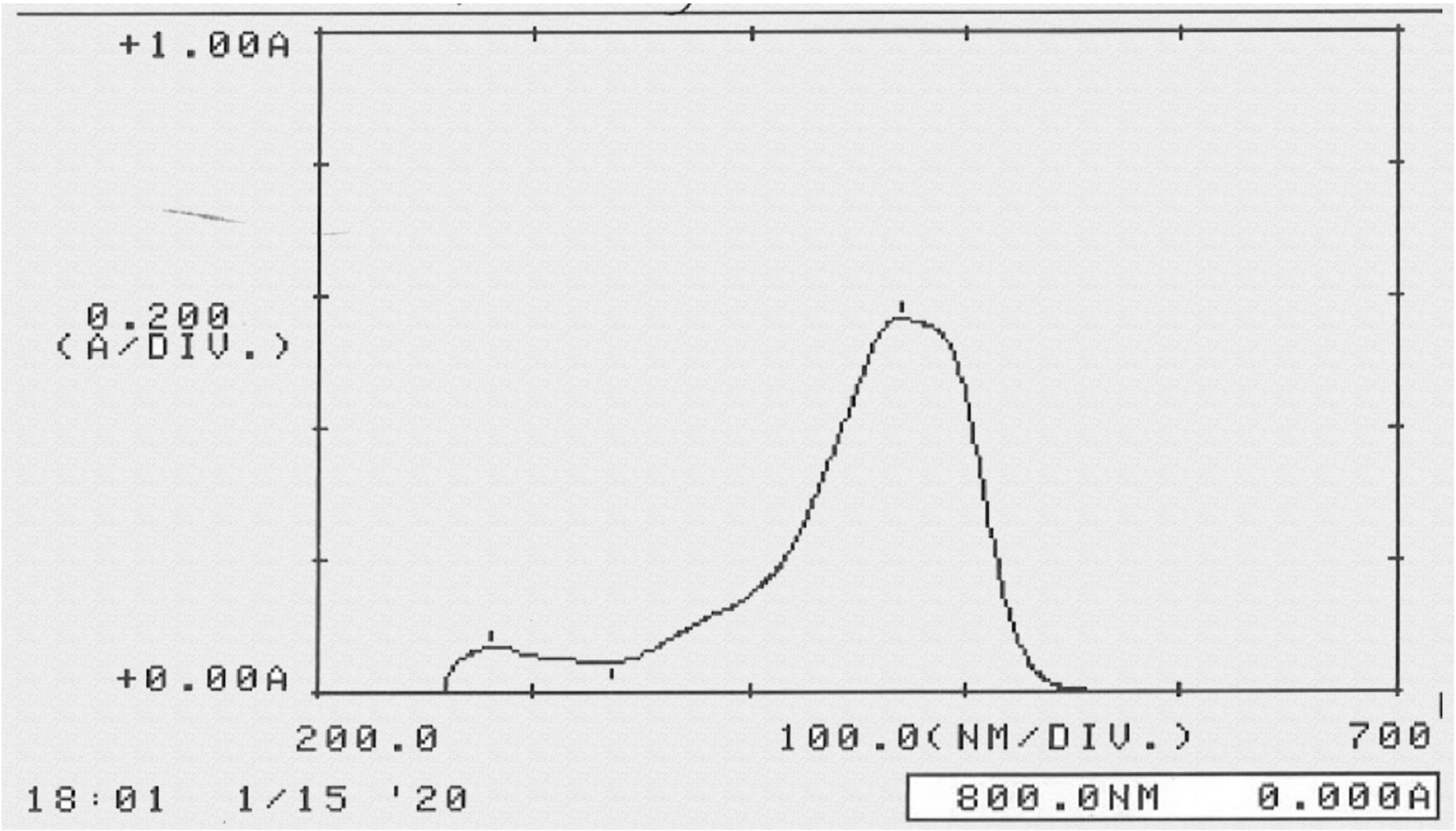

Figure S61. UV-Vis spectra of N-methyl-pyrCurc-Mg.

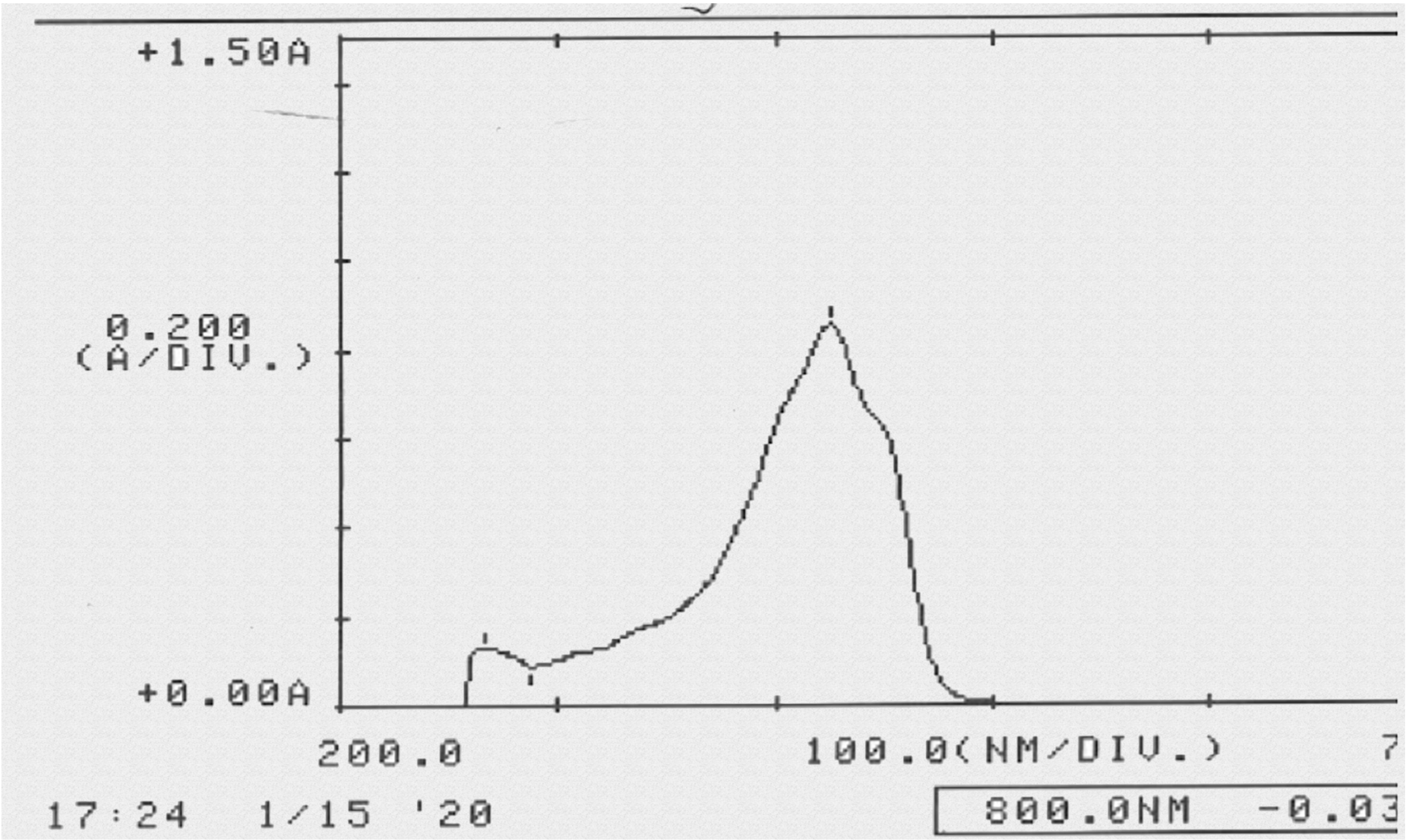

Figure S62. UV-Vis spectra of FuranCurc-Mg.

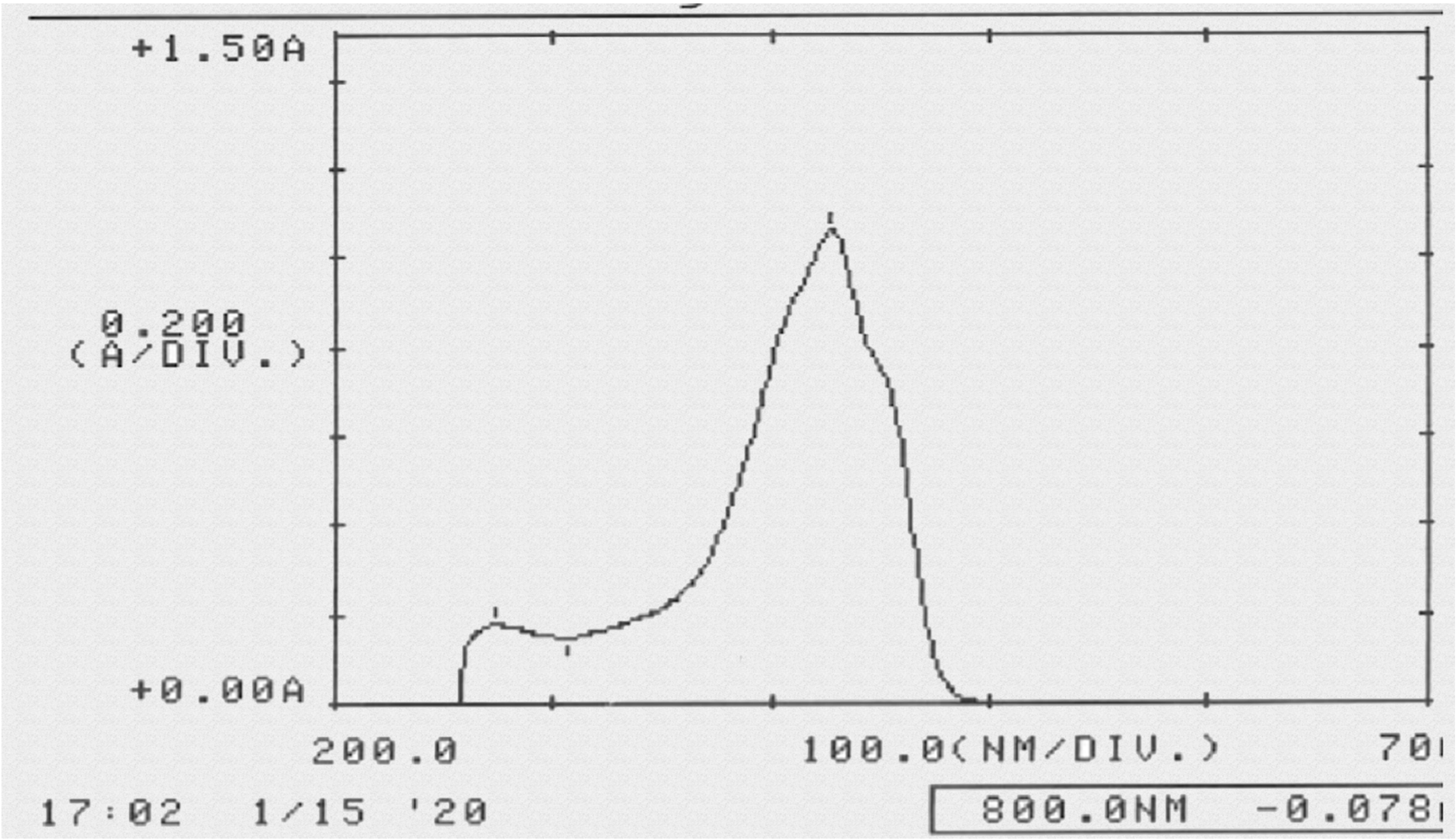

Figure S63. UV-Vis spectra of ThiopheneCurc-Mg.

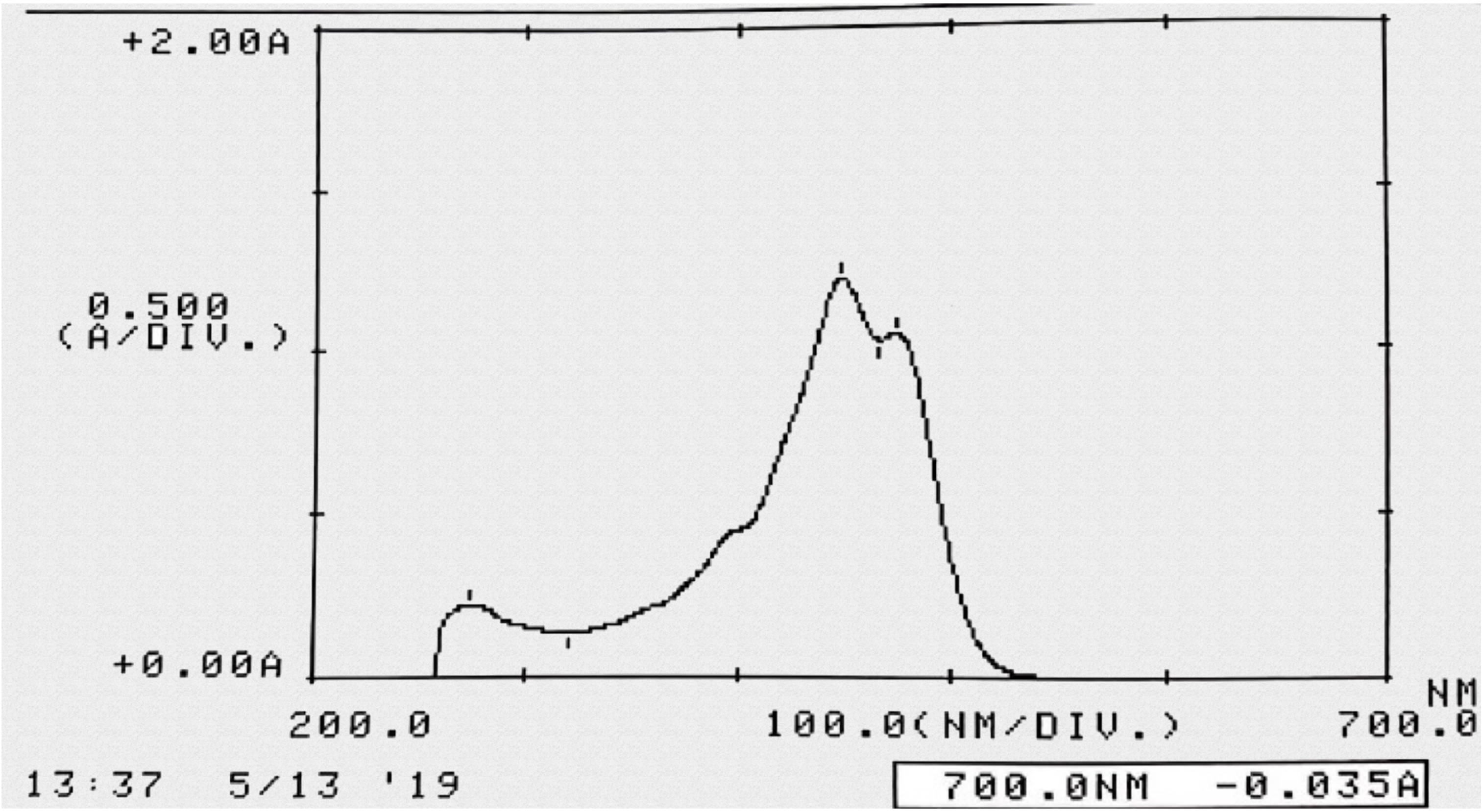

Figure S64. UV-Vis spectra of N-methyl-pyrCurc-Cu.

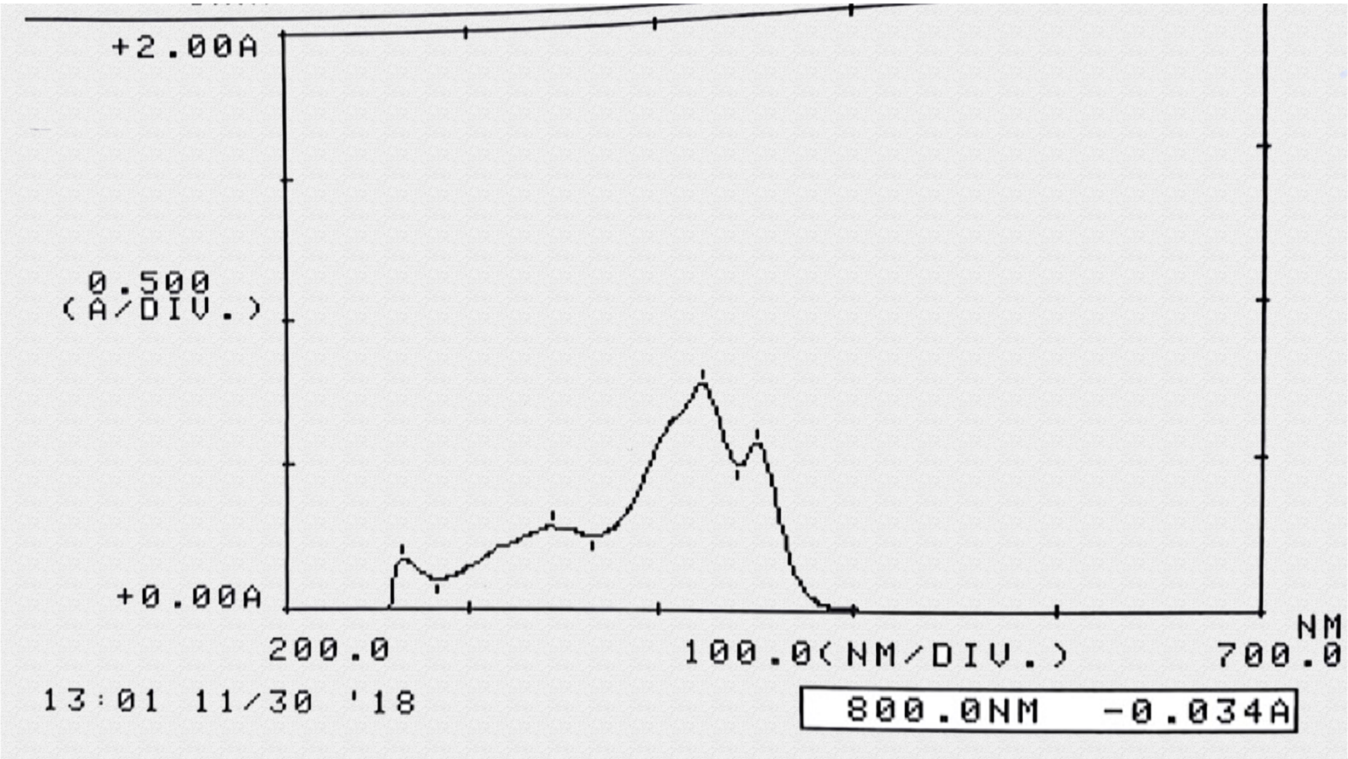

Figure S65. UV-Vis spectra of FuranCurc-Cu.

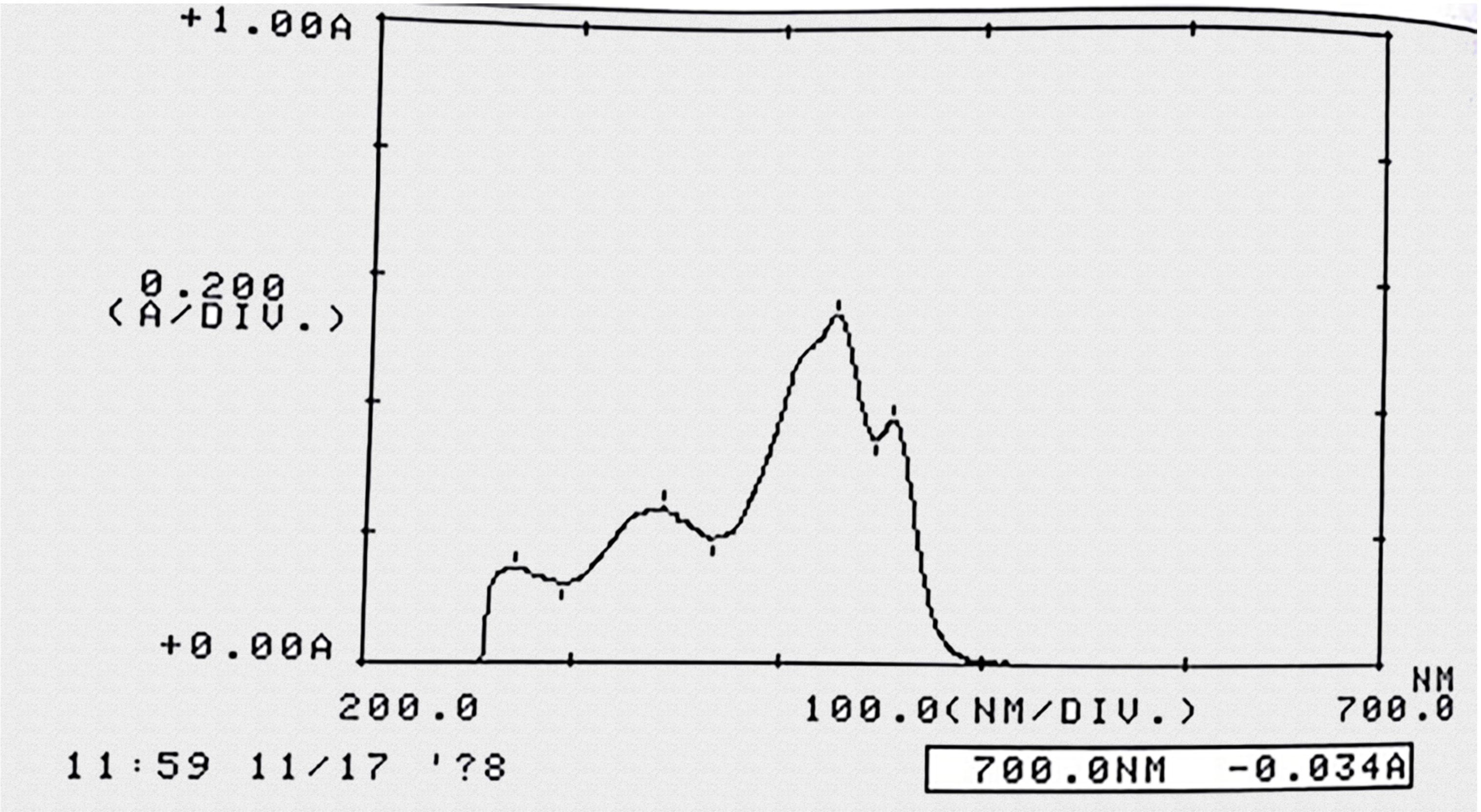

Figure S66. UV-Vis spectra of ThiopheneCurc-Cu.
